# Harnessing Light for G-Quadruplex Modulation: Dual Isomeric Effects of an *Ortho*-Fluoroazobenzene Derivative

**DOI:** 10.1021/acs.jpclett.4c02285

**Published:** 2024-09-17

**Authors:** Marta Dudek, Lucía López-Pacios, Nasim Sabouri, Juan J. Nogueira, Lara Martinez-Fernandez, Marco Deiana

**Affiliations:** 1 Institute of Advanced Materials, Faculty of Chemistry, Wrocław University of Science and Technology, Wyb. Wyspiańskiego 27, 50-370 Wrocław, Poland; 2 Departamento de Química, Facultad de Ciencias, Universidad Autónoma de Madrid, Campus de Excelencia UAM-CSIC, Cantoblanco, 28049 Madrid, Spain; 3 Department of Medical Biochemistry and Biophysics, Umeå University, SE-901 87 Umeå, Sweden; 4 Institute for Advanced Research in Chemistry (IAdChem), Universidad Autónoma de Madrid, Campus de Excelencia UAM-CSIC, Cantoblanco, 28049 Madrid, Spain; 5 Departamento de Química Física de Materiales, Instituto de Química Física Blas Cabrera, CSIC, 28006 Madrid, Spain

## Abstract

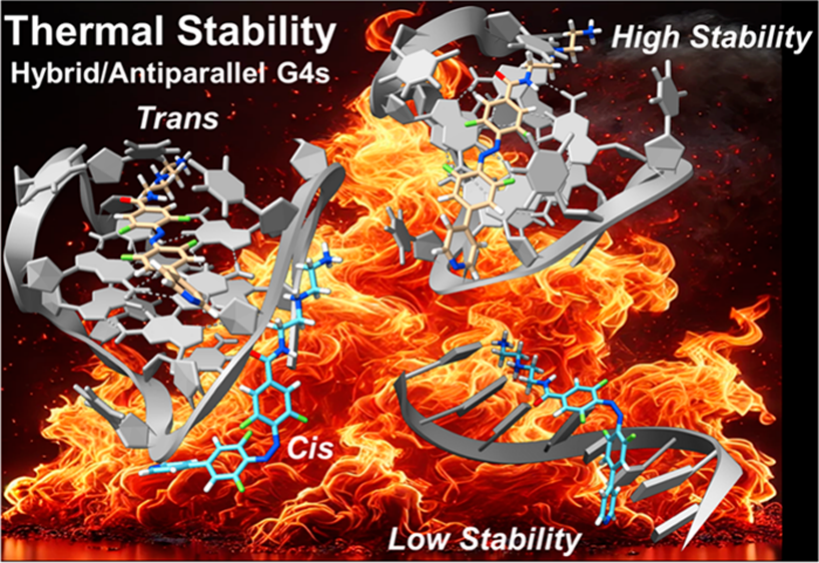

G-quadruplexes (G4s) are important therapeutic and photopharmacological targets in cancer research. Small-molecule ligands targeting G4s offer a promising strategy to block DNA transactions and induce genetic instability in cancer cells. While numerous G4-ligands have been reported, relatively few examples exist of compounds whose G4-interactive binding properties can be modulated using light. Herein, we report the photophysical characterization of a novel *ortho*-fluoroazobenzene derivative, **Py-Azo4F-3N**, that undergoes reversible two-way isomerization upon visible light exposure. Using a combination of biophysical techniques, including affinity and selectivity assays, structural and computational analysis, and cytotoxicity experiments in cancer cell lines, we carefully characterized the G4-interactive binding properties of both isomers. We identify the *trans* isomer as the most promising form of interacting and stabilizing G4s, enhancing their ablation capability in cancer cells. Our research highlights the importance of light-responsive molecules in achieving precise control over G4 structures, demonstrating their potential in innovative anticancer strategies.

Over the past decade, research focusing on the control of nucleic acid structure and functions has seen significant growth, with a multitude of reported DNA-based ligands, including molecular switches.^1−3^ Among various DNA structures, guanine-rich sequences forming four-stranded G-quadruplex (G4) structures^4^ have become an object of interest across various disciplines as therapeutic targets,^4−6^ functional materials,^7,8^ and catalysts.^9,10^ G4s are involved in key cellular processes such as transcription, replication, repair, and telomere maintenance.^11^ Their elevated levels in cancer cells compared to noncancerous cells make them attractive targets in cancer research.^4,6,12^ Consequently, they have become a focus for small-molecule ligands aimed at controlling their structure and biological functions.^11,13^ In cells, the formation of stable and persistent G4 structures can hinder the processivity of polymerases responsible for DNA transactions (replication, transcription, and repair).^51^ This disruption results in DNA damage and activates the DNA damage response (DDR) machinery.^5^ Specifically, G4-stabilizing ligands can exacerbate this disruption, elevating DNA damage levels and leading to genomic instability, particularly in DDR-deficient cells.^6,14^ However, the majority of these G4 stabilizers lack sensitivity to external stimuli, maintaining constant activity upon binding. This raises concerns about potential and persistent side effects in healthy cells. An alternative approach involves developing “active G4-ligands”, which enable the regulation of G4 properties through external stimuli.^15−17^ Light is an ideal tool for noninvasive manipulation of biological pathways, offering precise spatial and temporal regulation.^1,18,19^ Two different approaches to confer light sensitivity to biological systems have been implemented. One of them involves chemically modifying DNA sequences with photochromic molecules.^1^ In this context, guanine-rich DNA sequences were covalently linked with photoswitches, enabling the photoregulation of G4 structures^20,21^ and the selective transport of potassium ions across the lipid membrane.^22^ However, this approach requires covalent structural modification of native DNA sequences to engineer unnatural functionalities into the biomolecules, which limits the potential applications of these systems. Reversible modulation of G4 properties through noncovalent approaches offers an alternative method to overcome the limitations of covalent DNA modifications, especially when G4 structures are used as therapeutic or photopharmacological targets.^1^ This concept was implemented by Zhou and colleagues, who have demonstrated the light-triggered folding of G4 structures using an azobenzene (AB) derivative,^23^ in test tube settings.^24^ However, these systems exhibited limited reversibility and relied on UV light for isomerization,^23,24^ posing constraints on their applicability in cellular systems.^25,26^ In view of this, Galan’s group employed stiff-stilbene^27−29^ and dithienylethene^30,31^ derivatives symmetrically modified with *N*-methylated pyridine, to reversibly modulate the properties of the ligand-G4 complexes (Figure 1A). Through biophysical studies conducted on synthetically obtained isomers of stiff-stilbene, the differences in their activity to G4 structures were observed.^28^ However, it was found that stiff-stilbene ligands were susceptible to oxidation when exposed to light, thus restricting their application as G4-photoswitches.^27^ In contrast, the dithienylethene derivative exhibited efficient reversible switching with visible light in both directions; however, the isomeric effect on ligand-induced G4 stabilization and binding affinity was found to be negligible.^30^ Overall, these findings indicate that these scaffolds either lack photoswitching abilities or do not exhibit an isomer-dependent response (Figure 1A).

**Figure 1:**
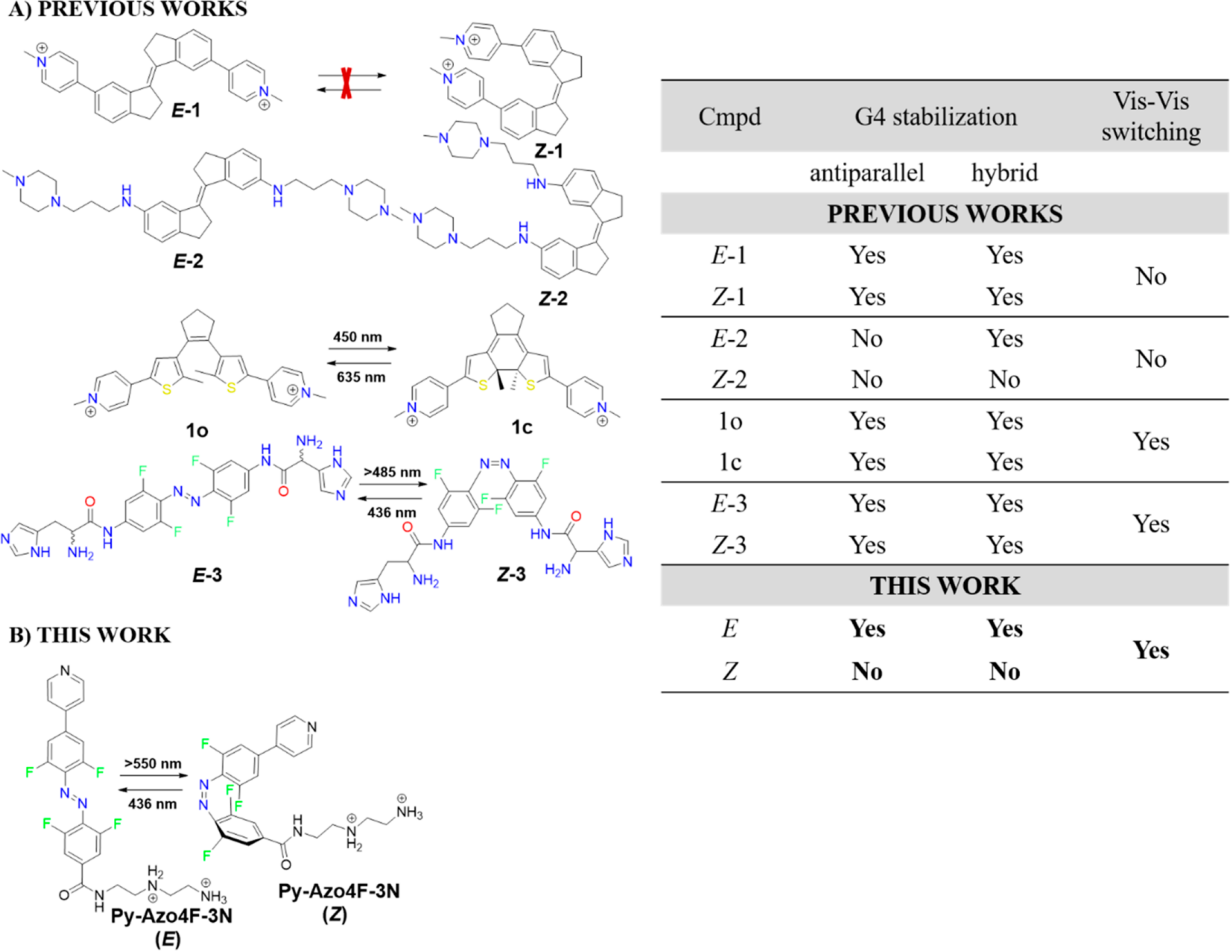
(A) Chemical structures of the previously studied isomers: *E*/*Z*-1;^27^*E*/*Z*-2;^28^ 1o/1c;^30^*E*/*Z*-3,^35^ interacting with G4s. The table summarizes insights into how isomers affect G4 stabilization, including both antiparallel and hybrid G4 topologies, as well as information about reversible switching under visible light irradiation. (B) Chemical structures of **Py-Azo4F-3N** in *trans* and *cis* forms, along with photoswitching.

As part of our investigation into the development of new G4 ligands,^17,32−34^ we become interested in the potential of *ortho*-fluoroazobenzene to act as photoresponsive G4-binding molecules.^35^ We speculated that significant geometrical variances between isomers and thus differences in properties could profoundly impact activity displayed toward G4 structures. Our findings revealed that *ortho*-fluoroazobenzene derivatives symmetrically modified with flexible side-chains, such as l- or d-histidine, demonstrated a degree of selectivity in recognizing G4 structures with opposite chirality. However, the overlap of the n−π* transitions of both isomers resulted in only 56% *cis*-to-*trans* isomerization under visible light irradiation, thereby restricting the photochromic properties.^35^ Moreover, as observed for other photoisomers,^30^ both isomeric forms induced stabilization of the antiparallel and hybrid G4 structures with minor differences between isomers (Figure 1A).^35^ Based on this insight, we hypothesized that replacing one flexible side of *ortho*-fluoroazobenzene with a rigid moiety and the other side with a flexible aliphatic chain might enhance the isomeric effect on G4 structures.

In this Letter, we tested this hypothesis by designing and synthesizing **Py-Azo4F-3N**, an *ortho*-fluoroazobenzene derivative featuring a flexible polyamine side chain on one end and a rigid pyridine group on the other (Figure 1B), targeting G4 structures. In fact, this compound demonstrated nearly quantitative two-way isomerization upon exposure to visible light. The isomeric activity toward G4 templates was validated through a combination of biophysical and theoretical approaches applied to biologically relevant parallel, antiparallel, and hybrid G4 DNA sequences.^4,11^ For the first time, we show a clear isomeric effect on ligand-induced stabilization of the antiparallel and hybrid G4 structures. Moreover, cytotoxicity studies conducted on cervical cancer HeLa cells and osteosarcoma U2OS cells showed an isomeric-dependent toxicity effect, which positively correlated to the biophysical and computational studies. These results suggest that AB-based G4 ligands are promising tools for controlling G4 dynamics and are great candidates for the development of new light-activated anticancer therapies.

**Py-Azo4F-3N** was synthesized (Figure 1B and Scheme S1) employing Mill’s reaction as the foundational step, aiming to construct a photochromic motif. The AB derivative was subsequently tailored at *para* positions, with a polyamine chain on one side, through the utilization of standard peptide bond synthesis protocols, employing HATU as a coupling agent, while on the other side, a pyridine unit was introduced via a Suzuki coupling reaction (see Supporting Information pp S6–S9 for details concerning synthesis). Our molecular design strategy was based on the utilization of (i) the photochromic motif to implement responsiveness to light,^1^ (ii) the polyamine chain to enhance electrostatic interactions with the G4 backbone and increase solubility in the aqueous medium,^34,36,37^ and (iii) the pyridine group, which may potentially act as a polyfunctional anchorage via π-stacking and hydrogen-bonding.^38^ Following the work of Hecht and co-workers,^39,40^ the incorporation of fluorine atoms at the *ortho* positions relative to the azo bond resulted in the separation of the n−π* transitions of the *trans* and *cis* isomers of **Py-Azo4F-3N** by approximately 40 nm (Figure 2A), enabling *trans*-to-*cis* and *cis*-to-*trans* isomerization solely with visible light. Specifically, irradiation of **Py-Azo4F-3N** with λ ≥ 550 nm induced *trans*-to-*cis* isomerization, while exposure to 436 nm light favored the back reaction (Figure 2A). According to the studies conducted via ^1^H and ^19^F NMR spectroscopy, the photostationary state (PSS) mixture of **Py-Azo4F-3N** contained ∼92% of the *cis* form when irradiated with λ ≥ 550 nm and ∼92% of the *trans* form when irradiated with 436 nm (Figure 2B and Figures S5 and S6). Similar to the findings for the *cis* isomers of other *ortho*-fluoroazobenzene derivatives,^34,40^ the *cis* isomer of **Py-Azo4F-3N** also demonstrated high thermal stability (Figure 2C, Table S1) at 25 °C, with a half-life (τ_1/2_) of approximately 52 days (see Supporting Information pp S11).

**Figure 2:**
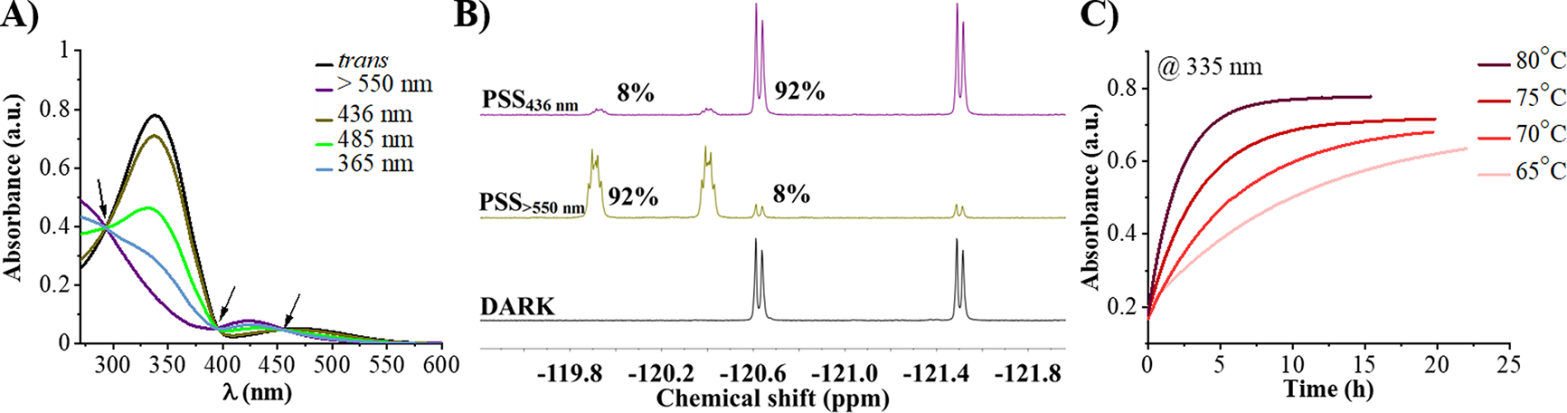
(A) Absorption spectra of **Py-Azo4F-3N** recorded in DMSO solution under excitation with light of different wavelengths (c_**Py-Azo4F-3N**_ = 30 μM). (B) PSS determination of **Py-Azo4F-3N** with ^19^F NMR spectroscopy (4.7 mM, in methanol-*d*_4_ at 25 °C) by the relative integration of the shifted signals. (C) The absorbance changes of 30 μM solution of **Py-Azo4F-3N** in DMSO were monitored at 335 nm during *cis*–*trans* thermal relaxation at four temperatures: 65, 70, 75, and 80 °C.

The controlled isomerization process with visible light, coupled with a high ratio of the isomers at PSSs and enhanced thermal stability of the *cis* isomer, prompted us to investigate the properties of two isomeric forms of **Py-Azo4F-3N** toward G4s.^11,41^ We selected a set of thoroughly characterized G4 structures, encompassing diverse topologies such as parallel, hybrid, and antiparallel (Table S2). First, we investigated the G4 binding affinity of the isomers of **Py-Azo4F-3N** by means of a ligand-induced fluorescence quenching assay with a 5′-fluorescently labeled *HIF-1α* G4 sequence using microscale thermophoresis (MST).^6,14^ Applied nonlinear curve-fitting procedures on MST traces for both isomers demonstrated robust binding affinity for the G4, with association constants of 2.8 × 10^5^ M^–1^ and 5.0 × 10^4^ M^–1^ for the *trans* isomer and *cis*-rich mixture, respectively (Figure 3A). The difference in the binding strength to the G4 between isomers prompted us to compare their ability to stabilize a variety of G4s and duplex (ds) DNA structures employing CD-based (or UV-based) thermal melting assay (Figure 3B and Supporting Information pp S13–S14).^34,42^ Experimental findings indicated that the planar *trans* isomer induced higher stabilization compared to the *cis* isomer, which features a bent geometry. **Py-Azo4F-3N**_*trans*_ stabilized all the tested G4 sequences being the most efficient for parallel G4s (*c-MYC* Pu22 and *VEGF*) increasing their melting temperature (*T*_m_) by 19.2 and 14.7 °C, respectively (Table S3). A lower stabilization effect was observed for the hybrid G4 (Tel22-K^+^), the antiparallel G4s (Tel22-Na^+^, Bom17, TBA), and other parallel G4s (*c-MYC* Pu24T) with the Δ*T*_m_ ranging from 4.2 to 9.9 °C (Figures 3B and Table S3). Importantly, under comparable experimental conditions, the duplex stabilization remains very low (Δ*T*_m_ ∼ 1.0 °C) in the presence of the *trans* isomer. The results provide initial evidence of a degree of selectivity for G4s over the duplex model. The same experiments performed for the G4s in the presence of the *cis*-rich mixture of **Py-Azo4F-3N** showed a much weaker influence on the *T*_m_ of *c-MYC* Pu22 and VEGF (7.4 and 4.4 °C, respectively), while a negligible impact on *T*_m_ for other DNA sequences was observed (<1.1 °C). These results show significant differences in the isomer-driven G4 stabilization, contrasting with the minor differences observed in other studies involving azobenzene^35^ or dithienylethene^30^ derivatives. To our knowledge, this is the first instance demonstrating isomeric-dependent activity toward ligand-induced G4 thermal stabilization, in particular for antiparallel and hybrid topologies, showing an ON-to-OFF response in the presence of the *trans* or *cis*-rich mixture of **Py-Azo4F-3N**, respectively (Figure 3B and Table S3).

**Figure 3:**
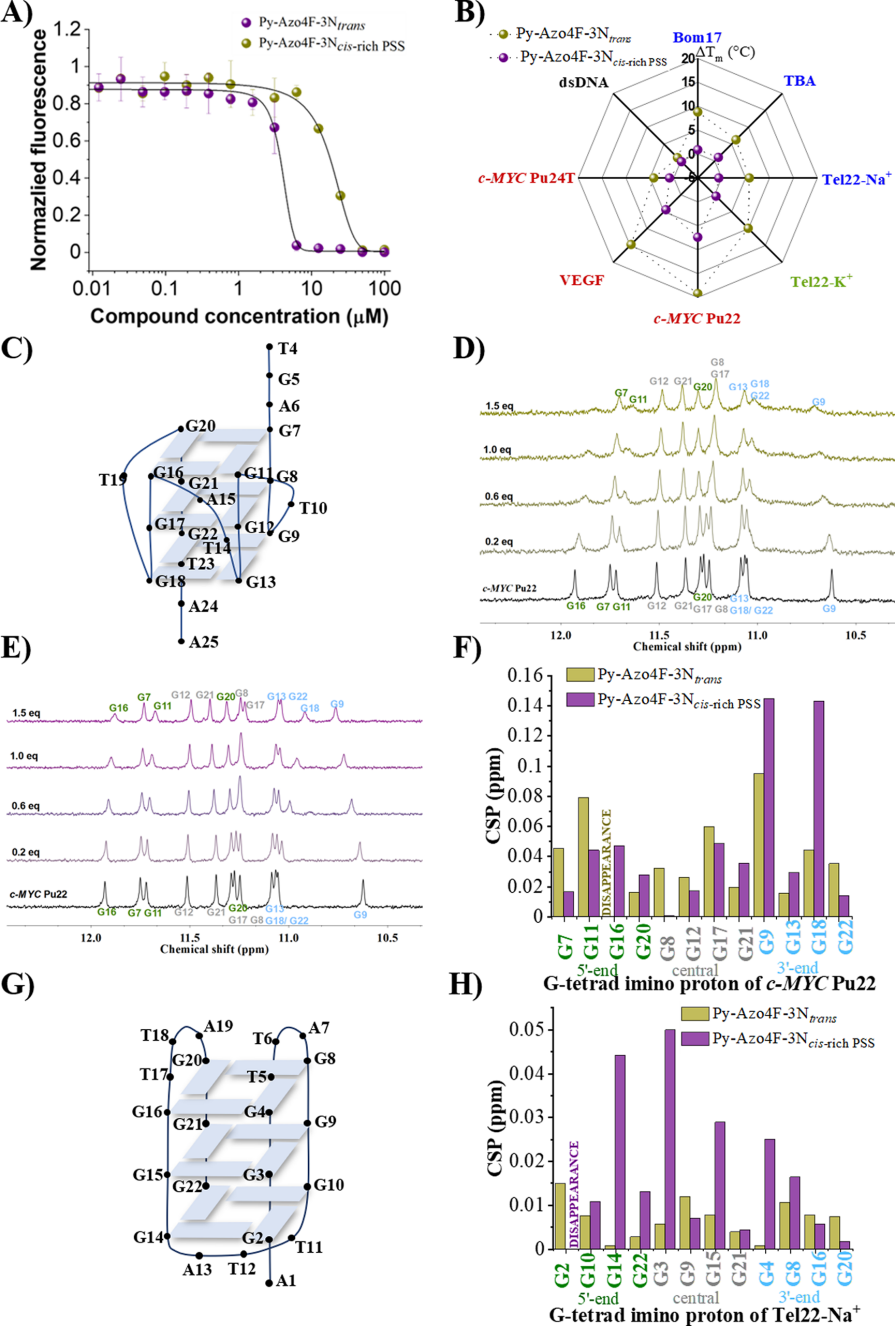
(A) MST binding curves for **Py-Azo4F-3N**_*trans*_/**Py-Azo4F-3N**_*cis*-rich PSS_ with Cy5-*HIF-1α* (c_G4_ = 50 nM, c_ligand_ = 3.05 nM to 100 μM, 100 mM KCl, 50 mM Tris pH = 7.2, 0.05% Tween 20). (B) Radar plot showing the ability of both isomers of **Py-Azo4F-3N** (8 μM) to stabilize different G4 structures (2 μM) and duplex dsDNA (2 μM). (C) Schematic representation of *c-MYC* Pu22 G4 formed in K^+^. (D, E) ^1^H NMR spectra of the G-tetrad imino protons in the absence (0.0 equiv) and presence (0.2, 0.6, 1.0, and 1.5 equiv) of **Py-Azo4F-3N**_*trans*_ (D) and of **Py-Azo4F-3N**_*cis*-rich PSS_ (E). (F) Plot of chemical shift perturbation (CSP) comparing *c-MYC* Pu22 imino protons alone and with the presence of 1.5 equiv of **Py-Azo4F-3N**_*trans*_ (olive) and **Py-Azo4F-3N**_*cis*-rich PSS_ (violet). (G) Schematic representation of Tel22 G4 formed in Na^+^. (H) Plot of CSP comparing Tel22-Na^+^ imino protons alone and with the presence of 2.0 equiv of **Py-Azo4F-3N**_*trans*_ (olive) and **Py-Azo4F-3N**_*cis*-rich PSS_ (violet).

To confirm the ability of **Py-Azo4F-3N**_***trans***_ to discriminate between quadruplex and duplex DNA, we performed FRET melting experiments with F-Tel22-Na^+^-T under competitive conditions with increasing amounts of dsDNA (Figure S10). No significant alteration in the thermal stability of Tel22-Na^+^ G4 DNA induced by **Py-Azo4F-3N**_*trans*_ was observed, demonstrating its selectivity for G4 over that of duplex DNA.

Based on these findings, we aimed to investigate the nature of the interactions between the isomers and G4 topologies. To do so, we performed CD and ^1^H NMR studies coupled with theoretical calculations. The CD titration experiments performed with G4s and both *trans* and *cis*-rich mixtures of **Py-Azo4F-3N** revealed no significant changes in the G4 structure upon binding (Figures S11–S14). To provide further structural insights into the interactions of **Py-Azo4F-3N** in both geometries with parallel (*c-MYC* Pu22) and antiparallel (Tel22-Na^+^) G4 structures, we performed ^1^H NMR titration experiments (Figures 3C–H and S15–S18). The free *c-MYC* Pu22 forms a single G4 conformation as indicated by 12 well-resolved guanine imino proton peaks (Figure 3D,E).^42−44^ Upon the addition of **Py-Azo4F-3N**_*trans*_, the progressive broadening and attenuation, especially G16 and G11 resonances from the 5′-end as well as G9 and G18/G22 signals from 3′-end was observed (Figure 3D,F). This suggests that interactions with this part of the G4 are important in the binding of ligand **Py-Azo4F-3N**_*trans*_. Importantly, significant chemical shift perturbation was observed for G17 indicating some interactions with central G-tetrads. Contrary, the titration of the *c-MYC* Pu22 with *cis*-rich mixture of **Py-Azo4F-3N** shows fast exchange chemical shift perturbations, allowing all 12 guanine imino protons from the G-tetrad planes to be tracked (Figure 3E,F).^45^ The ligand induced chemical shift perturbations significantly for 3′-end resonances (G9 and G18) and central resonances (G17 and G21). Since G17 is located above G18, we speculate that the ligand’s arms can be sandwiched between these two G4-tetrads affecting both the guanines. Finally, we performed an isomerization to confirm the reversibility of the binding mode to the G4 template (Figure S15). The *cis*-to-*trans* isomerization, triggered by irradiation with 436 nm caused the broadening of imino proton signals together with chemical shift perturbations of G8, G17, and G18 as well as attenuation of G9, G11, and G16 resonances particularly observed for *c-MYC* Pu22 treated with **Py-Azo4F-3N**_*trans*_. Nevertheless, the *trans*-to-*cis* isomerization did not reverse the changes linked with the formation of the *cis*-*c*-*MYC* Pu22 complex (Figure S15). This is probably because the *trans* isomer tends to stay firmly bound within the G4 structure, reflecting its strong binding affinity. In general, these results suggest some degree of reversibility in the process although not bidirectional.

To further assess how **Py-Azo4F-3N** interacted with antiparallel G4 DNA, NMR titrations were carried out using the Tel22-Na^+^ G4 structure (Figure 3G,H), known for its distinct single, well-defined basket topology (Figures S16–S18).^46,47^ Noticeable line broadening and attenuation of the imino resonances were observed during titration with **Py-Azo4F-3N**_*trans*_ (Figures 3H and S16). All imino signals broaden to a similar degree, suggesting that interactions with specific G-tetrad residues do not dominate in the association of the ligand with G4. Importantly, the titration of G4 with a *cis*-rich mixture of **Py-Azo4F-3N** resulted in notable changes, such as the chemical shift of G14 and the disappearance of G2, both of which are associated with the 5′-end (Figures 3H and S17). The chemical shift perturbations were also observed for G3 and G15 resonances from the central G-tetrad, with G3 and G15 positioned above G2 and G14, respectively, indicating the ligand’s localization between those two G-tetrads. Finally, we performed *in situ* photoisomerization of Tel22-Na^+^-**Py-Azo4F-3N**_*cis*-rich PSS_ triggered with 436 nm of light irradiation, revealing the reversibility of the binding mode to the G4 template (Figure S18). This was primarily evidenced by the chemical shift perturbation of G14 and the reappearance of the G2 resonances, which had been diminished in the presence of the *cis* isomer.

In order to examine whether duplex DNA may alter the coordination mode of the photochrome to G4s, we conducted ^1^H NMR competitive binding experiments with *c-MYC* Pu22 under increasing concentrations of dsDNA (Figures S19 and S20). No significant change was observed in the chemical shift perturbations of *c-MYC* Pu22-**Py-Azo4F-3N**_*trans*/*cis-*rich PSS_ complexes, ruling out any interference from duplex DNA.

The experimental findings of the binding mode were further investigated based on a computational approach (see Supporting Information pp S20–S48). We performed molecular docking and classical molecular dynamics (MD) simulations to investigate the binding modes of **Py-Azo4F-3N** into *c-MYC* Pu22 and Tel22-Na^+^. Using the *c-MYC* Pu22 (PDB ID 1XAV)^44^ and Tel22-Na^+^ (PDB ID 143D)^46^ PDB structures as docking target receptors, we identified the main external poses for both *cis* and *trans* isomers. In addition, we investigated the intercalative poses using *c-MYC* Pu22 and Tel22-Na^+^ structures that were previously opened through a combination of MD and umbrella sampling calculations (see section 6 in the Supporting Information for more details). Then, we run MD combined with free energy calculations for the two most populated external poses and the most populated intercalative pose. In the case of the *cis* isomer, we run a MD simulation for an additional pose to explore the intercalation of both sides of **Py-Azo4F-3N**: the pyridine and the aliphatic amine. Table 1 shows the free energy results for the most stable external and intercalated binding modes. In addition, the structures of the most stable complexes for both G4s are shown in Figure 4A,B,D,E.

**Table 1:** Molecular Mechanics Generalized Born Surface Area (MMGBSA)^52^ Binding Free Energies in kcal/mol of the Most Stable External and Intercalative Binding Pockets of **Py-Azo4F-3N**_*trans*_ and **Py-Azo4F-3N**_*cis*_ in *c-MYC* Pu22 and Tel22-Na^+^

	*trans*_ext_	*trans*_int_	*cis*_ext_	*cis*_int_
*c-MYC* Pu22	–81.0	–121.4	–84.1	–89.6
Tel22-Na^*+*^	–65.3	–109.5	–82.8	–105.2

**Figure 4:**
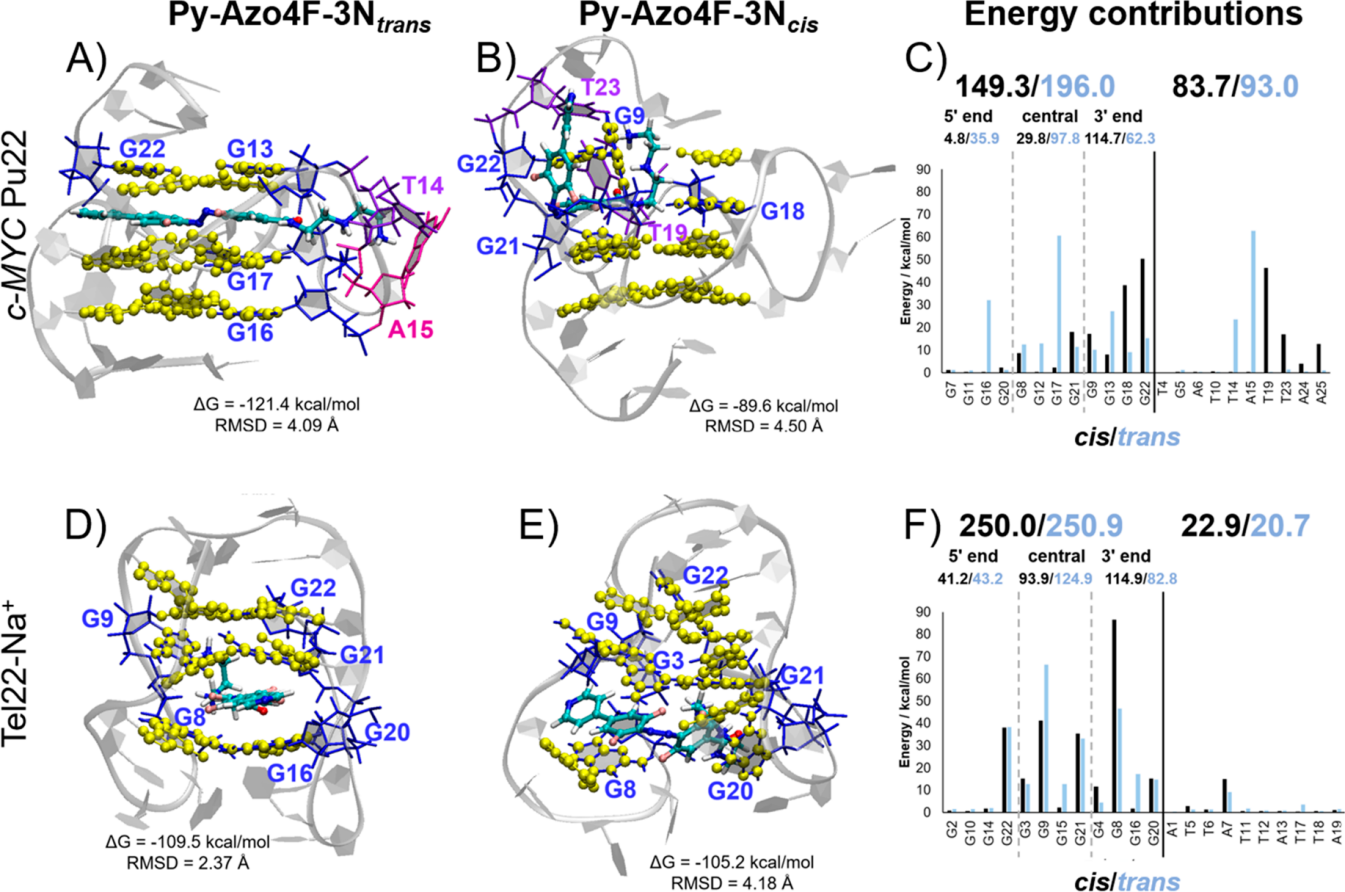
Representative structures of the most stable binding modes of **Py-Azo4F-3N**_*trans*_ in *c-MYC* Pu22 (A) and Tel22-Na^+^ (D) and **Py-Azo4F-3N**_*cis*_ in *c-MYC* Pu22 (B) and Tel22-Na^+^ (E), along with their corresponding MMGBSA binding energy in kcal/mol (absolute value) and their RMSD with respect to the initial G4 structure. The decomposition of the energy contributions per nucleotide is represented for *c-MYC* Pu22 (C) and Tel22-Na^+^ (F).

Upon comparison of the free energies of the most stable poses (Table 1), one can conclude that the interaction of the *trans* isomer is stronger than the corresponding *cis* isomer for both G4s: −121.4 vs −89.6 and −109.5 vs −105.2 kcal/mol for *c-MYC* Pu22 and Tel22-Na^+^, respectively. This qualitatively agrees with the obtained MST association constants and the Δ*T*_m_. Furthermore, the weaker stabilization effect for the antiparallel Tel22-Na^+^ G4 with **Py-Azo4F-3N**_*trans*_ may be related to its weaker interaction energy (−109.5 for Tel22-Na^+^ vs −121.4 kcal/mol for *c-MYC* Pu22).

According to these results, the intercalative pose is energetically favored for the *trans* isomer in both G4s. The energy decomposition analysis (Figure 4C,F) shows that the intercalation is dominated by the interaction with the G tetrads (196.0 kcal/mol for *c-MYC* Pu22, 250.9 kcal/mol for Tel22-Na^+^), with significantly smaller contributions from the external nucleobases (93.0 kcal/mol for *c-MYC* Pu22, 20.7 kcal/mol for Tel22-Na^+^). Note that the energies of the decomposition analysis are expressed in absolute values. In both cases, the most relevant interactions are with the central tetrad (97.8 kcal/mol for *c-MYC* Pu22, 124.9 kcal/mol for Tel22-Na^+^), although, in accordance with the NMR experiments, the contributions from the 5′ and 3′ ends are significant too. Intercalation is also favored for the *cis* isomer, although it may coexist at equilibrium with the external binding in *c-MYC* Pu22, which is only 5.5 kcal/mol less stable (see Table 1). In the same way, as for the *trans* isomer, the G tetrads energy contributions are much more relevant than the external nucleobases (149.3 vs 83.7 kcal/mol for *c-MYC* Pu22, 250.0 vs 22.9 kcal/mol for Tel22-Na^+^), as shown in Figure 4C,F. In this case, the most important contributions come from the 3′-end (114.7 kcal/mol for *c-MYC* Pu22, 114.9 kcal/mol for Tel22-Na^+^), which corresponds to the end where we manually placed the intercalated **Py-Azo4F-3N** species.

Finally, as shown by the experimental data, the MD simulations (Figure 4) also suggest that **Py-Azo4F-3N**_*trans*_ leads to more potent G4 stabilization than **Py-Azo4F-3N**_*cis*_. The G4 structure is well preserved when **Py-Azo4F-3N**_*trans*_ intercalates, probably due to the π–π stacking between **Py-Azo4F-3N** and the G tetrads. On the other hand, **Py-Azo4F-3N**_*cis*_ intercalation induces a partial tetrad unfolding in both G4s, with a more noticeable effect on Tel22-Na^+^. This can be quantitatively measured by the calculation of the root mean square deviation (RMSD) of the binding representative structures with respect to the initial isolated G4 structures. The RMSD of the tetrad nucleobases when **Py-Azo4F-3N**_*trans*_ intercalates (4.09 Å for *c-MYC* Pu22 and 2.37 Å for Tel22-Na^+^) is lower than when **Py-Azo4F-3N**_*cis*_ intercalates (4.50 Å for *c-MYC* Pu22 and 4.18 Å for Tel22-Na^+^), indicating a greater G4 distortion upon **Py-Azo4F-3N**_*cis*_ intercalation. Finally, this is also consistent with the IC_50_ values that we show below, in which we demonstrate that **Py-Azo4F-3N**_*trans*_ is more cytotoxic, presumably due to the stabilization of the G4 structures.

We speculated that the difference in the interaction between the AB photoisomers and G4s, as it was previously observed for other photochromic molecules,^30^ may influence toxicity toward cancer cell lines.^48,49^ Therefore, we performed cytotoxicity studies of both isomers of **Py-Azo4F-3N** in cervical cancer HeLa cells and osteosarcoma U2OS cells (Figure 5).^50^ A difference of about 2-fold in cytotoxicity was noted in both cancer cell lines between the two different isomeric states, indicating that this effect varies based on the switch’s conformation. The **Py-Azo4F-3N**_*trans*_ exhibited higher toxicity compared to the *cis*-rich mixture of **Py-Azo4F-3N**, and the half-maximum inhibitory concentrations (IC_50_) were approximately 6 and 13 μM, respectively. These data suggest that G4 stabilization may be involved in mediating cancer cell death upon binding of the photochrome. However, it is essential to note that these are preliminary findings, and alternative therapeutic mechanisms cannot be entirely ruled out at this stage.

**Figure 5:**
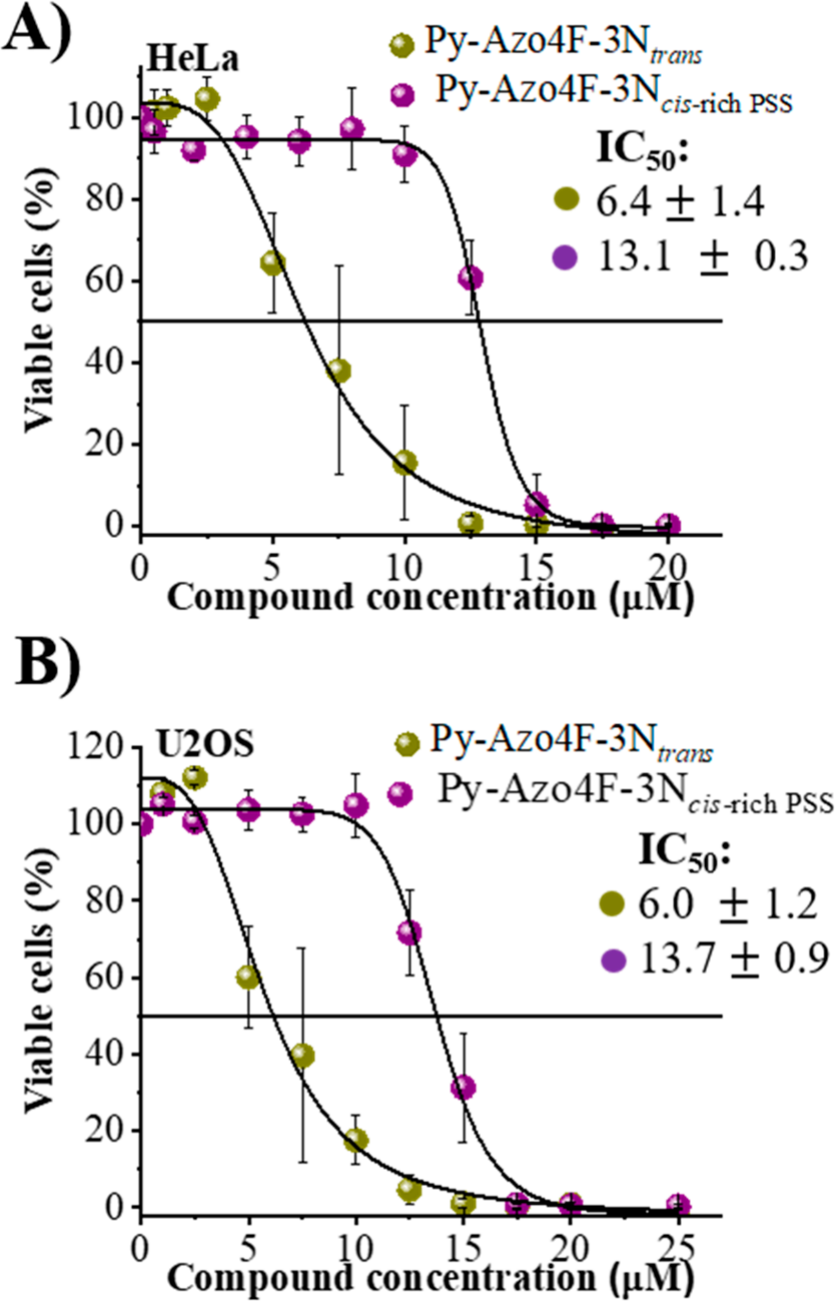
Cytotoxicity of **Py-Azo4F-3N** on HeLa (A) and U2OS (B) cell lines using both *trans* and *cis*-rich PSS, after 48 h of incubation. Error bars indicate the mean ± SD (*n* = 3).

In summary, we have developed a photochromic G4-targeted ligand based on the *ortho-*fluoroazobenzne core, which allows isomerization solely with visible light in both directions, achieving a satisfactory isomer ratio of 92% and 8% at PSS. Both isomers interact with the G4s, but the binding strength to the G4 template is higher for the *trans* isomer than for the *cis* isomer. This difference can be attributed to their distinct geometries. The *trans* isomer, which is nearly flat, facilitates π-stacking interactions with the G-tetrads, whereas these interactions may be partially impeded in the presence of the *cis* isomer due to its bent geometry. Importantly, for the first time, we have demonstrated that only the *trans* isomer stabilizes hybrid and antiparallel G4 structures. This enhanced isomeric effect surpasses that of previously studied photochromic G4-binders and may result from different binding modes of the photoisomers on the G4 template, as revealed through CD, ^1^H NMR, and theoretical calculations. Additionally, the strong G4 binding and stabilization by the *trans* isomer translate into increased toxicity toward human cancer cell lines, suggesting a potential mechanism of action involving genomic G4 sequences. These findings open new avenues for developing light-activated compounds aimed at regulating G4-associated biological processes.

## Supporting Information Available

Experimental procedures, synthesis and characterization of the compounds, photoisomerization and thermal stability studies, DNA-binding studies, and computational details and results (PDF)
