# Supplementary material for: Harnessing Light for G-Quadruplex Modulation: Dual Isomeric Effects of an Ortho-Fluoroazobenzene Derivative
Source: J Phys Chem Lett. 2024 Sep 17;15(38):9757–65. doi: 10.1021/acs.jpclett.4c02285 (PMC11440583; doi:10.1021/acs.jpclett.4c02285)
Supplement: Supplementary file 1 — jz4c02285_si_001.pdf [file jz4c02285_si_001.pdf]

Supporting Information

Harnessing Light for G-Quadruplex Modulation: Dual Isomeric Effects of an *Ortho*-Fluoroazobenzene Derivative

Marta Dudek[1], Lucía López-Pacios[2], Nasim Sabouri[3], Juan J. Nogueira[2,4], Lara Martinez-Fernandez[5] and Marco Deiana[1,3]*

[1]Institute of Advanced Materials, Faculty of Chemistry Wrocław University of Science and Technology Wyb. Wyspiańskiego 27, 50-370 Wrocław, Poland.

[2]Departamento de Química, Facultad de Ciencias, Universidad Autónoma de Madrid, Campus de Excelencia UAM-CSIC, Cantoblanco, 28049 Madrid, Spain.

[3]Department of Medical Biochemistry and Biophysics Umeå University SE-901 87 Umeå, Sweden.

[4]Institute for Advanced Research in Chemistry (IAdChem), Universidad Autónoma de Madrid, Campus de Excelencia UAM-CSIC, Cantoblanco, 28049 Madrid, Spain.

[5]Departamento de Química Física de Materiales, Instituto de Química Física Blas Cabrera, CSIC; 28006, Madrid, Spain.

*E-mail: marta.ziemianek-dudek@pwr.edu.pl

Table of Contents:

1. Materials and methods S3
2. Synthesis and characterization S6
3. Photoisomerization studies S10
4. Thermal stability S11
5. DNA studies S12
    5.1 DNA characterization S12
    5.2 Melting studies S13
    5.3 G4 DNA conformational changes S15
    5.4 NMR studies S17
6. Theoretical calculations S20
    6.1 Umbrella sampling S20
    6.2 Docking calculations S22
    6.3 Molecular dynamics simulations S29
7. References S48

1. MATERIALS AND METHODS

Solvents and reagents were purchased from commercial suppliers and used as received. Column chromatography was carried out using a flash chromatograph equipped with RediSep columns filled with silica gel (particle size: 40–63 μm irregular, mesh size: 230–400). Unless otherwise specified, all reactions were conducted under standard conditions. Oligonucleotides were obtained from MERCK.

NMR spectra were acquired using a Bruker AvanceTM 600 MHz spectrometer or a JEOL 400 MHz spectrometer at 25 °C. Residual protonated solvent signals were used as internal standards for ^{1}H and ^{13}C spectra. Multiplicities are denoted as follows: singlet (s), doublet (d), triplet (t), multiplet (m), and broad (br).

High Pressure Liquid Chromatography (HPLC) was done using Waters 1525 solvent delivery module with a Waters M2489 detector system equipped with a semipreparative C18 column (SepaChrom, 300 Å, 10 μ, 250 x 10 mm) in water/acetonitrile (0.05% TFA).

High resolution mass spectra (HRMS) were conducted with a WATERS LCT Premier XE mass spectrometer (ESI).

Absorption spectra were obtained using a JASCO V-730 spectrometer equipped with a JascoPeltier-type temperature controller (CDF-426S/15). All optical measurements were conducted in quartz cell cuvettes with standard path lengths of 10 mm.

Sample irradiation was performed using a UV Spot Light Source (Hamamatsu Photonics K.K., model: L9588-04) equipped with filters: 365 nm, 436 nm, 485 nm, and ≥ 550 nm.

The thermal stability of the *cis* isomer was evaluated by monitoring absorbance changes at various temperatures using a JASCO V-730 spectrometer equipped with a JascoPeltier-type temperature controller (CDF-426S/15). Initially, the solution containing 30 μM of **Py-Azo4F-3N** in DMSO was irradiated with λ ≥ 550 nm for 10 minutes to induce *trans*-to-*cis* isomerization. Subsequently, absorbance readings were recorded in the range of 250-600 nm over time at specific temperatures of 65 °C, 70 °C, 75 °C, and 80 °C. The half-life of the *cis* isomer at room temperature was calculated using the Arrhenius equation $\ln k = \ln A - E_a/RT$.

The composition of the photostationary state (PSS) was conducted on **Py-Azo4F-3N** (3.3 mM in methanol-d4 at 25 °C) using a UV Spot Light Source equipped with filters: 436 nm, 485 nm, and ≥550 nm. The NMR sample was irradiated for 15 minutes before recording each NMR spectrum. The determination of PSSs compositions was conducted via ^{1}H and ^{19}F NMR spectroscopy, with the isomeric ratio derived from intensity comparisons of the corresponding peaks.

Microscale thermophoresis (MST) experiments were conducted using a Monolith NT.115 (NanoTemper, Germany) with 50% LED power in standard MST-grade glass capillaries. The *HIF-1α* DNA labeled with Cy5 at the 5′-end (c_{G4}= 50 nM, c_{TRIS} = 50 mM, pH = 7.2, c_{KCl} = 100 mM, 0.05% Tween 20) was titrated with a **Py-Azo4F-3N** *trans* and *cis*-rich mixture ranging from 3 nM to 100 μM (16 dilution steps). The dissociation constant (Kd) was calculated by fitting the data to the Hill equation using OriginPro 2020b.

Melting studies were conducted using a Jasco J-1500 spectropolarimeter (Jasco Inc, USA) equipped with a JascoPeltier-type temperature controller (CDF-426S/15), recording both

UV/Vis and CD spectra. CD melting experiments were performed at a fixed DNA concentration (2 μM), either with or without a fixed concentration (8 μM) of the given isomer in Tris-HCl buffer (10 mM) with 15 mM KCl (for Bom17) or 5 mM KCl (for TBA, Tel-22-K^+, *c-MYC* Pu22, *c-MYC* Pu24T, and dsDNA) and 15 mM NaCl (for Tel22-Na^+). The resulting plots enabled the calculation of melting temperature ($T_{1/2}$) values using sigmoidal nonlinear curve-fitting procedures using OriginPro 2020b.

Competitive binding studies

- were performed based on a fluorescence resonance energy transfer (FRET) melting assay. The stock solution of dual-labeled DNA (Tel22) (Table S1) with FAM (6-carboxyfluorescein) at the 5′-end and TAMRA (6-carboxytetramethylrhodamine) at the 3′-end was prepared in MilliQ water at a 100 μM concentration. The DNA was prefolded in a 10 mM sodium cacodylate buffer (pH 7.2) with 100 mM NaCl by heating for 5 minutes at 95 °C and then cooling overnight. The experiments were performed using a JASCO FP-8550 fluorimeter equipped with a Julabo CD-B5 cooling system at temperatures ranging from 10 to 90 °C at a 2.0 °C/min heating rate, using a 492 nm excitation wavelength and recording the emission spectra in the range of 495-800 nm in quartz cell cuvettes with standard path lengths of 10 mm. The sample containing 0.2 μM of labeled oligonucleotide in 40 mM NaCl and 10 mM sodium cacodylate buffer (pH 7.2) was heated in the presence (2 μM) or absence of the ligand and with or without different ratios of dsDNA to compete. The normalized emission of FAM (read at 518 nm) was plotted as a function of temperature. The $T_{1/2}$ was calculated using sigmoidal nonlinear curve-fitting procedures in OriginPro 2020b.
- were carried out using the ^{1}H NMR technique. The stock solution of *c-MYC* Pu22 (200 μM, 33 mM KCl) in Tris-HCl buffer (10 mM, 10% D_2O, pH 7.2) and **Py-Azo4F-3N** *trans*/*cis*-rich PSS (200 μM) was titrated with varying concentrations of dsDNA (0 μM, 50 μM, 100 μM, 150 μM, 200 μM), acting as a competitor. Spectra were recorded at 298 K on a Bruker 600 MHz spectrometer using excitation sculpting and 256 scans

CD spectra were recorded using a Jasco J-1500 spectropolarimeter (Jasco Inc, USA) equipped with a JascoPeltier-type temperature controller (CDF-426S/15). Prior to use, the optical chamber of the CD spectrometer was deoxygenated with nitrogen and maintained under a nitrogen atmosphere during measurements. Appropriate references were subtracted from the obtained CD spectra. CD titration experiments were conducted at a fixed DNA concentration (2 μM), with incremental addition of the *trans* or *cis*-rich mixture of **Py-Azo4F-3N** (to 5 eq) in Tris-HCl buffer (10 mM) pH 7.2 with 100 mM KCl/NaCl.

^{1}H NMR titration experiments were carried out with *c-MYC* Pu22 (33 mM KCl) and Tel22-Na^+ (70 mM NaCl) in Tris-HCl buffer (10 mM, 10% D_2O, pH = 7.2) with the final concentration of 200 μM. G4s were titrated with increasing amounts of *trans* and *cis*-rich mixture from a 20 mM DMSO-d6 stock solution. Spectra were recorded at 298 K on a Bruker 600 MHz spectrometer (*c-MYC* Pu22) or Bruker 600 MHz (Tel-22Na^+) spectrometer equipped with a 5 mm TCI cryoprobe. Excitation sculpting was used in the ^{1}H NMR experiments, and 256 scans were recorded.

Cell cultures and compound preparation were conducted as follows: HeLa cells and U2OS cells were cultured at 37°C in a 5% CO_2 atmosphere in DMEM (Dulbecco's Modified Eagle

Medium) supplemented with 10% FBS (fetal bovine serum) and 1% penicillin/streptomycin. Cells were rinsed with PBS (Phosphate-Buffered Saline) and detached from the surfaces using Trypsin-EDTA (0.25%) from Gibco®. **Py-Azo4F-3N** was dissolved in DMSO to prepare stock solutions with different concentrations (20, 10, and 5 mM) and stored at −20 °C.

Cell viability was assessed using the PrestoBlue cell viability reagent (Invitrogen, Ref. No.: A13261) following the manufacturer's instructions. Briefly, 5000 cells for HeLa and 4000 cells for U2OS were seeded per well in a complete medium on 96-well plates the day before treatment. Prior to addition to the cells, the **Py-Azo4F-3N** stock solutions, when required, were irradiated with light of ≥550 nm, and then the solutions containing various ratios of the isomers were diluted in the culture medium at the final concentration required. At 48 h post-treatment, 20µL of PrestoBlue was added to each well, and the cells were further incubated at 37 °C for 3h. Fluorescence (excitation 560 nm, emission 590 nm, 9 nm bandwidth) was measured using a Synergy H4 microplate reader (Biotek). At least three independent experiments were performed for each mixture (dark state or irradiated). Data are presented as mean ± SD.

2. SYNTHESIS AND CHARACTERIZATION

Py-Azo4F-3N was synthesized according to the route depicted in Scheme S1. Compounds: **2-6**,[1] **7**[2] were prepared according to literature procedures.

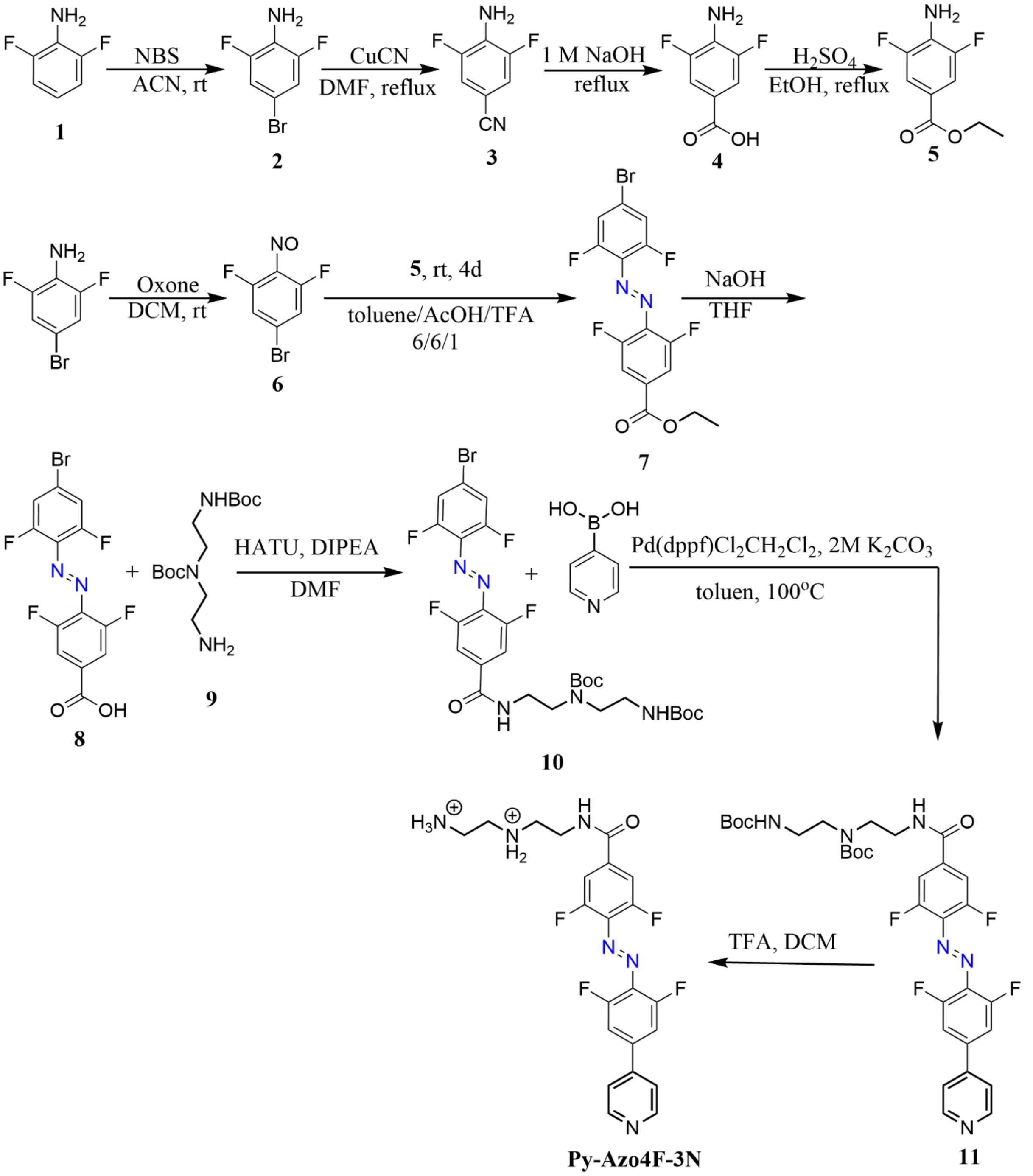

Scheme S1. Synthetic route of **Py-Azo4F-3N**.

Compound 8: A solution of **7** (360 mg, 0.89 mmol, 1 eq) and NaOH (110 mg, 2.67 mmol, 3 eq) in a mixture of H_2O/THF 2:1 (15 mL) was stirred for 4 h. The mixture was then diluted with 1M HCl (50 mL), extracted with ethyl acetate, and the organic phase was dried over Na_2SO_4, filtered, and concentrated under reduced pressure to afford **8** as a red solid (0.33 g quant.).

^{1}H NMR (400 MHz, **DMSO-d6**) δ: 7.85 – 7.75 (m, 4H).
^{19}F NMR (376 MHz, DMSO-d6) δ: -116.6 (d, *J* = 9.8 Hz), -118.1 (d, *J* = 10.0 Hz).
HRMS m/z (ESI): $C_{13}H_5BrF_4N_2O_2$ $[M+H]^+$, calculated: 376.9549, found: 376.9645.

Compound 10: A solution of **8** (100 mg, 0.27 mmol, 1.0 eq.) in DMF (10 mL) was treated with DIPEA (83 mg, 0.64 mmol, 2.4 eq.) and HATU (122 mg, 0.32 mmol, 1.2 eq.) followed by the addition of **9** (97 mg, 0.32 mmol, 1.2 eq.) after 5 minutes. The reaction mixture was stirred for 2 hours at room temperature. Upon completion, the solution was diluted with brine (30 mL), and extracted with ethyl acetate. The organic phase was dried over Na_2SO_4, filtered, and concentrated under reduced pressure. The resulting residue was purified by column chromatography (DCM/MeOH: 100/0 to 96/4) to give **10** as a red solid (122 mg, 68%).

^{1}H NMR (400 MHz, Acetonitrile-*d*$_3$) δ: 7.67 – 7.52 (m, 2H), 7.48(d, *J* = 8.7 H 2H), 5.44 (s, 1H), 3.49 (t, *J* = 5.5 Hz, 2H), 3.47 – 3.38 (m, 2H), 3.32 – 3.24 (m, 2H), 3.21 – 3.14 (m, 2H), 1.43 – 1.38 (m, 18H).
^{19}F NMR (376 MHz, Acetonitrile-*d*$_3$) δ: -120.38 (d, *J* = 9.6 Hz), -121.29 – -121.52 (m).
HRMS m/z (ESI): $C_{27}H_{32}BrF_4N_5O_5$ $[M+H]^+$, calculated: 662.1601, found: 662.1618.

Compound 11: To a solution of **10** (120 mg, 0.18 mmol, 1 eq), 4-Pyridinylboronic acid (210 mg, 0.54 mmol, 3 eq), and Pd(dppf)Cl_2·CH_2Cl_2 (15 mg, 0.018 mmol, 0.1 eq) in toluene (10 mL) was added 2M K_2CO_3 (0.27 mL, 3 eq), and the resulting solution was stirred overnight at 100 °C. After cooling, the solution was diluted with water (100 mL), and extracted with ethyl acetate. The organic phase was dried over Na_2SO_4, filtered, and concentrated under reduced pressure. The obtained residue was subjected to column chromatography (using DCM/MeOH/TEA: 99/0/1 to 97/2/1) to afford compound **11** as a red solid (62 mg, 52% yield).

^{1}H NMR (400 MHz, Acetonitrile-*d*$_3$) δ: 8.75 – 8.68 (m, 2H), 7.71 – 7.64 (m, 2H), 7.65 – 7.56 (m, 4H), 5.45 (s, 1H), 3.50 (t, *J* = 5.7 Hz, 2H), 3.48 – 3.39 (m, 2H), 3.34 – 3.25 (m, 2H), 3.22 – 3.13 (m, 2H), 1.49 – 1.37 (m, 18H).
^{19}F NMR (376 MHz, Acetonitrile-*d*$_3$) δ: -121.0 (d, *J* = 11.3 Hz, 2F), -121.29 – -121.52 (m, 2F).
HRMS m/z (ESI): $C_{32}H_{36}F_4N_6O_5$ $[M+H]^+$, calculated: 661.2772, found: 661.2762.

Py-Azo4F-3N: Compound **11** (50 mg, 0.076 mmol) was dissolved in DCM (5 mL), followed by the addition of TFA (300 µl), and the resulting solution was stirred overnight. Subsequently, the solvent was evaporated under reduced pressure and the compound was purified by HPLC using a gradient from water to water/MeCN (50/50) over15 minutes, yielding the TFA salt of **Py-Azo4F-3N** as a red solid (54 mg, 90% yield).

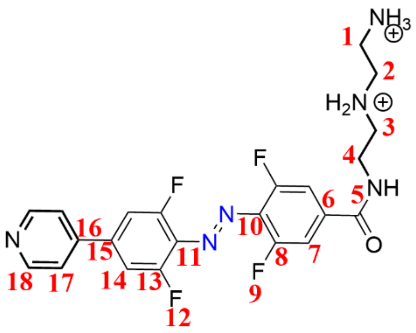

^{1}H NMR (601 MHz, D$_2$O) δ: 8.88 (d, J = 6.9 Hz, 2H$_{18}$), 8.36 (d, J = 6.9 Hz, 2H$_{17}$), 7.81 (d, J = 9.7 Hz, 2H$_{14}$), 7.66 (d, J = 9.3 Hz, 2H$_7$), 3.81 (t, J = 5.7 Hz, 2H$_4$), 3.54 – 3.48 (m, 2H$_2$), 3.47 – 3.41 (m, 4H$_{1,3}$).

^{19}F NMR (376 MHz, D$_2$O) δ: -74.1 (s, 9F, TFA), -119.5 (d, J = 11.5 Hz, 2F$_{12}$), -120.2 (d, J = 10.8 Hz, 2F$_9$).

^{13}C NMR (151 MHz, D$_2$O) δ: 167.8 (C$_5$), 156.3 (dd, J = 260.9, 4.2 Hz, 2C$_8$), 155.7 (dd, J = 261.2, 3.3 Hz, 2C$_{13}$), 154.0 (C$_{16}$),142.1 (2C$_{18}$), 139.2 (t, J = 10.5 Hz, C$_{15}$), 137.1 (t, J = 9.4, Hz, C$_6$), 132.7 (t, J = 10.3 Hz, C$_{10}$), 131.8 (t, J = 10.1 Hz, C$_{11}$), 124.8 (2C$_{17}$), 112.8 (dd, J = 22.5, J = 3.1 Hz, 2C$_{14}$), 112.2 (dd, J = 22.2, 3.0 Hz, 2C$_7$), 47.9 (C$_3$), 44.4 (C$_2$), 36.5 (C$_4$), 35.4 (C$_1$).

HRMS m/z (ESI): $C_{22}H_{20}F_4N_6O$ [M+H]$^+$, calculated: 461.1713, found: 461.1699.

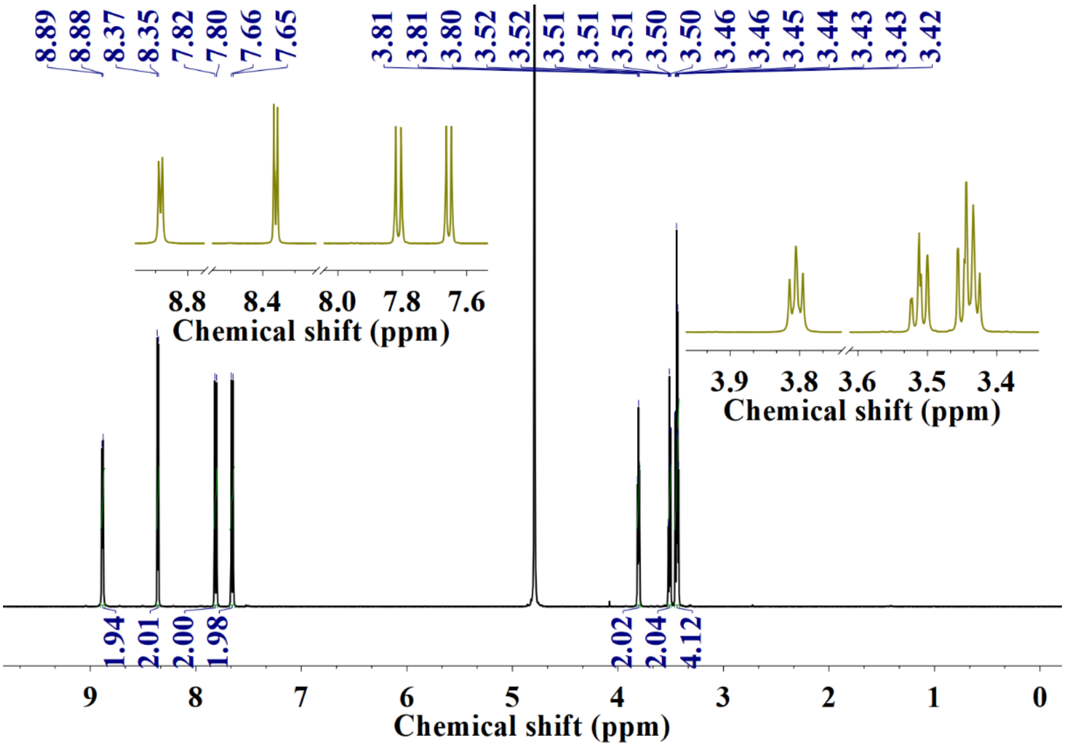

Figure S1. ^{1}H NMR (600 MHz, D$_2$O, 298 K) spectrum of **Py-Azo4F-3N**.

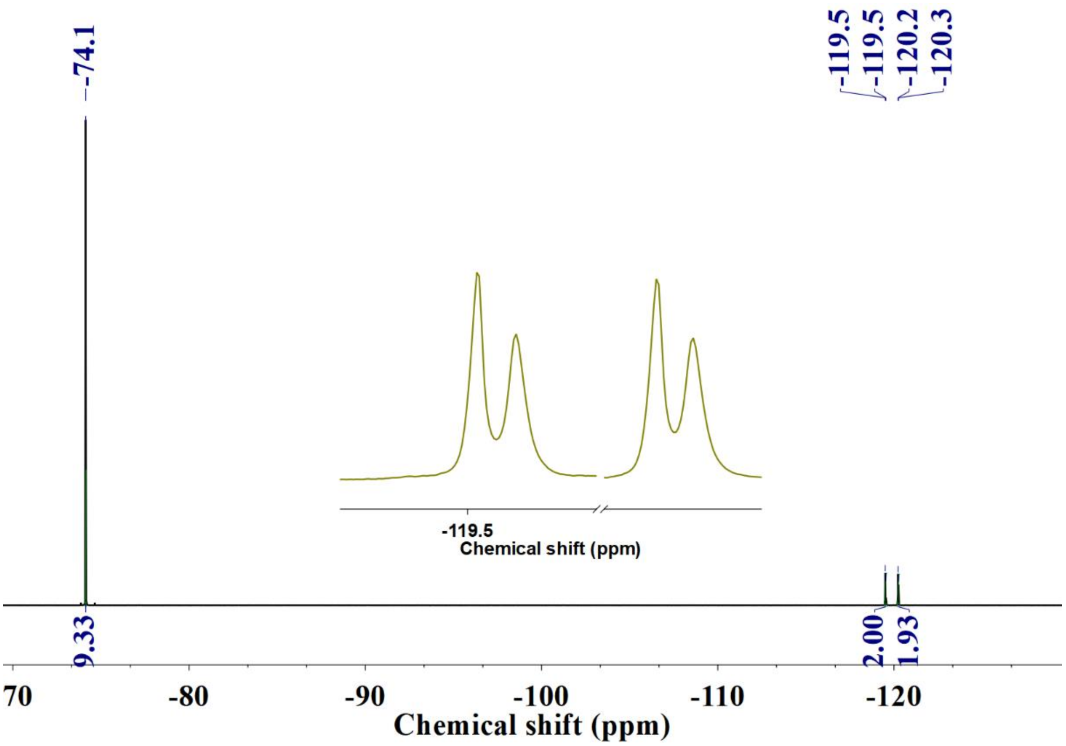

Figure S2. ^{19}F NMR (376 MHz, D_2O, 298 K) spectrum of **Py-Azo4F-3N**. The signal at 74.1 ppm corresponds to TFA salt.

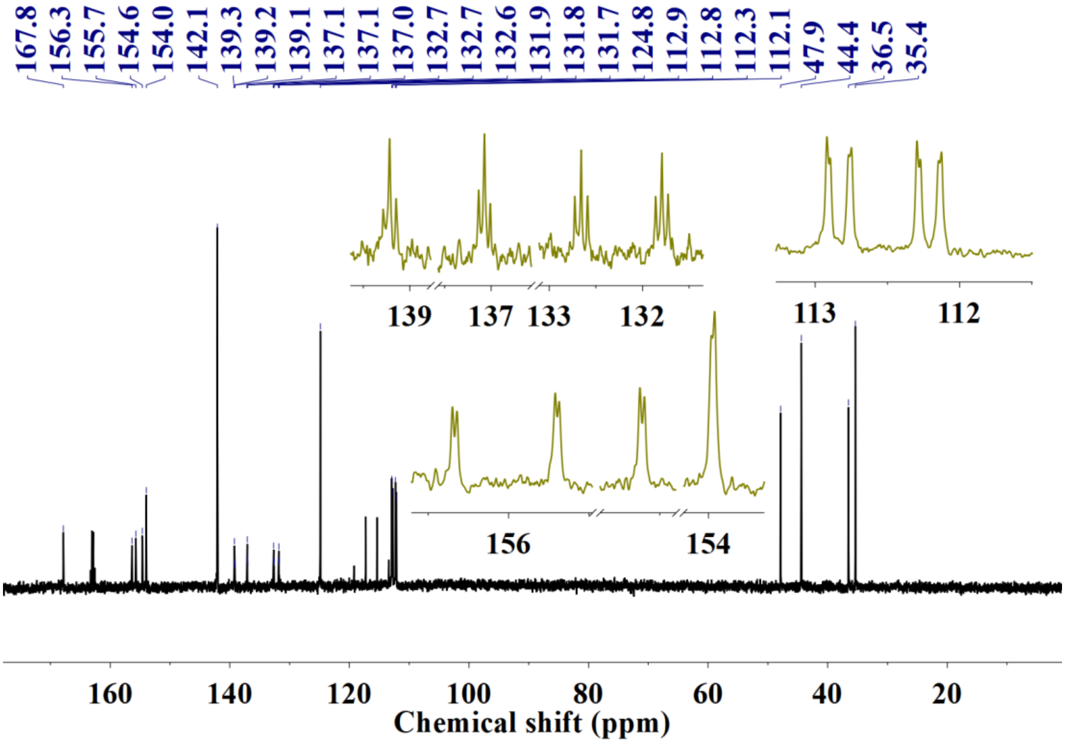

Figure S3. ^{13}C NMR (151 MHz, D_2O, 298 K) spectrum of **Py-Azo4F-3N**.

3. PHOTOISOMERIZATION STUDIES

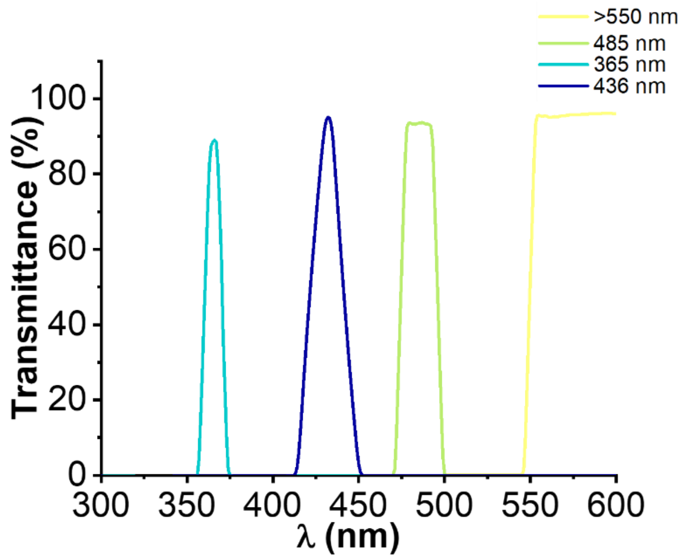

Figure S4. Transmission spectra of the used filters.

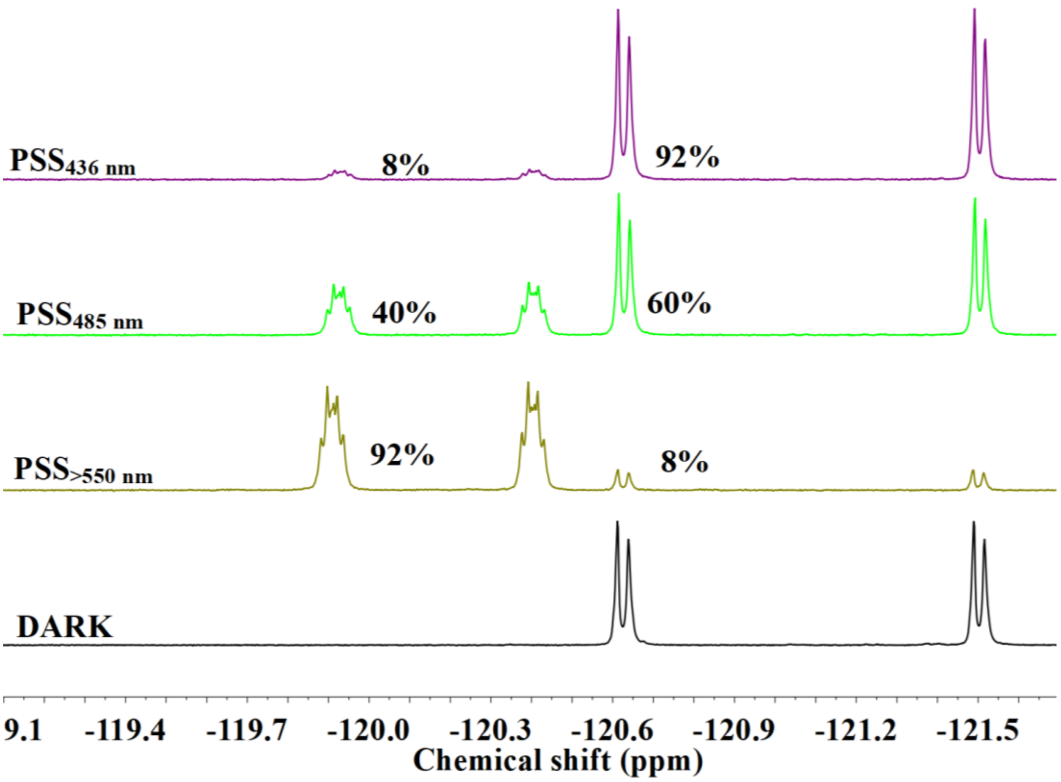

Figure S5. The PSSs of **Py-Azo4F-3N** ($C_{Py\text{-}Azo4F\text{-}3N\text{-}C}$ = 3.3 mM, in methanol-d4 at 25 °C) were determined using ^{19}F NMR spectroscopy. The proportions of the *trans* and *cis* isomers in the solution were estimated based on the intensity ratios of the integrals of the corresponding peaks.

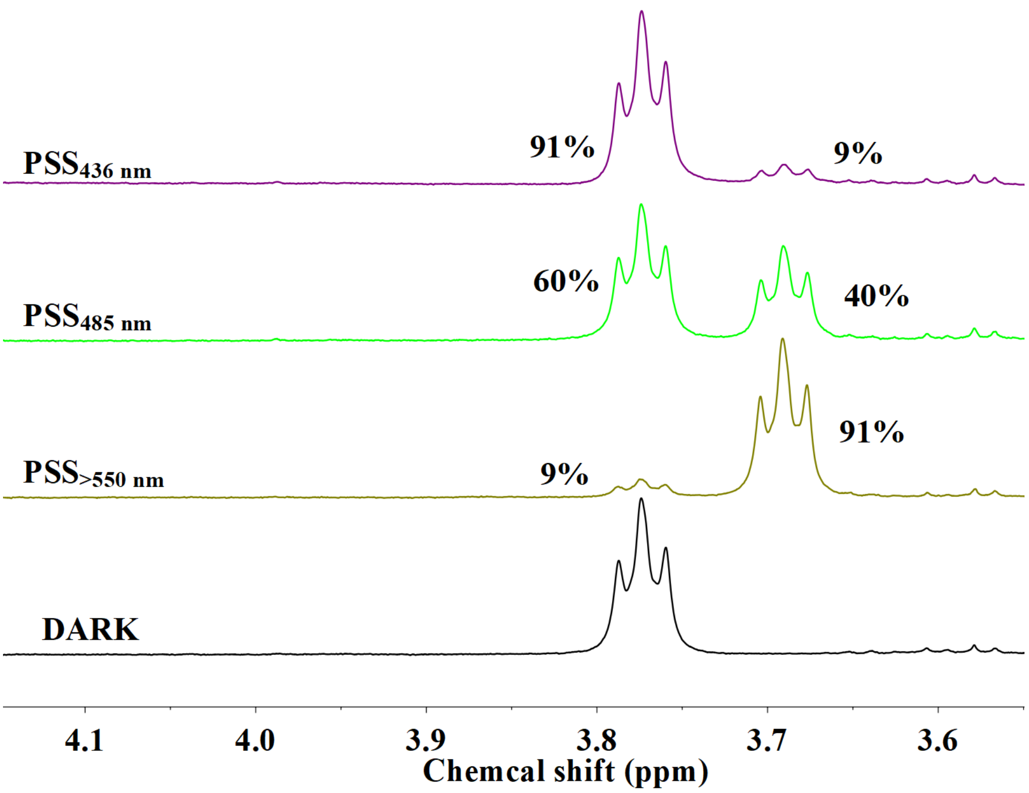

Figure S6. The PSSs of **Py-Azo4F-3N** ($C_{Py\text{-}Azo4F\text{-}3N\text{-}C}$ = 3.3 mM, in methanol-d4 at 25 °C) were determined using ^{1}H NMR spectroscopy. The proportions of the *trans* and *cis* isomers in the solution were estimated based on the intensity ratios of the integrals of the corresponding peaks.

4. THERMAL STABILITY

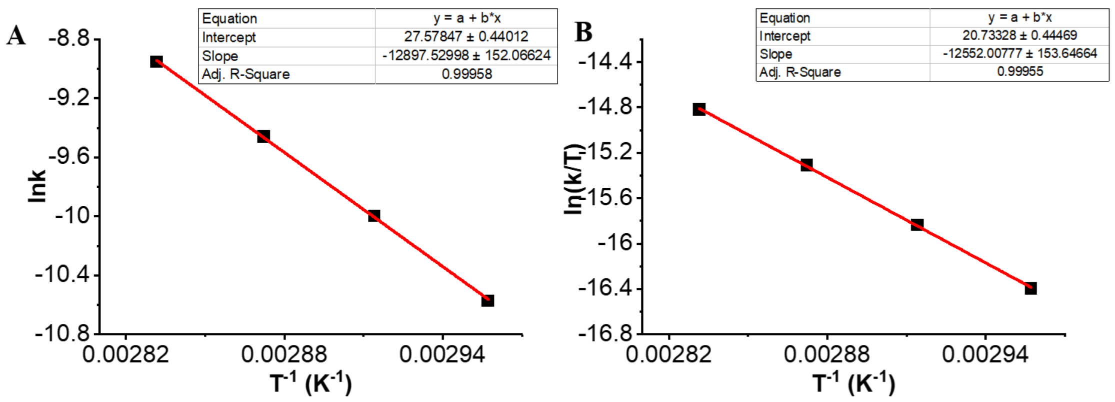

Figure S7. (A) Arrhenius plot for **Py-Azo4F-3N**, the rate constants of the *cis-trans* isomerization process at different temperatures were determined by the exponential curve fitting of the plot: absorbance vs. time. The slope is equal to -Ea/R. (B) Eyring plot for **Py-Azo4F-3N**, slope is equal to -ΔH‡/R, intercept: ln((κk$_B$)/h) + ΔS‡/R.

Table S1. Isomerization rates $k_{cis \rightarrow trans}$ (at 298 K), thermal half-life ($\tau_{1/2}$) (at 298 K), Arrhenius prefactor A, activation energies E$_a$, as well as Eyring activation free energies ΔG‡, enthalpies ΔH‡ and entropies ΔS‡ for **Py-Azo4F-3N** in DMSO.

Cmpd	$k^{[a]}_{E\rightarrow Z}$ [s^{-1}]	$lnk_{E\rightarrow Z}$	$\tau^{[b]}_{1/2}$ [h]	A[c] [s^{-1}]	Ea $[\frac{kJ}{mol}]$	ΔH‡ $[\frac{kJ}{mol}]$	ΔS‡ $[\frac{J}{mol \times K}]$	ΔG‡ $[\frac{kJ}{mol}]$
**Py-Azo4F-3N**	1.55 ± 0.78	-15.7 ± 0.7	1242 ± 625	9.50 ± 3.38	107.2 ± 1.3	104.4 ± 1.3	-25.2 ± 3.7	111.9 ± 1.7

[a] 10^{-7} at 298 K and [b] at 298 K, estimated from the Arrhenius equation. [c] 10^{11}

5. DNA STUDIES

5.1 DNA CHARACTERIZATION

Synthetic oligonucleotides were purchased from MERCK. The sequences used are listed in Table S2. All the oligonucleotides (except those for the NMR and competitive binding experiments) were prefolded in 10 mM TRIS buffer (pH 7.2), with 100 mM KCl/NaCl by heating for 5 min at 95 °C and then cooling overnight.

Table S2. Oligonucleotides used in this study.

Name	Sequence [5' – 3']	Length (bp)	Topology and description
**Tel22-Na$^+$**	AGGGTTAGGGTTAGGGTTAGGG	22	antiparallel
**F-Tel22-Na$^+$-T**	FAM[a]-AGGGTTAGGGTTAGGGTTAGGG-TAMRA[a]	22	antiparallel
**Bom17**	GGTTAGGTTAGGTTAGG	17	antiparallel
**TBA**	GGTTGGTGTGGTTGG	15	antiparallel
**Tel22-K$^+$**	AGGGTTAGGGTTAGGGTTAGGG	22	hybrid
**VEGF**	GGGAGGGTTGGGGTGGG	17	parallel
***c-MYC* Pu22**	TGAGGGTGGGTAGGGTGGGTAA	22	parallel
***c-MYC* Pu24-T**	TGAGGGTGGTGAGGGTGGGGAAGG	24	parallel
HIF-1α	GCGCGGGGAGGGGAGAGGGGGCGGGAGCGCG	31	parallel
**Cy5-*HIF-1α***	Cy5[b]-GCGCGGGGAGGGGAGAGGGGGCGGGAGCGCG	31	parallel
**dsDNA**	CAATCGGATCGAATTCGATCCGATTG	26	double stranded

[a] FAM - 6-carboxyfluorescein; TAMRA - 6-carboxy-tetramethylrhodamine [b] Cy5 - Cyanine5

5.2 MELTING STUDIES

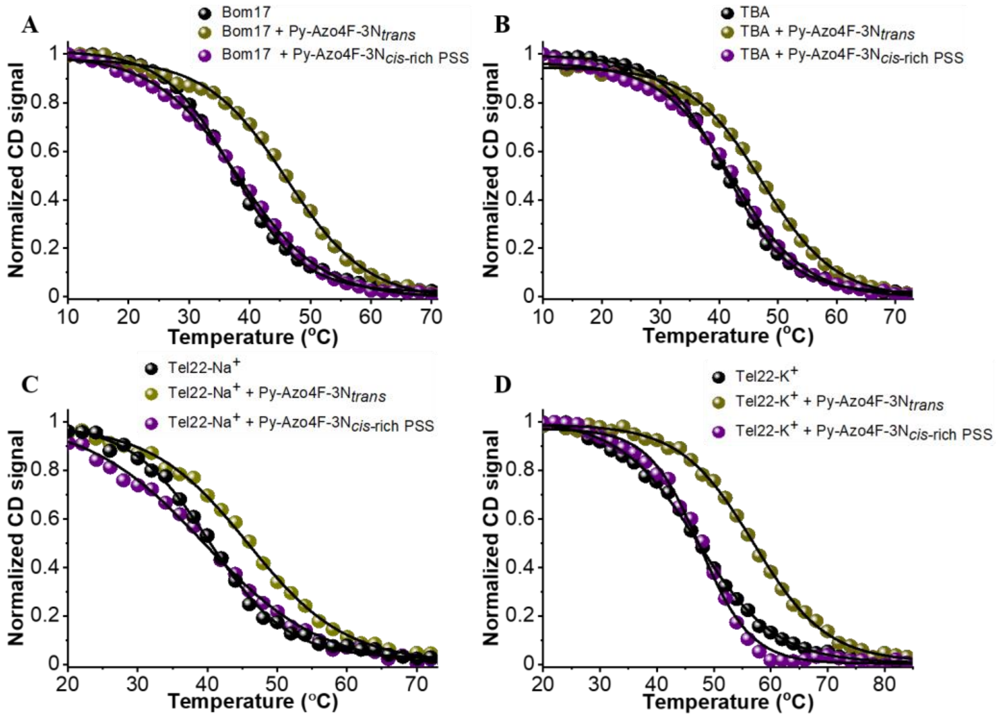

Figure S8. Melting curves for Bom17 (A), TBA (B), Tel22-Na$^+$ (C) and Tel22-K$^+$(D) in the absence and presence of *trans/cis-rich PSS* of **Py-Azo4F-3N** ($C_{\mathbf{Py\text{-}Azo4F\text{-}3N}}$ = 8.0 µM, C_{G4s} = 2.0 µM, C_{KCl} = 5 mM (TBA, Tel22-K$^+$) or 15 mM (Bom17), C_{NaCl} = 15 mM, C_{Tris} = 10 mM pH 7.2).

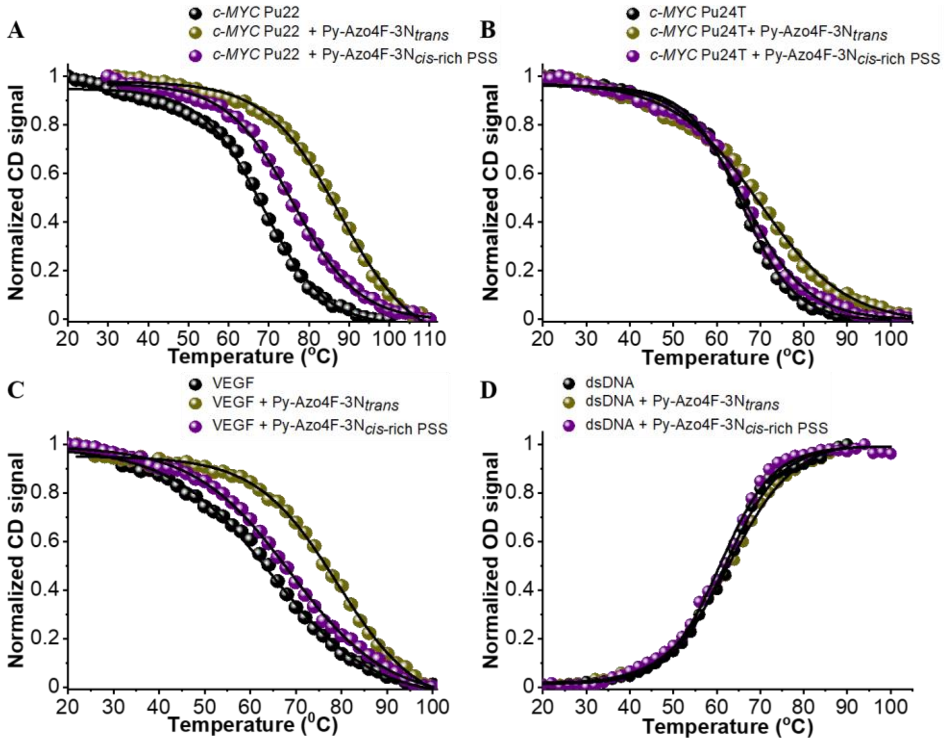

Figure S9. Melting curves for *c-MYC* Pu22 (A), *c-MYC* Pu24 (B), VEGF (C), and dsDNA (D) in the absence and presence of *trans/cis-rich PSS* of **Py-Azo4F-3N** ($C_{\mathbf{Py\text{-}Azo4F\text{-}3N}}$ = 8.0 µM, $C_{G4s/dsDNA}$ = 2.0 µM, C_{KCl} = 5 mM, C_{Tris} = 10 mM pH 7.2).

Table S3. Temperature changes induced by **Py-Azo4F-3N** to G4 and duplex templates.

**Oligonucleotide**	**Py-Azo4F-3N**$_{trans}$	**Py-Azo4F-3N**$_{PSS>550\ nm}$	
	ΔT_m[a]	ΔT_m[a]	δT_m[b]
**Bom17**	8.8	0.9	7.9
**TBA**	6.3	1.1	5.2
**Tel22-Na$^+$**	5.8	-0.6	6.4
**Tel22-K$^+$**	**9.9**	0.4	**9.5**
***c-MYC* Pu22**	**19.2**	7.4	**11.8**
***c-MYC* Pu24**	4.2	0.9	3.3
**VEGF**	**14.7**	4.4	**10.3**
**dsDNA**	1.0	-0.2	1.2

[a]$\Delta = T_m^{trans/cis} - T_m^{DNA}$ [b] $\delta T_m = T_m^{trans} - T_m^{cis}$

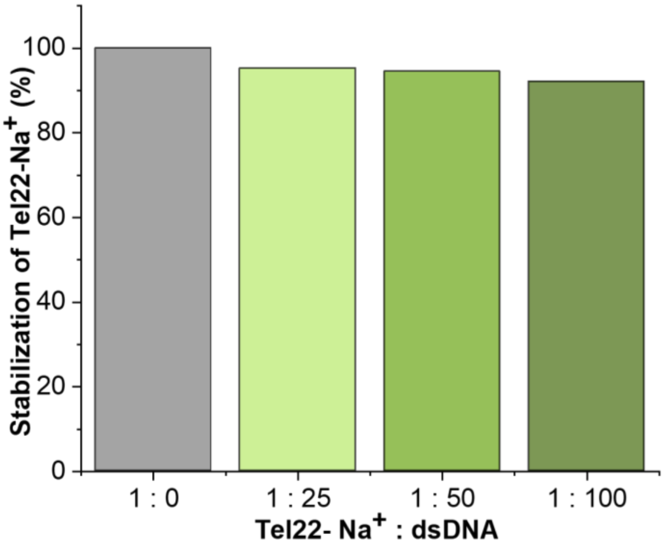

Figure S10. Retained thermal stabilization of F-Tel22-Na$^+$-T (0.2 μM) by 2 μM of **Py-Azo4F-3N**$_{trans}$ in the presence of increasing concentrations of dsDNA (0-20 μM). Experimental conditions: 40 mM NaCl, 10 mM sodium cacodylate buffer, pH = 7.2.

5.3 G4 DNA CONFORMATIONAL CHANGES

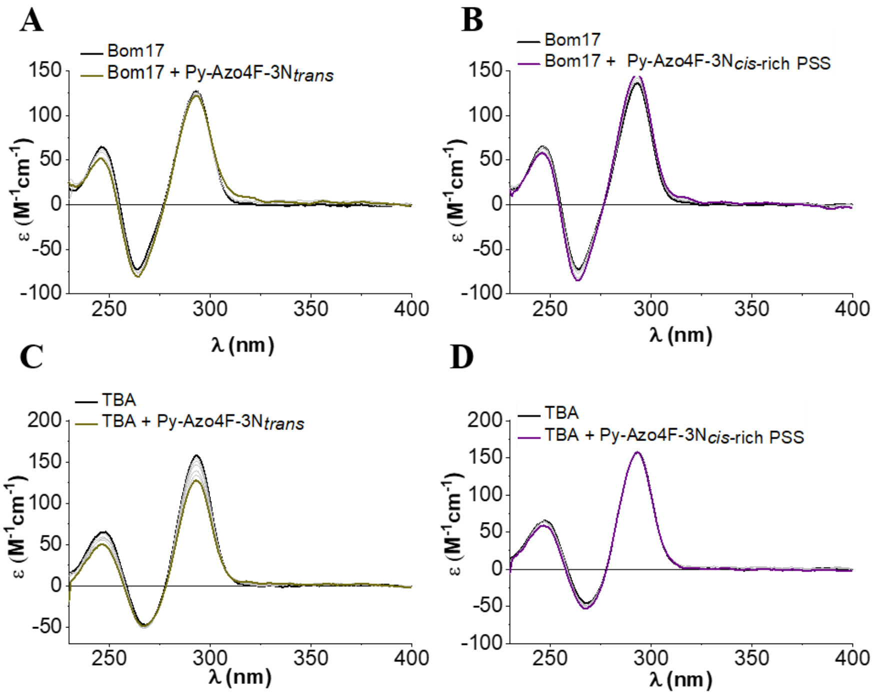

Figure S11. CD spectra of Bom17 (A, B) and TBA (C, D) upon addition of *trans* (A, C) and *cis*-rich mixture (B, D) of **Py-Azo4F-3N**. Black and olive/violet lines correspond to the spectra at 0 eq. and 5 eq. of ligand, respectively. Experimental conditions: C_{G4} = 2.0 µM, Tris-HCl 50.0 mM, pH 7.2, KCl 100 mM.

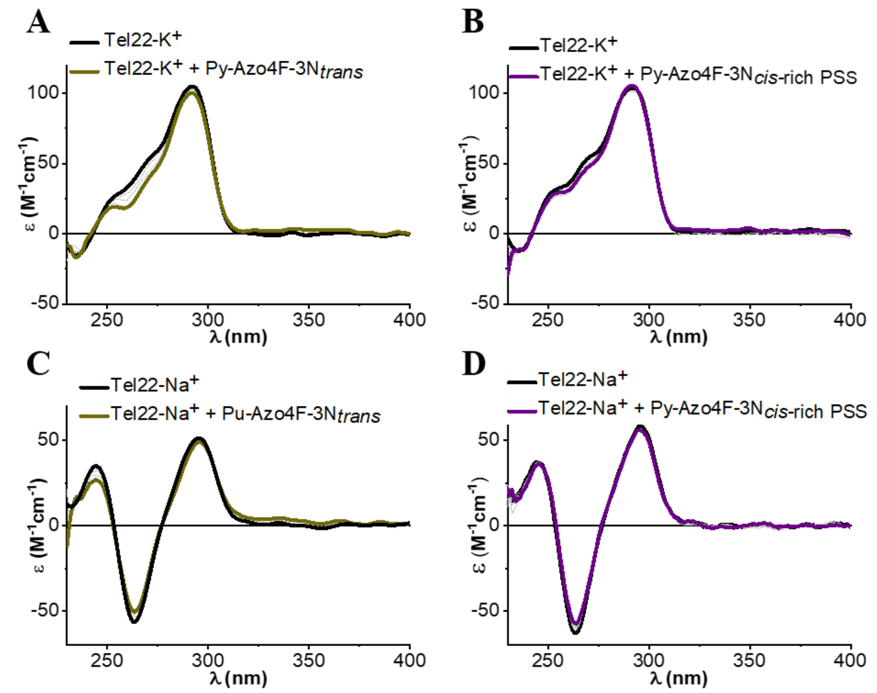

Figure S12. CD spectra of Tel22-K$^+$ (A, B) and Tel22-Na$^+$ (C, D) upon addition of *trans* (A, C) and *cis*-rich mixture (B, D) of **Py-Azo4F-3N**. Black and olive/violet lines correspond to the spectra at 0 eq. and 5 eq. of ligand, respectively. Experimental conditions: C_{G4} = 2.0 µM, Tris-HCl 50.0 mM, pH 7.2, NaCl/KCl 100 mM.

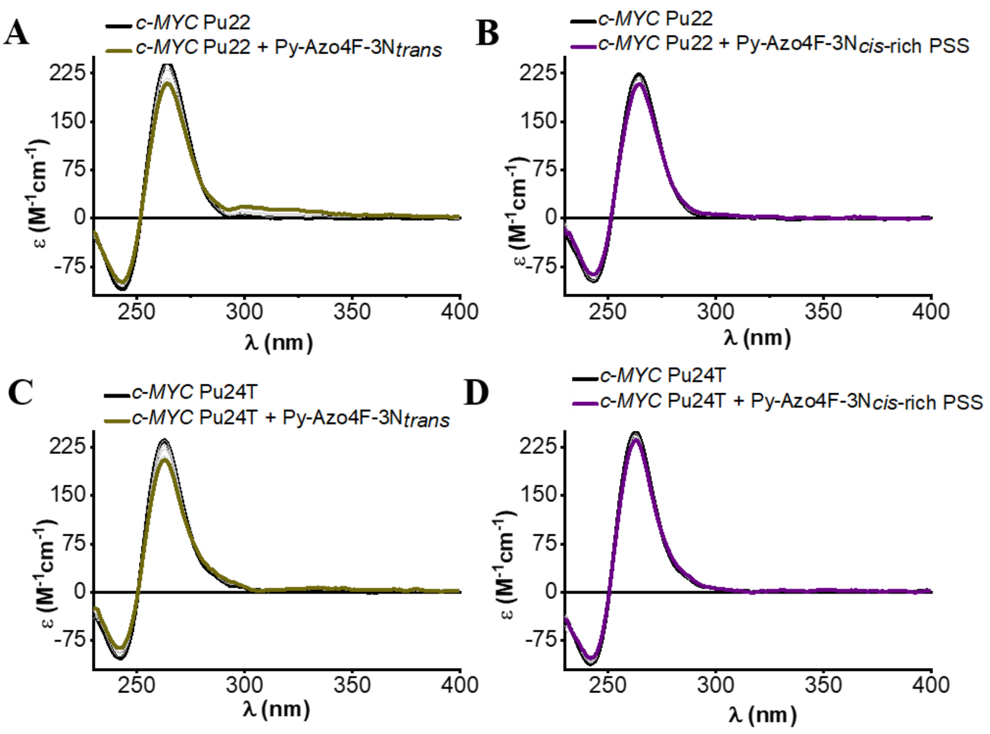

Figure S13. CD spectra of *c-MYC* Pu22 (A, B) and *c-MYC* Pu24 (C, D) upon addition of *trans* (A, C) and *cis*-rich mixture (B, D) of **Py-Azo4F-3N**. Black and olive/violet lines correspond to the spectra at 0 eq. and 5 eq. of ligand, respectively. Experimental conditions: C_{G4} = 2.0 µM, Tris-HCl 50.0 mM, pH 7.2, KCl 100 mM.

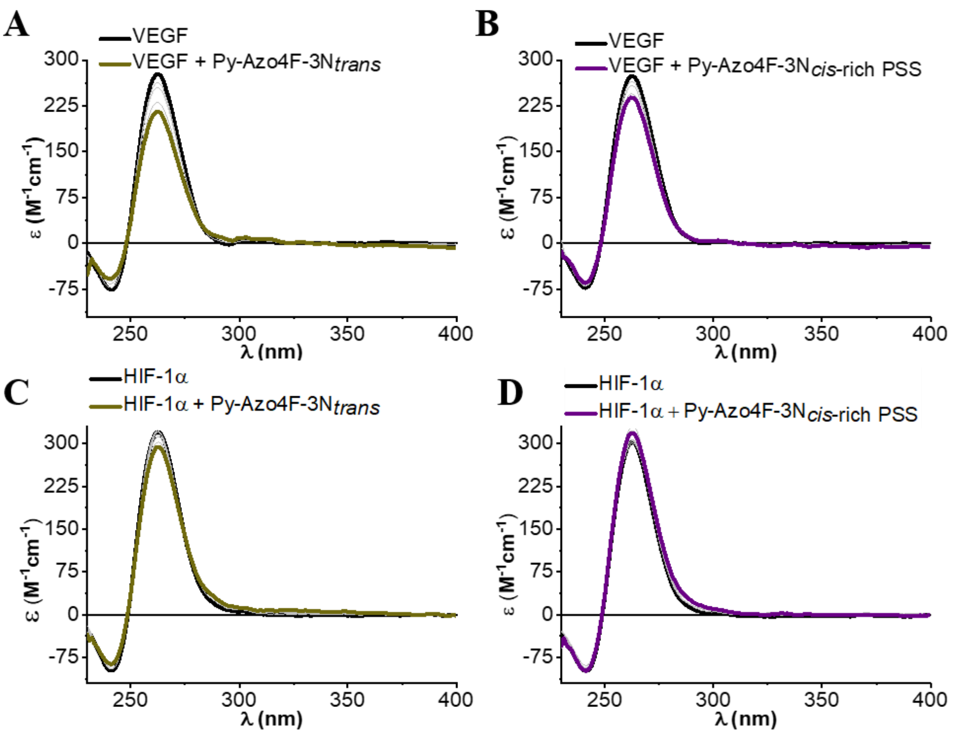

Figure S14. CD spectra of VEGF (A, B) and *HIF-1α* (C, D) upon addition of *trans* (A, C) and *cis*-rich mixture (B, D) of **Py-Azo4F-3N**. Black and olive/violet lines correspond to the spectra at 0 eq. and 5 eq. of ligand, respectively. Experimental conditions: C_{G4} = 2.0 µM, Tris-HCl 50.0 mM, pH 7.2, NaCl/KCl 100 mM.

5.4 NMR STUDIES

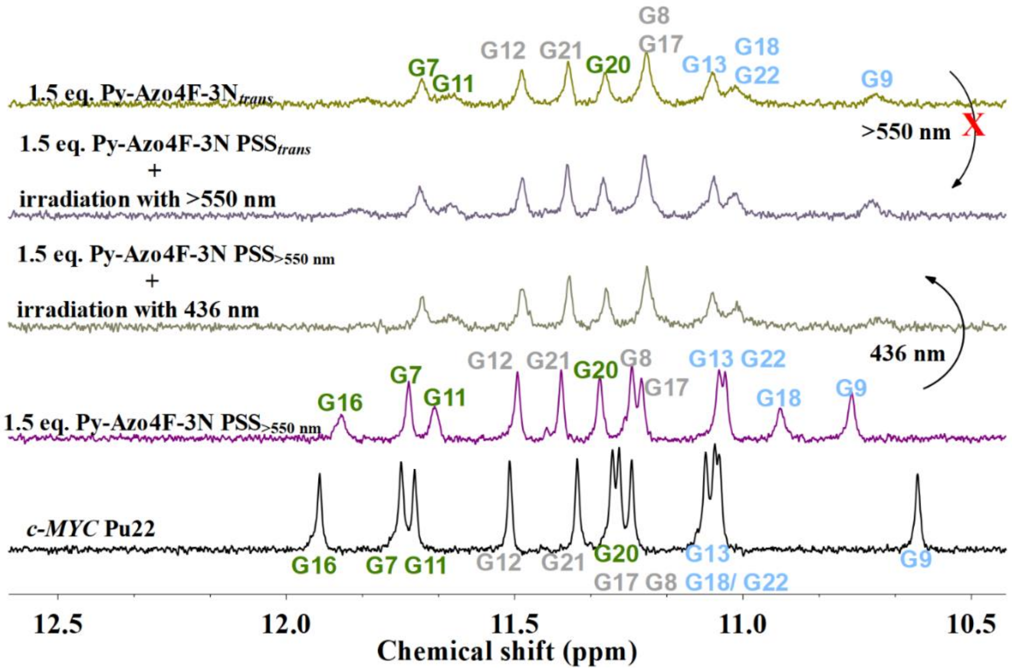

Figure S15. ^{1}H NMR spectra of *c-MYC* Pu22 (black) with 1.5 eq of **Py-Azo4F-3N**$_{trans}$ (olive) and **Py-Azo4F-3N**$_{cis\text{-rich PSS}}$ (violet). The middle ^{1}H NMR spectra were taken after irradiation with i) 436 nm (*cis*-to-*trans* isomerization, pale olive), and clearly shows the change in imino protons shift, or after irradiation with ii) ≥ 500 nm (*trans*-to-*cis* isomerization, light grey), and shows negligible change in imino protons shift and intensity.

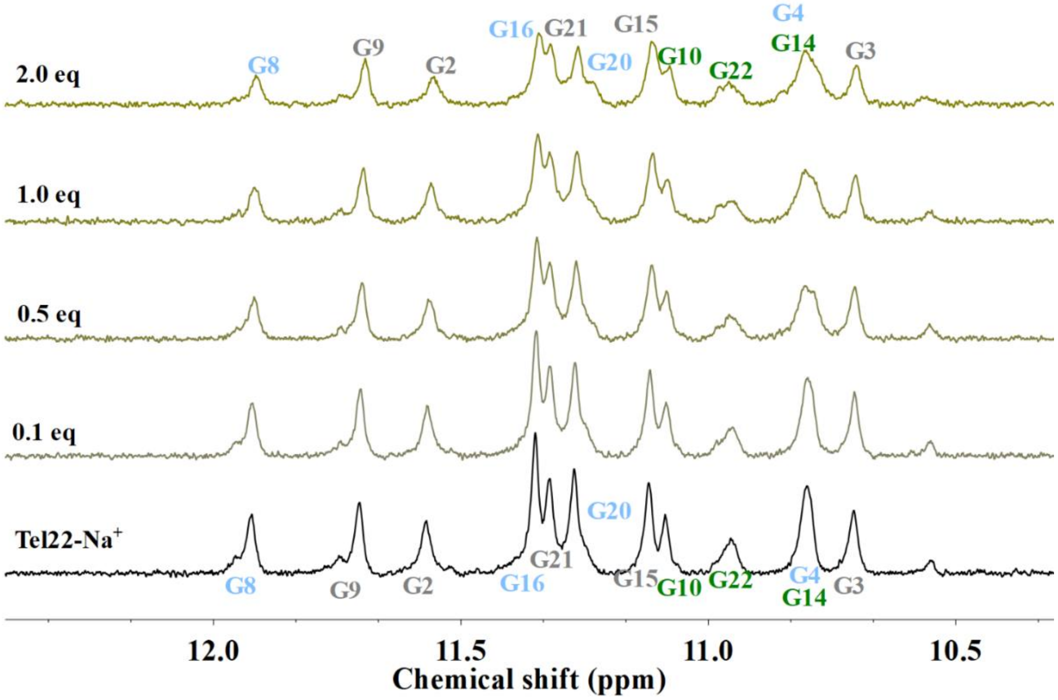

Figure S16. ^{1}H NMR spectra of the G-tetrad imino protons of Tel22-Na$^+$ in the absence (black line, bottom) and presence (olive line, top) of 2.0 eq. of **Py-Azo4F-3N**$_{trans}$. The imino protons from the 5'end are colored in green, the middle G-tetrad in grey, and the 3'end in blue.

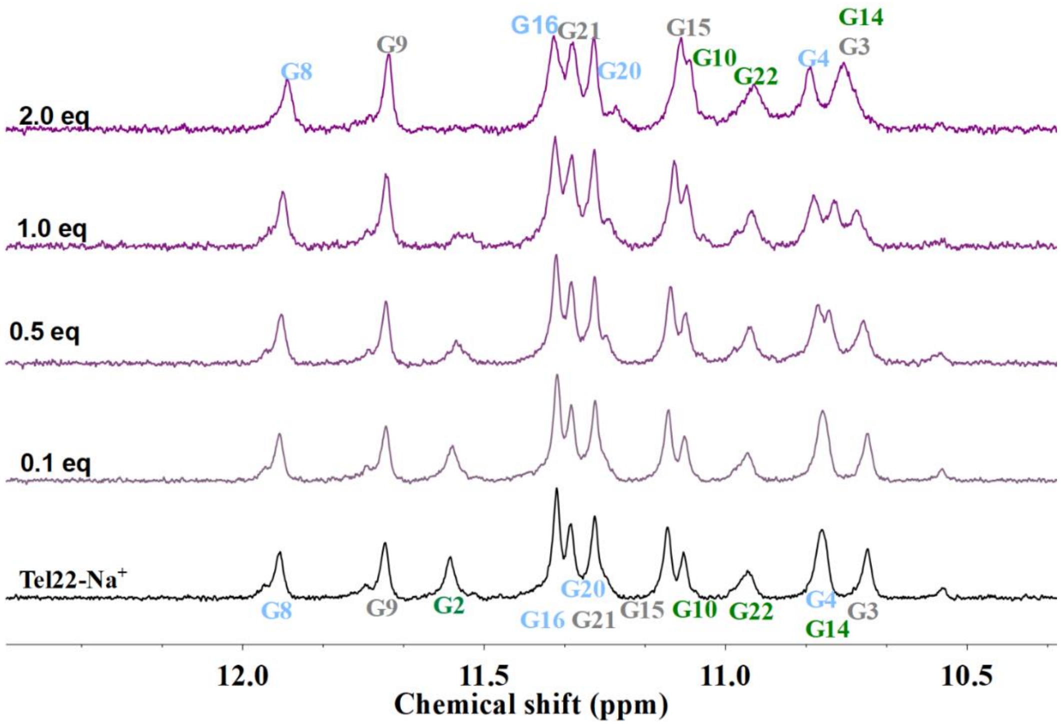

Figure S17. ^{1}H NMR spectra of the G-tetrad imino protons of Tel22-Na$^+$ in the absence (black line, bottom) and presence (violet line, top) of 2.0 eq. of **Py-Azo4F-3N**$_{cis\text{-rich PSS}}$. The imino protons from the 5'end are colored in green, the middle G-tetrad in grey, and the 3' end in blue.

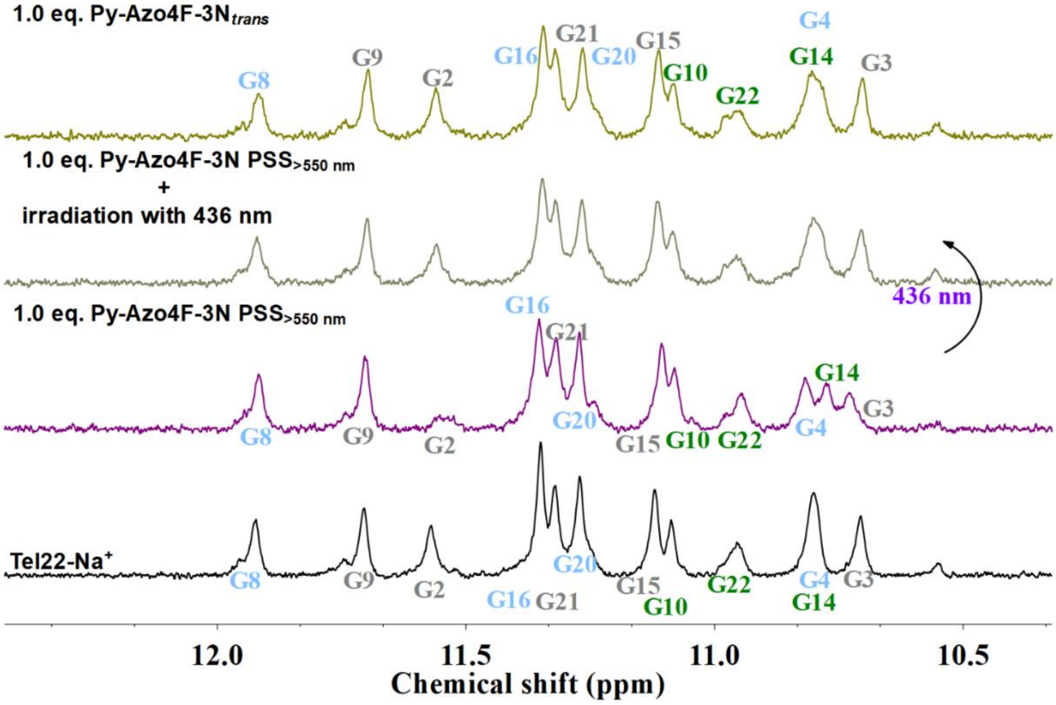

Figure S18. ^{1}H NMR spectra of Tel22-Na$^+$ (black) with of **Py-Azo4F-3N**$_{cis\text{-rich PSS}}$ (violet) and of **Py-Azo4F-3N**$_{trans}$ (olive, top). The middle ^{1}H NMR spectrum was taken after irradiation with 436 nm (*cis*-to-*trans* isomerization) and clearly shows the change in imino protons shift (pale olive).

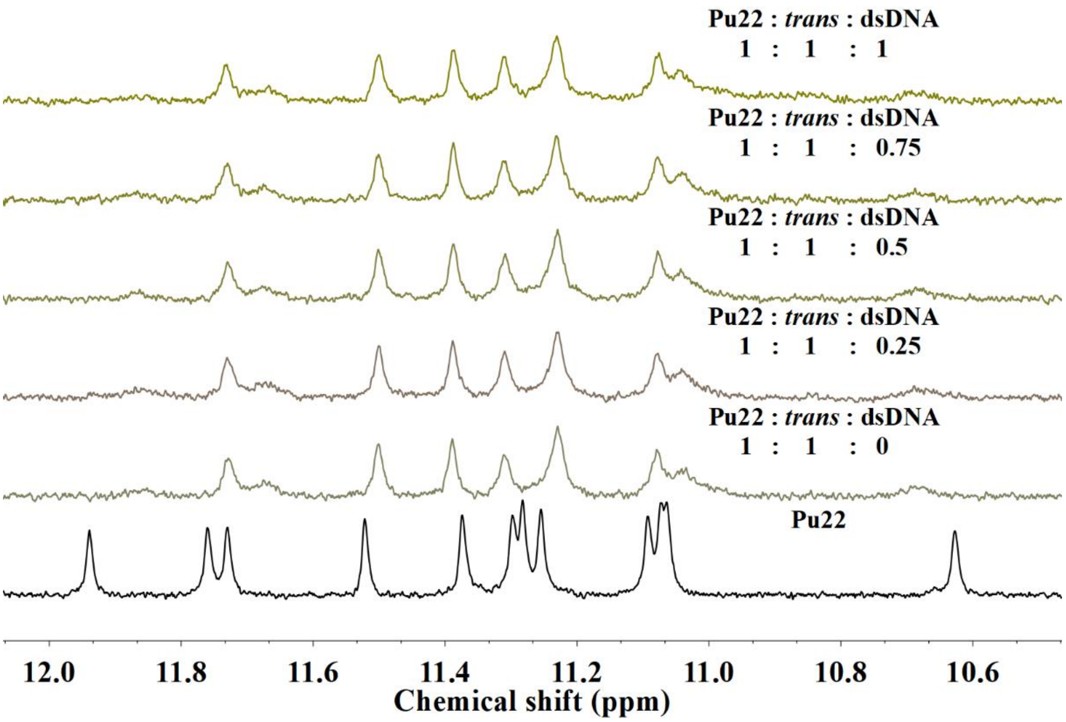

Figure S19. ^{1}H NMR spectra showing the G-tetrad imino protons of *c-MYC* Pu22 (200 μM, black line, bottom) interacting with **Py-Azo4F-3N**$_{trans}$ (200 μM olive) with increasing ratios of dsDNA (from 0 μM to 200 μM).

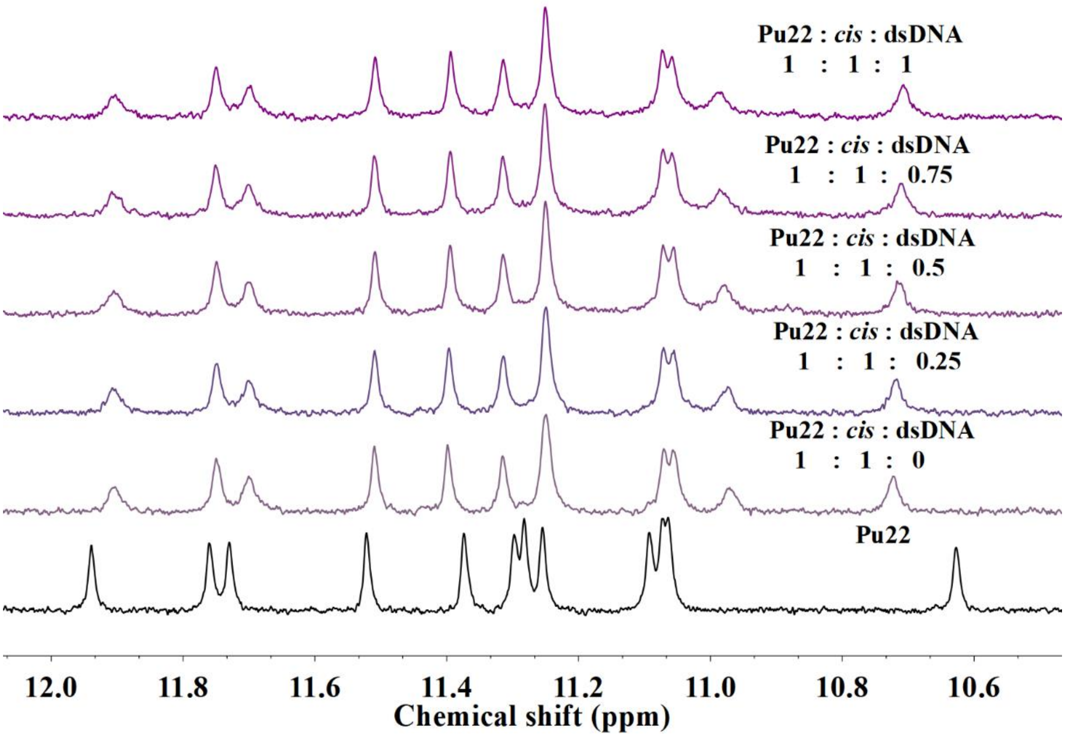

Figure S20. ^{1}H NMR spectra showing the G-tetrad imino protons of *c-MYC* Pu22 (200 μM, black line, bottom) interacting with **Py-Azo4F-3N**$_{cis\text{-rich PSS}}$ (200 μM, violet) with increasing ratios of dsDNA (from 0 μM to 200 μM).

6. THEORETICAL CALCULATIONS

To find the binding poses of *trans* and *cis* **Py-Azo4F-3N** in *c-MYC* Pu22 and Tel22-Na^+, docking calculations were performed to identify potential binding pockets. The *c-MYC* Pu22 and Tel22-Na^+ structures for the docking calculations were taken from the Protein Data Bank (PDB),[3] PDB IDs 1XAV[4] and 143D,[5] respectively, or from a representative structure of a previous Umbrella Sampling Molecular Dynamics (US MD) simulation. The docking calculations provided binding modes for *trans* and *cis* **Py-Azo4F-3N**, for which additional MD simulations were performed to study their stability and to compute the binding free energies of each isomer in each pocket. In the following, the computational details of all these steps will be explained.

6.1 UMBRELLA SAMPLING

To generate the initial geometry, the G4 structures 1XAV and 143D were taken without the coordinated K^+ and Na^+ ions and were surrounded by water in a periodic truncated octahedral box, using the *tleap* module included in AmberTools20.[6] The water molecules were described by the TIP3P[7] force field and were present within 12 Å from any G4 atom. Moreover, K^+ (*c-MYC* Pu22) or Na^+ (Tel22-Na^+) ions were added to neutralize the systems and the nucleic acid interactions were described with the OL15 force field.[8] The resulting systems were minimized with the steepest descent (10 steps) and the conjugate gradient (4990 steps) algorithms. Then, they were heated to 300 K during 1 ns in the NVT ensemble. Next, the systems were equilibrated for 0.1 ns in the NVT ensemble and for 300 ns in the NPT ensemble. The Lennard-Jones and real-space Ewald cutoffs were set to 12 Å, the bonds involving H atoms were constrained with SHAKE, the time-step was 2 fs and the Langevin thermostat and the Berendsen barostat were employed. All these calculations were performed using the Amber20 software.[6]

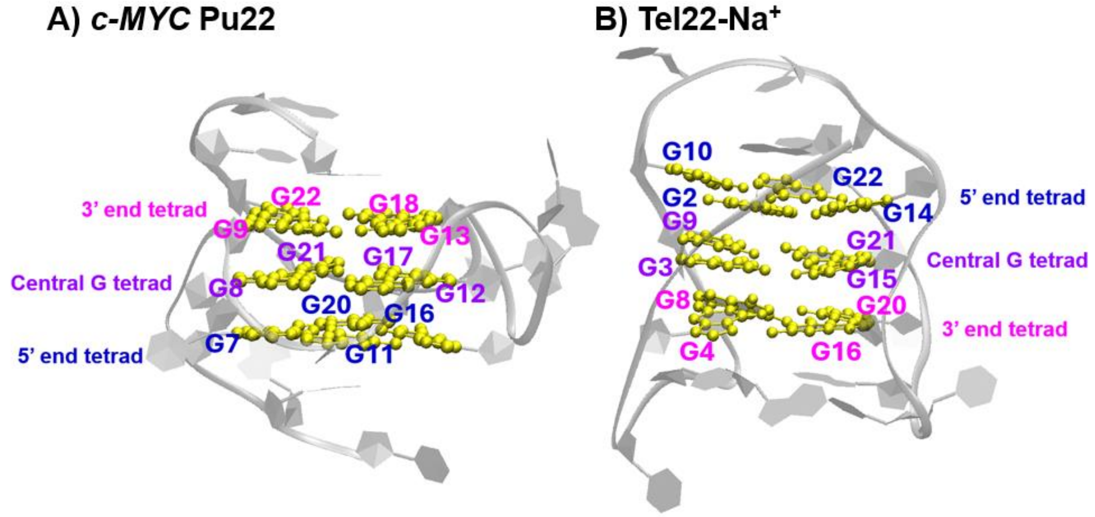

Figure S21. *c-MYC* Pu22 1XAV (A) and Tel22-Na^+ 143D (B) structures and G numbering. Gs corresponding to the 3'-end, central, and 5'-end tetrads are represented in magenta, purple and blue, respectively.

To investigate the ligand intercalation in the *c-MYC* Pu22 docking calculations, we opened the G4 structure by separating the G8-G12-G17-G21 (central G-tetrad) and G9-G13-G18-G22 (3'-end tetrad) tetrads, as represented in Figure S22A. Similarly, for Tel22-Na^+, we separated the G3-G9-G15-G21 (central G-tetrad) and G4-G8-G16-G20 (3'-end tetrad) tetrads (Figure S21B).

In both cases, we defined 5 collective variables (CV) to bias the potential energy of the system. The first CV was the distance between the centers of mass (CM) of the four central nucleobases and the four end nucleobases; the 4 remaining CVs were the distances between the CM of G8 and G9 (CV2), G12 and G13 (CV3), G17 and G18 (CV4), and G21 and G22 (CV5) for *c-MYC* Pu22; and the distances between the CM of G3 and G4 (CV2), G9 and G8 (CV3), G15 and G16 (CV4), and G21 and G20 (CV5) for Tel22-Na^+. These distances were increased from ~3.5 Å to ~7.50 Å, forcing the opening of the two tetrads. To do this, 10 ns MD simulations per window were run in Amber20 constraining the CV distances in a 50 $kcal\cdot mol^{-1}Å^{-2}$ harmonic potential. A total of 21 windows were run to cover the 3.5-7.5 Å range, centering the restraining harmonic potentials every 0.2 Å. The initial velocities of the first window were generated according to a Boltzmann distribution at 300 K, and the initial geometry corresponded to the last structure from the previous equilibration trajectory. The remaining windows started from the velocities and coordinates of the previous window. A final 200 ns NPT equilibration with the same restrictions as in the last window was performed to obtain a representative structure of the open G4 by means of the dbscan clustering of the last 100 ns, implemented in cpptraj.[9] In all windows and in the final equilibration, the rest of the MD parameters were equivalent to the ones specified in the initial equilibration. Moreover, to preserve the G4 structure in all windows, the distances between the guanines of the same tetrad were restrained to their initial values in a 50 $kcal\cdot mol^{-1}Å^{-2}$ harmonic potential. These restrictions were not enough to preserve the Tel22-Na^+ structure. Hence, we also restrained the distances between the CM of the four central nucleobases and the four 5'-end nucleobases, and between the CM of the individual nucleobases G3-G2, G9-G10, G15-G14, and G21-G22 to their initial value; and the angles of the CM of the four 3'-end nucleobases, the four central nucleobases, and the four 5'-end nucleobases, and the CM of the individual nucleobases G4-G3-G2, G8-G9-G10, G16-G15-G14, and G20-G21-G22 to 180º. Figure S23 shows the resulting opened representative structures and the root mean square displacement (RMSD) along the simulation of the last window and the equilibration.

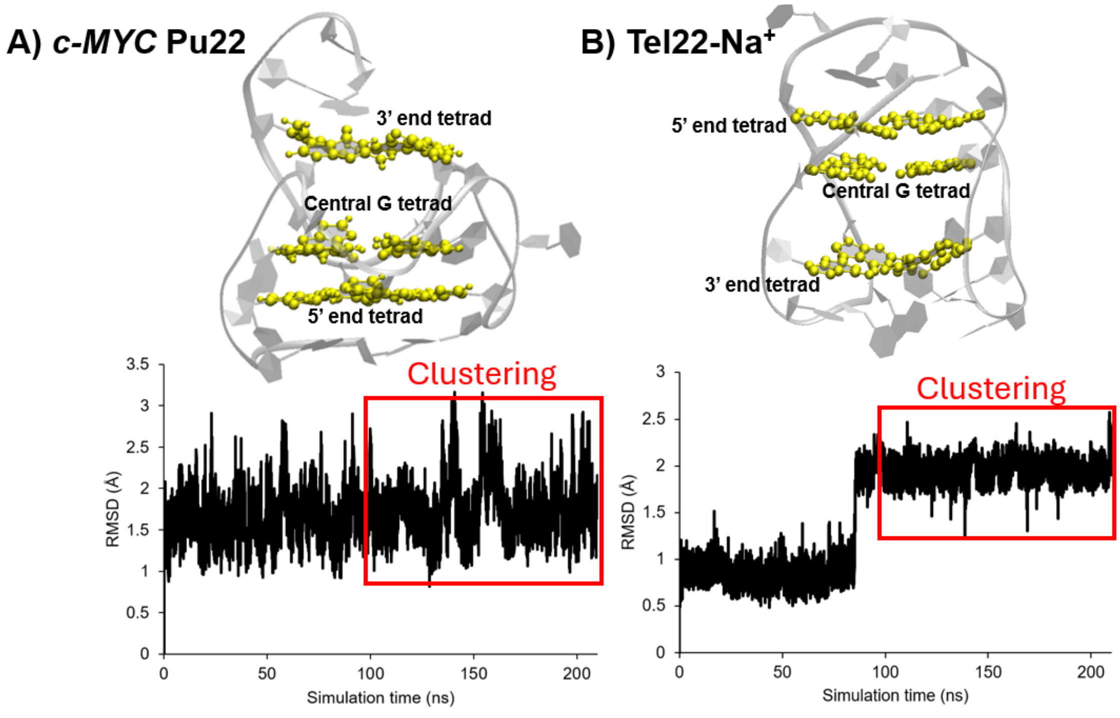

Figure S22. Representative structures and RMSD of the last window (7.5 Å) and the equilibration joined trajectory of *c-MYC* Pu22 (A) and Tel22-Na^+ (B). The representative structures are taken from the last 100 ns of the simulation (indicated in red).

The RMSD in the last 100 ns in both structures (Figures S23) oscillate between 1-3 Å. These low values indicate that the structures in the simulations are equilibrated. Hence, both serve as good receptors for the docking calculations to find the intercalative binding pockets.

6.2 DOCKING CALCULATIONS

The docking calculations were performed with DOCK 6[10] using the same parameters as in reference,[11] except for the Hawkins GB/SA rescoring, which was not performed, and the calculation of the ligand charges, which were computed quantum mechanically. Specifically, *cis* and *trans* **Py-Azo4F-3N** were built using GaussView[12] and were later optimized at the B3LYP[13-16]/cc-pVDZ[17] level of theory in implicit water solvation according to the IEFPCM[18, 19] implementation in Gaussian16.[20] Then, the restrained electrostatic potential (RESP)[21] charges were calculated at the Hartree-Fock[22]/6-31G*[23, 24] level of theory with Gaussian16. Following the calculation of the RESP charges, c*is* and *trans* **Py-Azo4F-3N** were converted to mol2 format with an antechamber.[25] Then, an additional mol2 file for each ligand was created using the *Dock Prep* tool in Chimera.[26] Finally, the RESP charges of the first mol2 file were included in the Chimera mol2 file: the final ligand mol2 files include the atom types from Chimera and the RESP charges from antechamber. To restrain the *cis* and *trans* dihedrals to their corresponding values in the optimized structure, the *flex.dfn* and the *flex_drive.tbl* files were edited to include this restriction. Regarding the receptors, the first 1XAV model (Figure S21A) and the fifth 143D model (Figure S22B) were employed to identify the external binding pockets. Similarly, the representative *c-MYC* Pu22 open structure (Figure S23A) and the representative Tel22-Na^+ open structure (Figure S22B) from the US were employed to identify the intercalative binding pockets.

1000 docking calculations were performed for each system (*cis*-intercalative *c-MYC* Pu22, *trans*-intercalative *c-MYC* Pu22, *cis*-external *c-MYC* Pu22, *trans*-external *c-MYC* Pu22, *cis*-intercalative Tel22-Na^+, *trans*-intercalative Tel22-Na^+, *cis*-external Tel22-Na^+ and *trans*-external Tel22-Na^+). Each one of them rendered 5 different geometries, resulting in a total of 5000 docking positions per system. To analyze the convergence of the results, we coded a script that divided the receptor box in 12 equivalent regions and calculated the CM of all the poses. Then, it calculated the percentage of the CM positions per region every 10 docking runs. We represented these results in Figures S23-24, where we show the percentage of CM positions per region with the number of docking runs to check convergence.

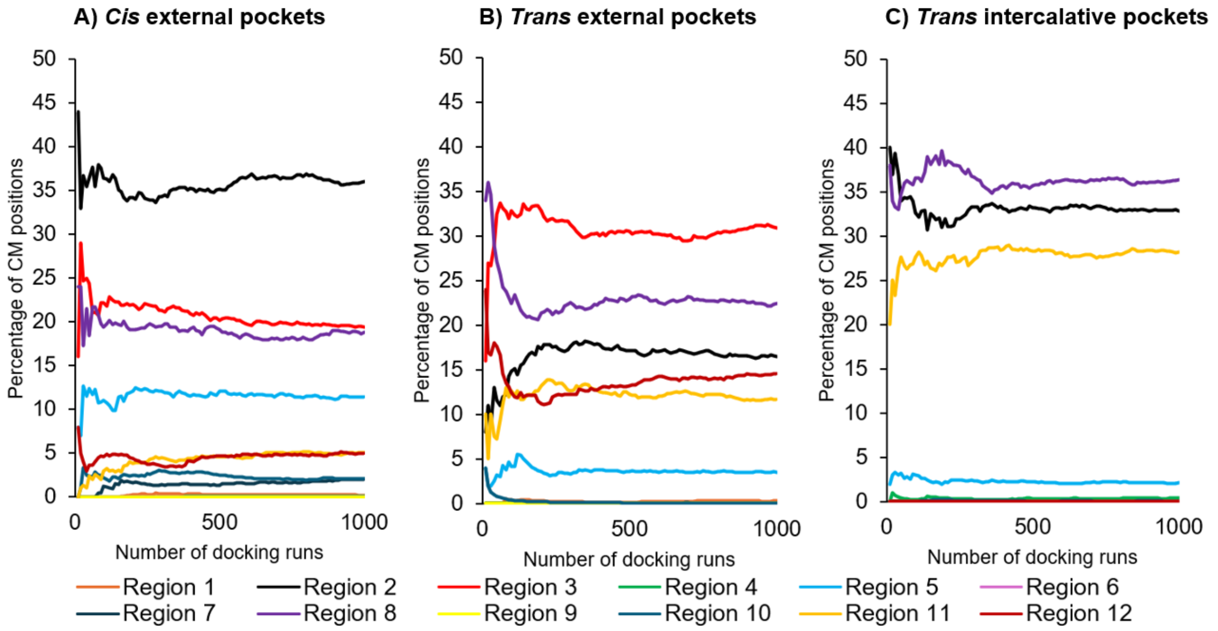

Figure S23. Convergence of the percentage of CM positions per docking runs in the *c-MYC* Pu22 calculations. There is no figure for the *cis* intercalative pockets because 99.68% of CM were in region 8.

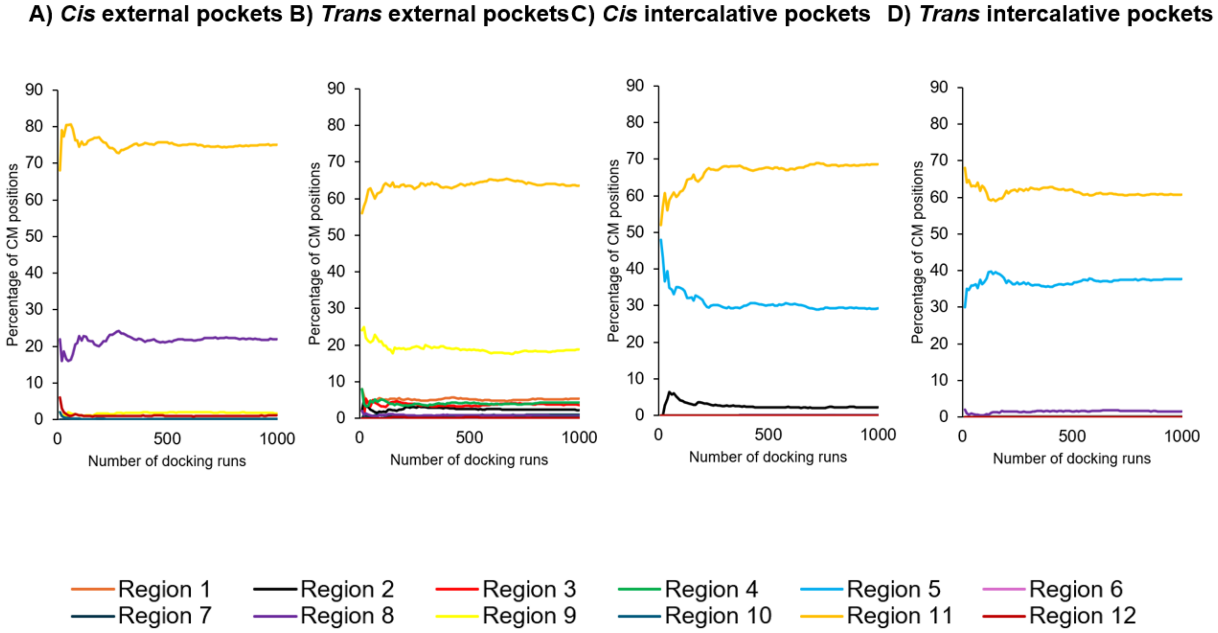

Figure S24. Convergence of the percentage of CM positions per docking runs in the Tel22-Na^+ calculations.

In all cases, the percentage of CM positions seem to converge at around 400 docking runs. As the docking calculations are fast, we run them 1000 times to generate binding pocket representative structures of statistical significance. Finally, we combined the 5000 docking positions in one single trajectory per system. The resulting trajectory was analyzed with cpptraj to get the 10 most representative structures of the docking calculations, i.e. the binding modes that represent the most common docking positions.

The following figures show the binding modes corresponding to the 5 most common docking positions of **Py-Azo4F-3N** in each system, as well as the analysis of their relative abundance (RMSD calculation).

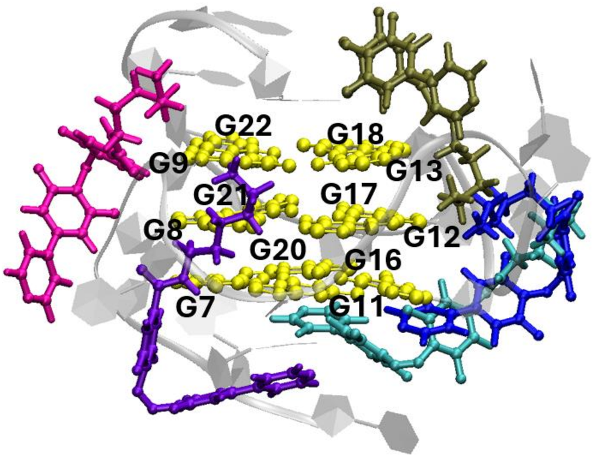

Figure S25. Most representative external docking positions of **Py-Azo4F-3N**$_{cis}$ in *c-MYC* Pu22. Pockets 1, 2, 3, 4, and 5 are represented in cyan, purple, blue, magenta and tan, respectively.

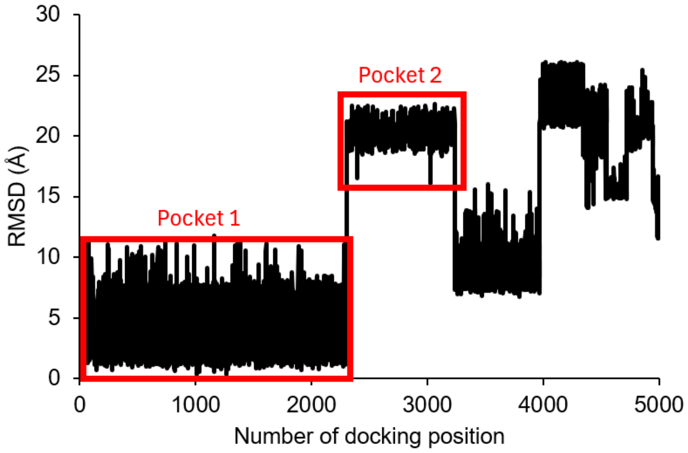

Figure S26. RMSD of the external docking positions of **Py-Azo4F-3N**$_{cis}$ in *c-MYC* Pu22 with respect to its first position in pocket 1. The values corresponding to the most abundant pockets (Pockets 1 and 2) are indicated in red.

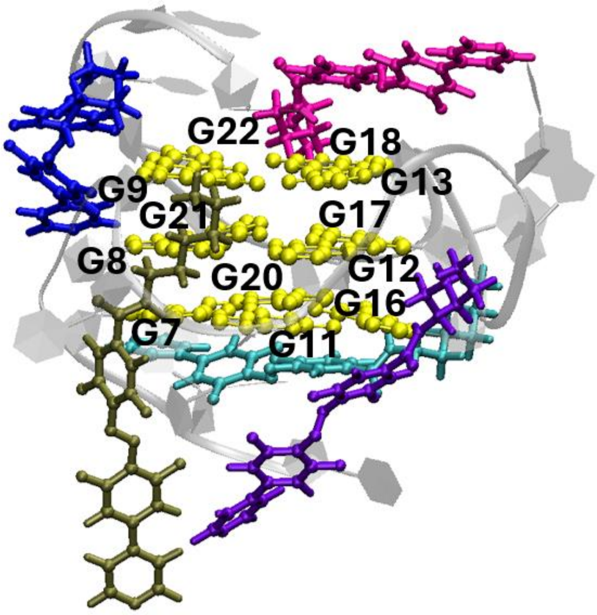

Figure S27. Most representative external docking positions of **Py-Azo4F-3N**$_{trans}$ in *c-MYC* Pu22. Pockets 1, 2, 3, 4, and 5 are represented in cyan, purple, blue, magenta and tan, respectively.

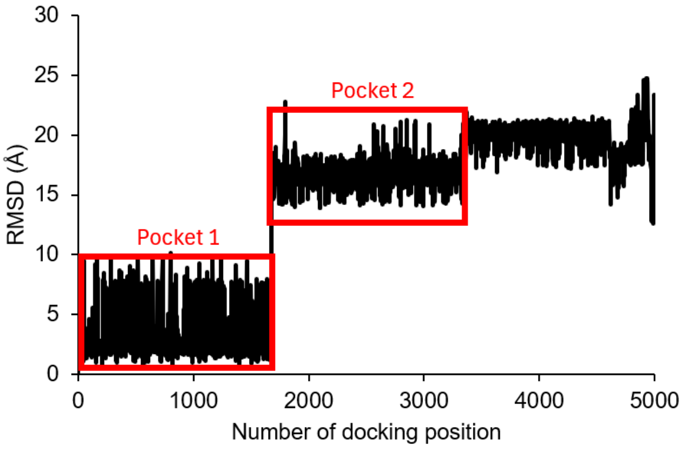

Figure S28. RMSD of the external docking positions of **Py-Azo4F-3N**$_{trans}$ in *c-MYC* Pu22 with respect to its first position in pocket 1. The values corresponding to the most abundant pockets (Pockets 1 and 2) are indicated in red.

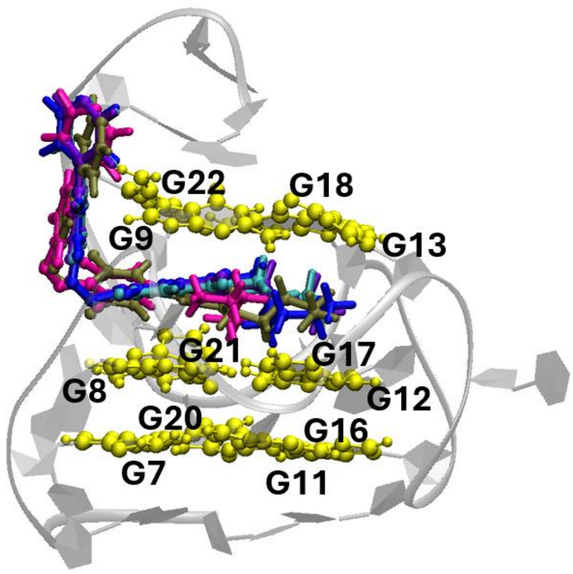

Figure S29. Most representative intercalative docking positions of **Py-Azo4F-3N**$_{cis}$ in *c-MYC* Pu22. Pockets 1, 2, 3, 4, and 5 are represented in cyan, purple, blue, magenta and tan, respectively.

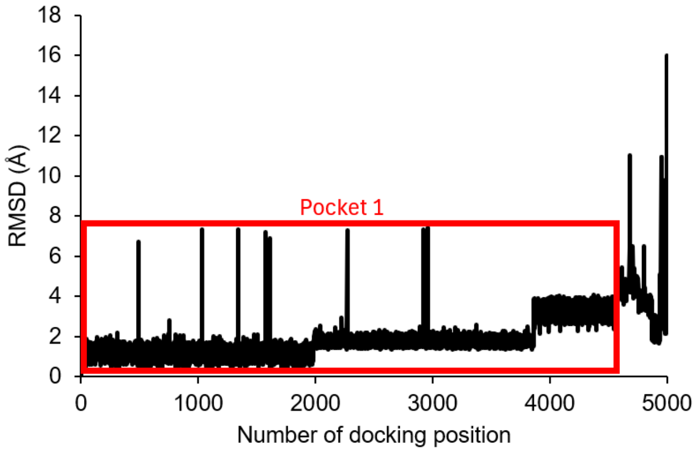

Figure S30. RMSD of the intercalative docking positions of **Py-Azo4F-3N**$_{cis}$ in *c-MYC* Pu22 with respect to its first position in pocket 1. The values corresponding to the most abundant pocket (Pocket 1) are indicated in red.

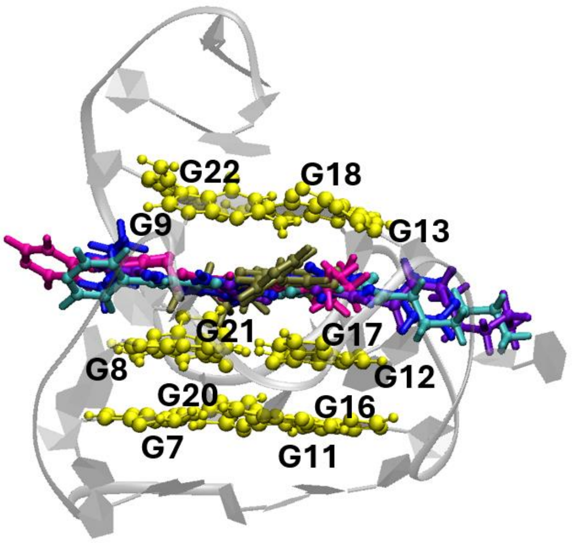

Figure S31. Most representative intercalative docking positions of **Py-Azo4F-3N***trans* in *c-MYC* Pu22. Pockets 1, 2, 3, 4 and 5 are represented in cyan, purple, blue, magenta and tan, respectively.

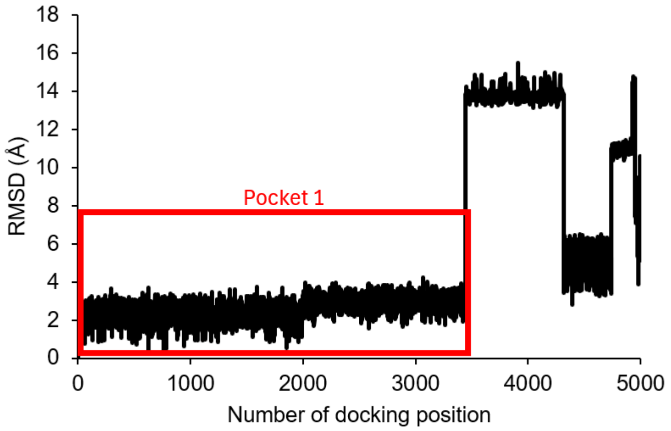

Figure S32. RMSD of the intercalative docking positions of **Py-Azo4F-3N***trans* in *c-MYC* Pu22 with respect to its first position in pocket 1. The values corresponding to the most abundant pocket (Pocket 1) are indicated in red.

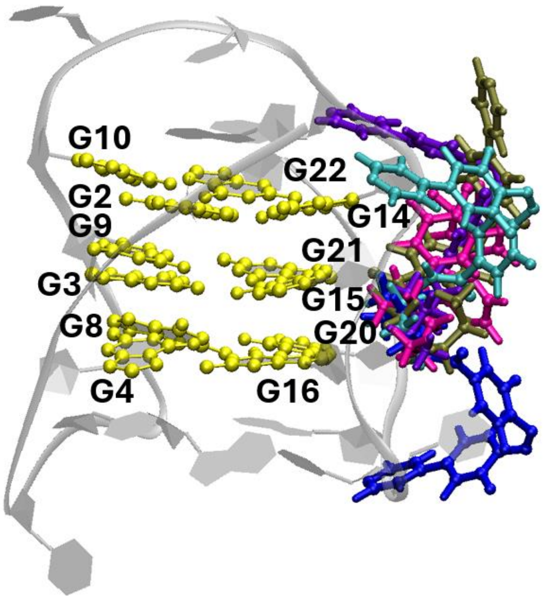

Figure S33. Most representative external docking positions of **Py-Azo4F-3N***cis* in Tel22-Na^+. Pockets 1, 2, 3, 4 and 5 are represented in cyan, purple, blue, magenta and tan, respectively.

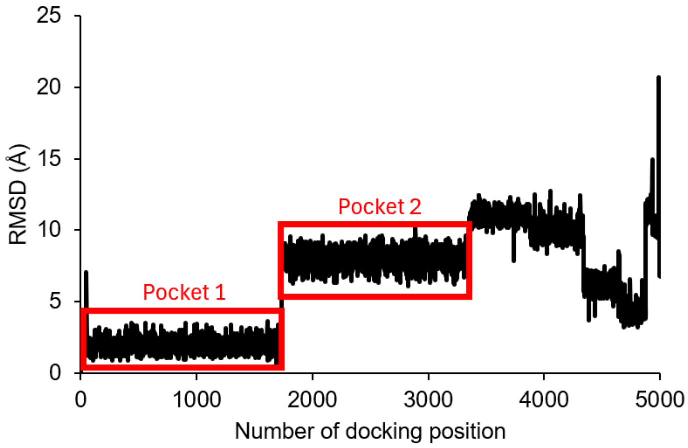

Figure S34. RMSD of the external docking positions of **Py-Azo4F-3N**$_{cis}$ in Tel22-Na$^+$ with respect to its first position in pocket 1. The values corresponding to the most abundant pockets (Pockets 1 and 2) are indicated in red.

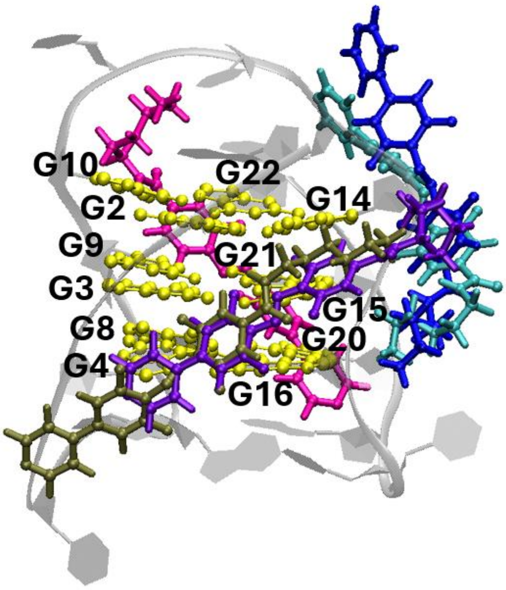

Figure S35. Most representative external docking positions of **Py-Azo4F-3N**$_{trans}$ in Tel22-Na$^+$. Pockets 1, 2, 3, 4 and 5 are represented in cyan, purple, blue, magenta and tan, respectively.

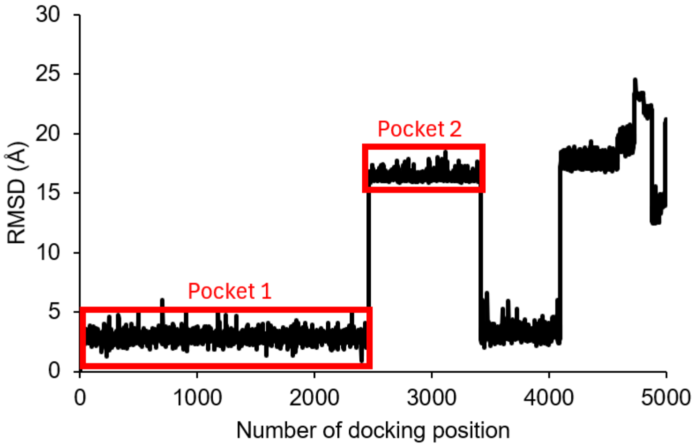

Figure S36. RMSD of the external docking positions of **Py-Azo4F-3N**$_{trans}$ in Tel22-Na$^+$ with respect to its first position in pocket 1. The values corresponding to the most abundant pockets (Pockets 1 and 2) are indicated in red.

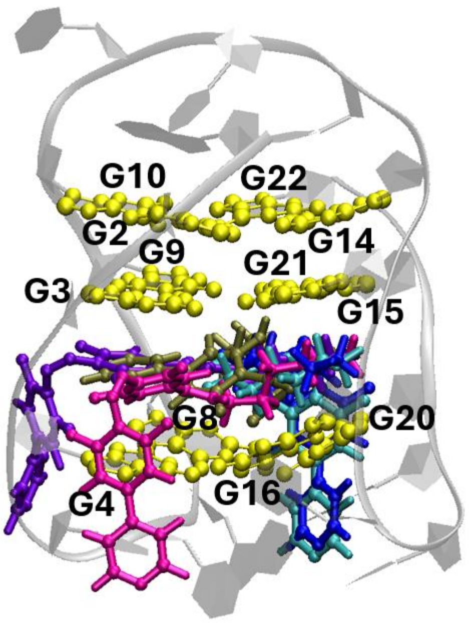

Figure S37. Most representative intercalative docking positions of **Py-Azo4F-3N**$_{cis}$ in Tel22-Na$^+$. Pockets 1, 2, 3, 4 and 5 are represented in cyan, purple, blue, magenta and tan, respectively.

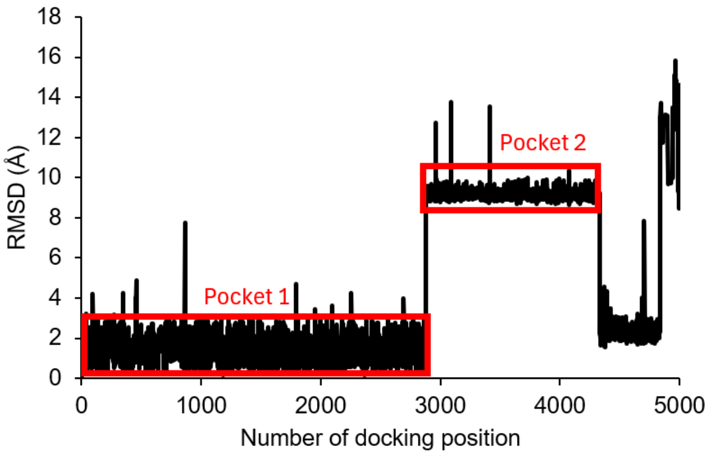

Figure S38. RMSD of the intercalative docking positions of **Py-Azo4F-3N**$_{cis}$ in Tel22-Na$^+$ with respect to its first position in pocket 1. The values corresponding to the most abundant pockets (Pockets 1 and 2) are indicated in red.

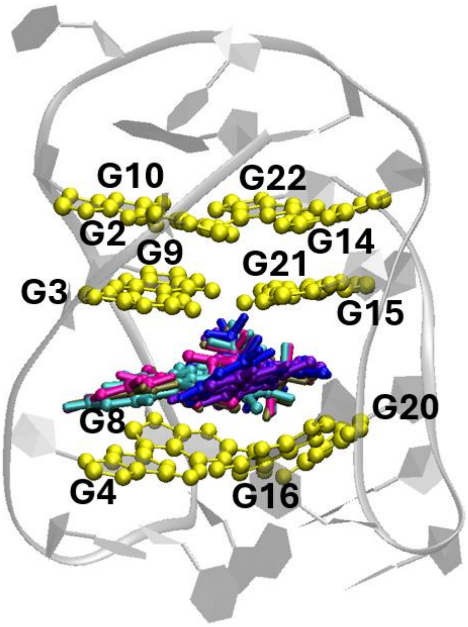

Figure S39. Most representative intercalative docking positions of **Py-Azo4F-3N**$_{trans}$ in Tel22-Na^+. Pockets 1, 2, 3, 4 and 5 are represented in cyan, purple, blue, magenta and tan, respectively.

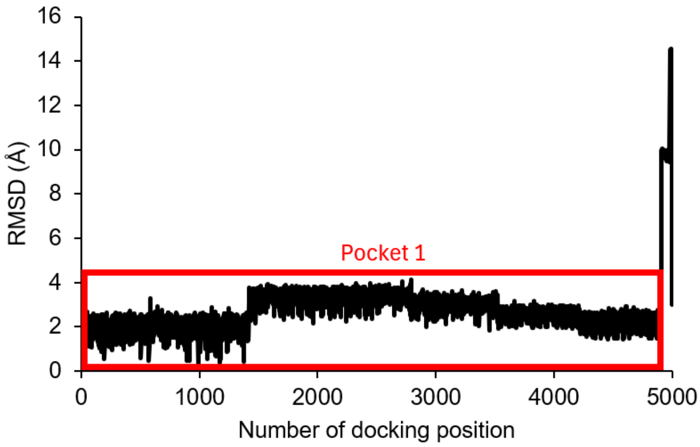

Figure S40. RMSD of the intercalative docking positions of **Py-Azo4F-3N**$_{trans}$ in Tel22-Na^+ with respect to its first position in pocket 1. The values corresponding to the most abundant pocket (Pocket 1) are indicated in red.

6.3 MOLECULAR DYNAMICS SIMULATIONS

The two most abundant external and the most abundant intercalative docking pockets were chosen as the initial geometries for the external and intercalated MD calculations, respectively. Furthermore, to investigate the intercalation of the **Py-Azo4F-3N**$_{cis}$ aromatic rings, we also selected the most common docking position in which they were intercalated in Tel22-Na^+ (5th docking mode), and we placed them manually for *c-MYC* Pu22, as there were no docking positions with the **Py-Azo4F-3N**$_{cis}$ aromatic rings intercalated. From these representative structures, the systems were prepared using *tleap* to run the dynamics. The Generalised Amber Force Field 2 (GAFF2)[27] was employed to model c*is* and *trans* **Py-Azo4F-3N**, except for the electrostatic potential charges, which were the previously calculated RESP charges. The remaining force field parameters and *tleap* options were the same as in the US system preparation.

Following the same minimization and NVT heating steps as in the US simulation, NPT calculations were run with restrictions to the motion of the ligand, which were gradually removed in 5 steps of 40 ns each, with force constants of 10, 5, 2.5, 1 and 0.5 kcal·mol^{-1}Å^{-2}. In the case of the intercalative pockets, the same restrictions were applied to the guanines in the tetrads, and an additional 40 ns NPT simulation was run with a 0.5 kcal·mol^{-1}Å^{-2} restriction on the tetrad guanines and no restrictions on the ligand motion. Then, a 2 µs NPT production was calculated. The *cis* and *trans* azo dihedrals were restrained according to reference[28] in all MD simulations. The remaining parameters were the same as in the equilibration preceding the US. The analysis of the trajectories, including the RMSD and the clustering, were performed with cpptraj. Moreover, VMD[29] was used to generate the computational figures in this work.

The last 1µs of the NPT production runs, with a 2-frame step (a total of 50000 frames), were employed to calculate the binding free energy of the resulting pockets by using the Molecular Mechanics Generalized Born Surface Area (MMGBSA) approach. [30] Furthermore, an energy decomposition analysis was performed including all the G4 nucleobases. To check the convergence, the same procedure was repeated for the last 800 ns, 600 ns, 400 ns, 200 ns, 160 ns, 120 ns, 80 ns, 60 ns, 40 ns and 20 ns (Figure S41).

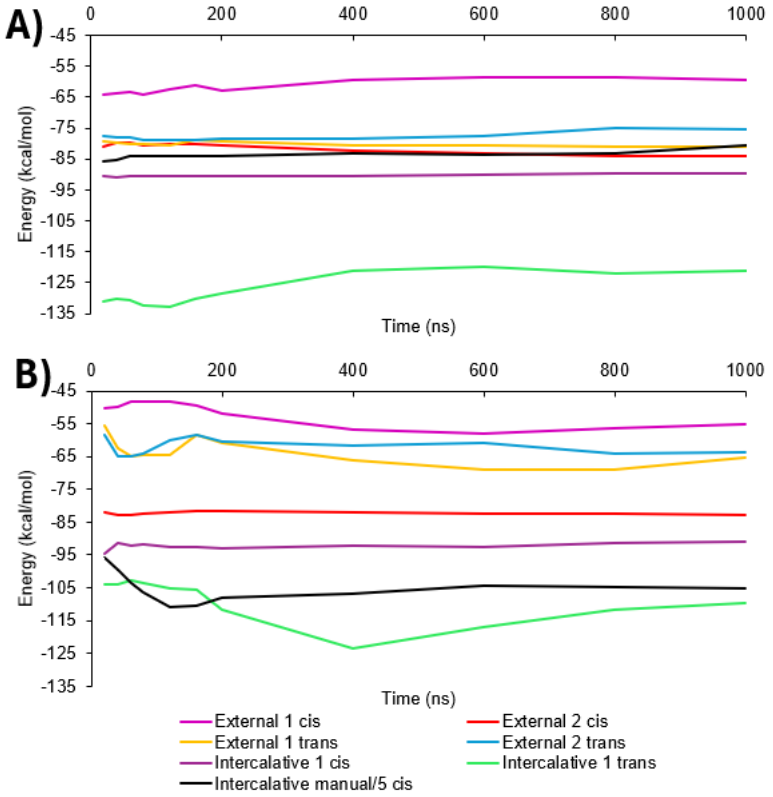

Figure S41. Convergence of the free energy when using the last 1000, 800, 600, 400, 200, 160, 120, 80, 60, 40 and 20 ns of the 2 µs production simulation in *c-MYC* Pu22 (A) and Tel22-Na$^+$ (B).

In all cases, the energy converged at around 600 ns, indicating that 1000 ns seems enough to obtain converged results. The following figures show the representative structures and the total MMGBSA energy of the last 1000 ns of the 2 µs simulations. In the representative structures of the most favourable external and intercalative poses, we show the 6 most contributing nucleotides according to the MMGBSA analysis in blue, purple, and magenta for the guanines, thymines, and adenines, respectively. Tables containing the MMGBSA energy decomposition are also included for these cases. Finally, we also show the RMSD analysis of **Py-Azo4F-3N**

and the corresponding G4 structure along the simulation time to check the stability of the pockets. In most cases, the RMSD equilibrates prior to 1000 ns, indicating that the pockets are stable.

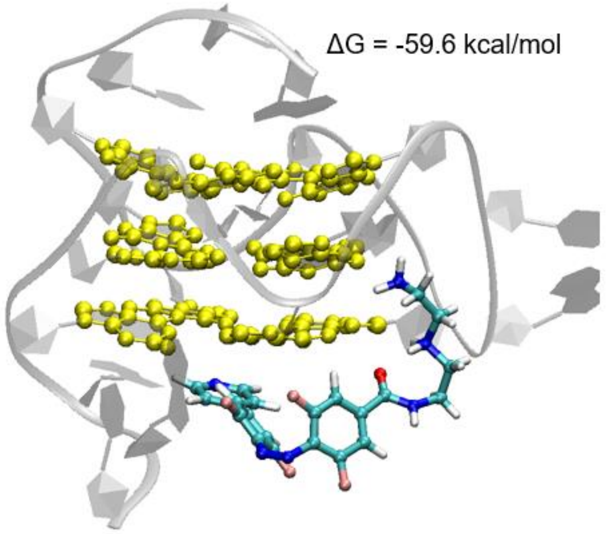

Figure S42. Representative MD structure and MMGBSA binding energy of the external **Py-Azo4F-3N**$_{cis}$ Pocket 1 in *c-MYC* Pu22.

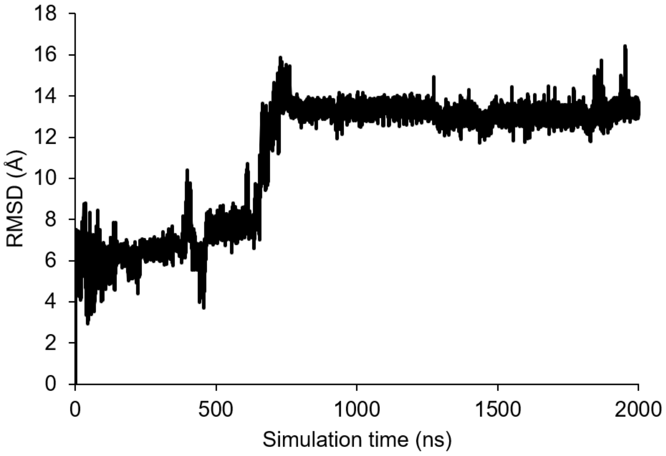

Figure S43. RMSD of **Py-Azo4F-3N** with respect to its docking pose in the external **Py-Azo4F-3N**$_{cis}$ Pocket 1 in *c-MYC* Pu22.

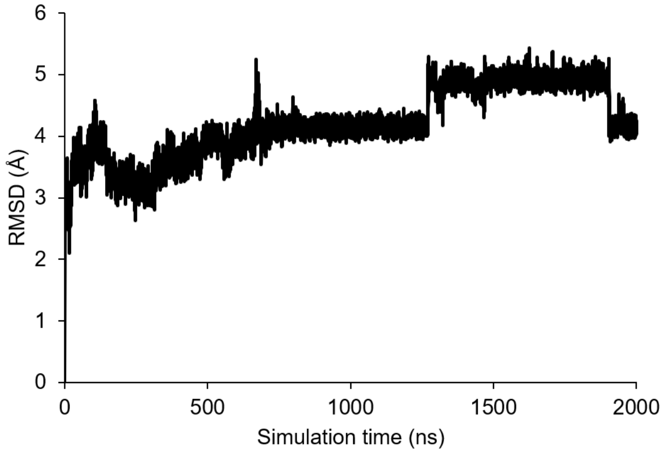

Figure S44. RMSD of *c-MYC* Pu22 in the external **Py-Azo4F-3N**$_{cis}$ Pocket 1 with respect to 1XAV.

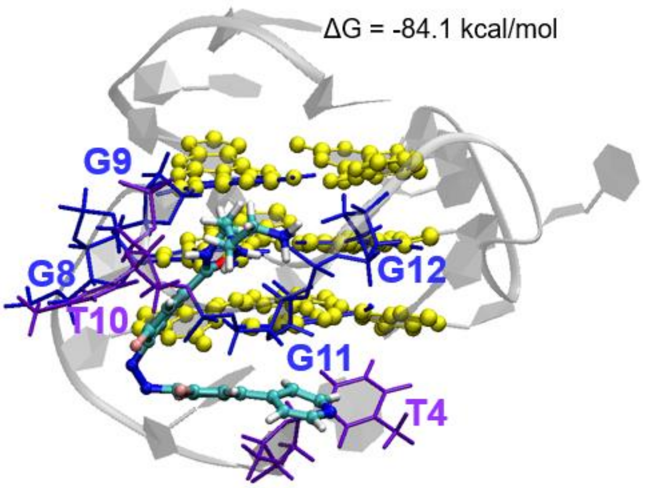

Figure S45. Representative MD structure and MMGBSA binding energy of the external **Py-Azo4F-3N**$_{cis}$ Pocket 2 in *c-MYC* Pu22. The 6 most contributing residues according to the MMGBSA analysis are indicated in blue, purple, and magenta for G, T, and A, respectively.

Table S4. MMGBSA energy decomposition of the 6 most contributing residues in the external **Py-Azo4F-3N**$_{cis}$ Pocket 2 in *c-MYC* Pu22. Energies in kcal/mol.

	**ΔG$_{vdW}$**	**ΔG$_{el}$**	**ΔG$_{pol}$**	**ΔG$_{nonpol}$**	**ΔG$_{tot}$**
G11	-21.32	-172.39	125.40	-10.66	-78.97
T10	-8.65	-139.38	102.87	-4.72	-49.89
G12	-3.78	-154.34	118.54	-1.99	-41.58
G9	-7.40	-69.98	63.68	-3.78	-17.48
G8	-3.26	-43.50	37.96	-2.39	-11.19
T4	-3.92	-13.83	13.36	-2.86	-7.26

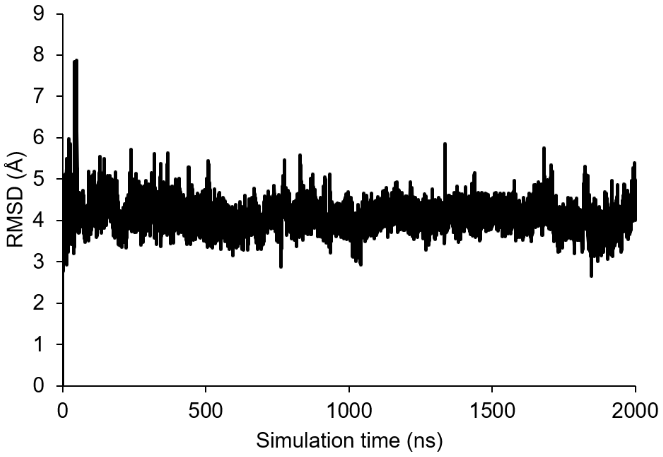

Figure S46. RMSD of **Py-Azo4F-3N** with respect to its docking pose in the external **Py-Azo4F-3N**$_{cis}$ Pocket 2 in *c-MYC* Pu22.

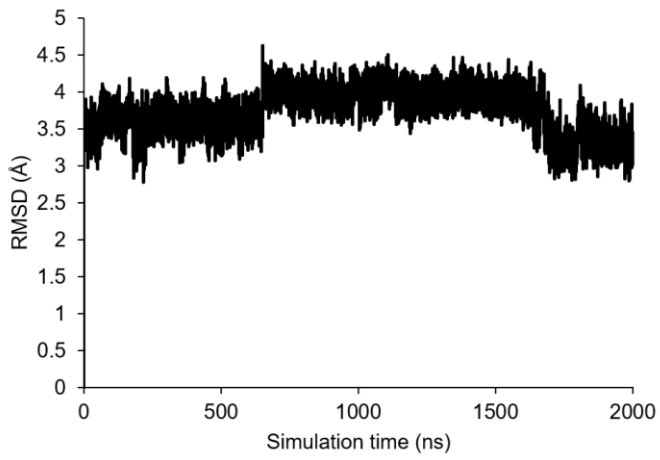

Figure S47. RMSD of *c-MYC* Pu22 in the external **Py-Azo4F-3N**$_{cis}$ Pocket 2 with respect to 1XAV.

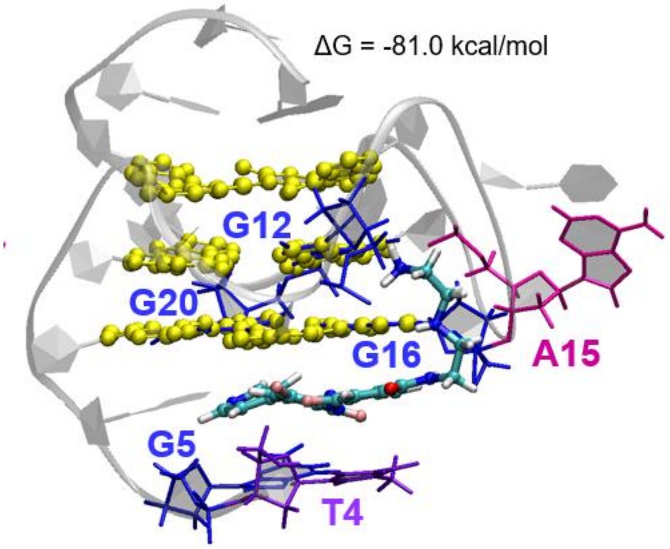

Figure S48. Representative MD structure and MMGBSA binding energy of the external **Py-Azo4F-3N**$_{trans}$ Pocket 1 in *c-MYC* Pu22. The 6 most contributing residues according to the MMGBSA analysis are indicated in blue, purple, and magenta for G, T, and A, respectively.

Table S5. MMGBSA energy decomposition of the 6 most contributing residues in the external **Py-Azo4F-3N**$_{trans}$ Pocket 1 in *c-MYC* Pu22. Energies in kcal/mol.

	**ΔG$_{vdW}$**	**ΔG$_{el}$**	**ΔG$_{pol}$**	**ΔG$_{nonpol}$**	**ΔG$_{tot}$**
A15	-6.47	-180.51	137.60	-3.04	-52.42
G16	-13.25	-139.30	111.67	-6.82	-47.69
G12	-8.09	-102.07	84.36	-3.17	-28.97
T4	-10.05	-17.67	17.39	-5.55	-15.88
G5	-9.50	-44.37	43.24	-4.92	-15.56
G20	-8.63	-50.01	49.73	-4.36	-13.27

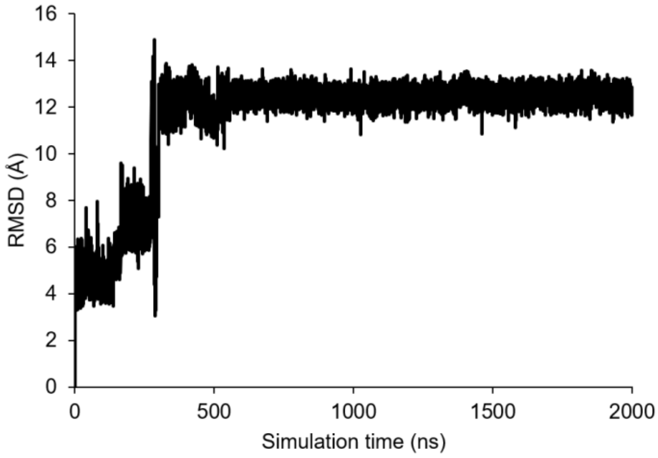

Figure S49. RMSD of **Py-Azo4F-3N** with respect to its docking pose in the external **Py-Azo4F-3N**$_{trans}$ Pocket 1 in *c-MYC* Pu22.

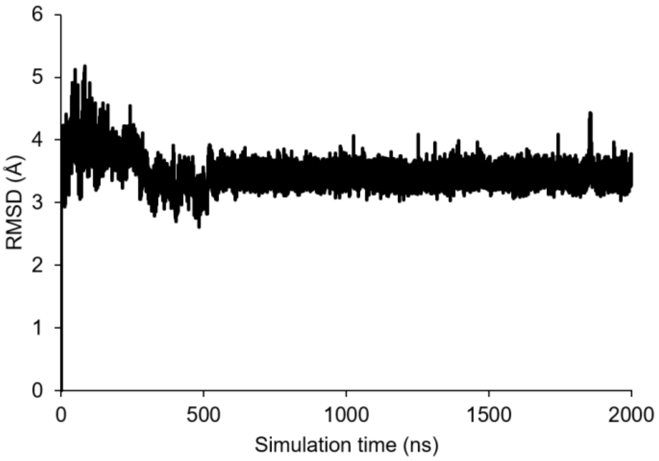

Figure S50. RMSD of *c-MYC* Pu22 in the external **Py-Azo4F-3N**$_{trans}$ Pocket 1 with respect to 1XAV.

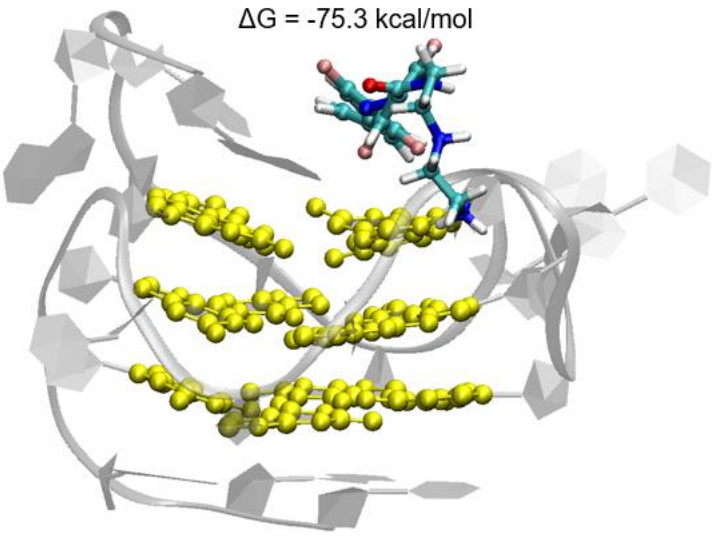

Figure S51. Representative MD structure and MMGBSA binding energy of the external **Py-Azo4F-3N**$_{trans}$ Pocket 2 in *c-MYC* Pu22.

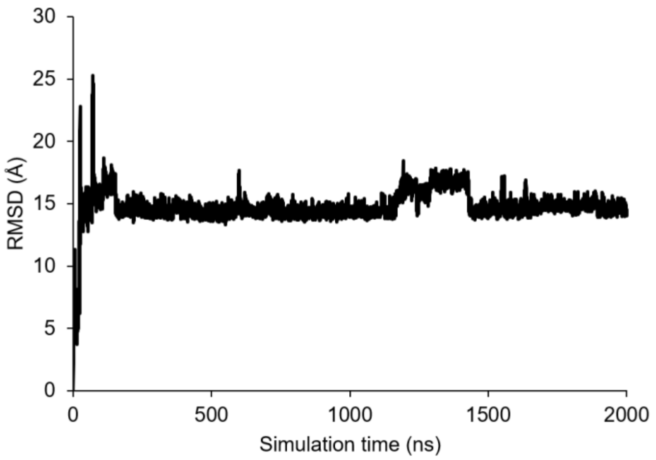

Figure S52. RMSD of **Py-Azo4F-3N** with respect to its docking pose in the external **Py-Azo4F-3N**$_{trans}$ Pocket 2 in *c-MYC* Pu22.

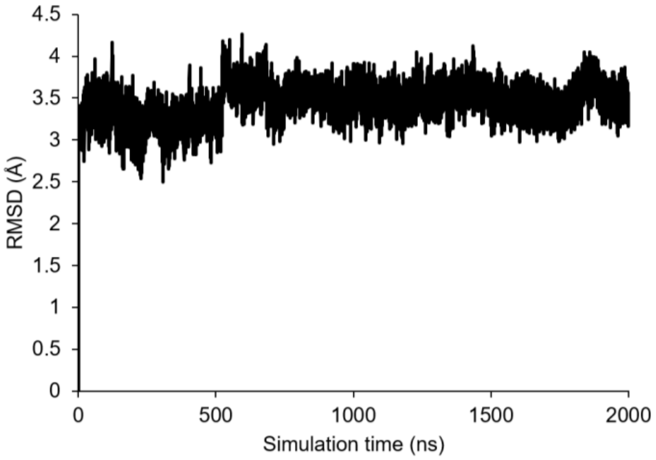

Figure S53. RMSD of *c-MYC* Pu22 in the external **Py-Azo4F-3N**$_{trans}$ Pocket 2 with respect to 1XAV.

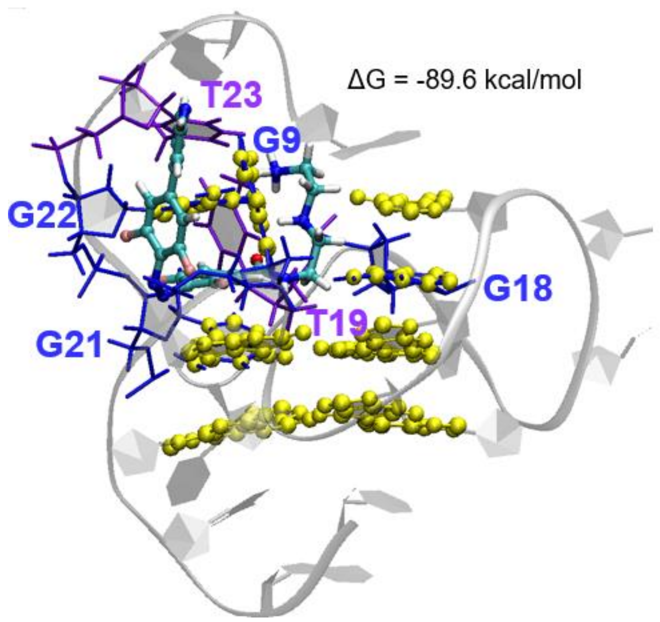

Figure S54. Representative MD structure and MMGBSA binding energy of the intercalative **Py-Azo4F-3N**$_{cis}$ Pocket 1 in *c-MYC* Pu22. The 6 most contributing residues according to the MMGBSA analysis are indicated in blue, purple, and magenta for G, T, and A, respectively.

Table S6. MMGBSA energy decomposition of the 6 most contributing residues in the intercalative **Py-Azo4F-3N***cis* Pocket 1 in *c-MYC* Pu22. Energies in kcal/mol.

	ΔG_{vdW}	ΔG_{el}	ΔG_{pol}	ΔG_{nonpol}	ΔG_{tot}
G22	-14.93	-105.54	78.20	-8.20	-50.47
T19	-5.21	-141.62	102.94	-2.63	-46.52
G18	-7.18	-96.25	68.24	-3.61	-38.80
G21	-10.90	-68.19	66.15	-5.13	-18.07
G9	-9.99	-62.00	59.96	-5.21	-17.23
T23	-6.53	-79.89	72.69	-3.44	-17.18

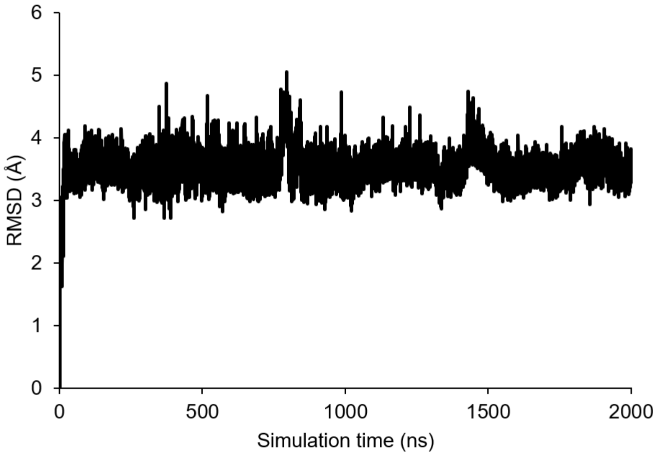

Figure S55. RMSD of **Py-Azo4F-3N** with respect to its docking pose in the intercalative **Py-Azo4F-3N***cis* Pocket 1 in *c-MYC* Pu22.

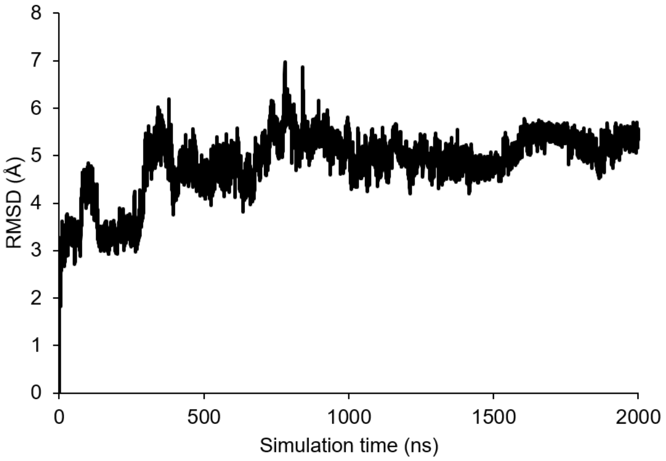

Figure S56. RMSD of *c-MYC* Pu22 in the intercalative **Py-Azo4F-3N***cis* Pocket 1 with respect to 1XAV.

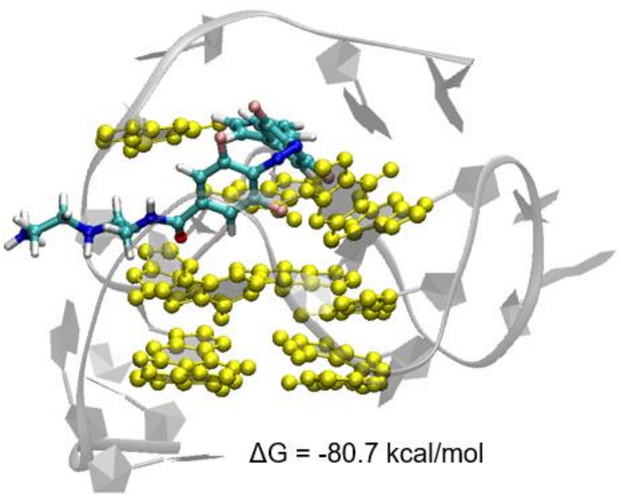

Figure S57. Representative MD structure and MMGBSA binding energy of the **Py-Azo4F-3N**$_{cis}$ Pocket in *c-MYC* Pu22 in which the **Py-Azo4F-3N**$_{cis}$ aromatic rings were manually intercalated.

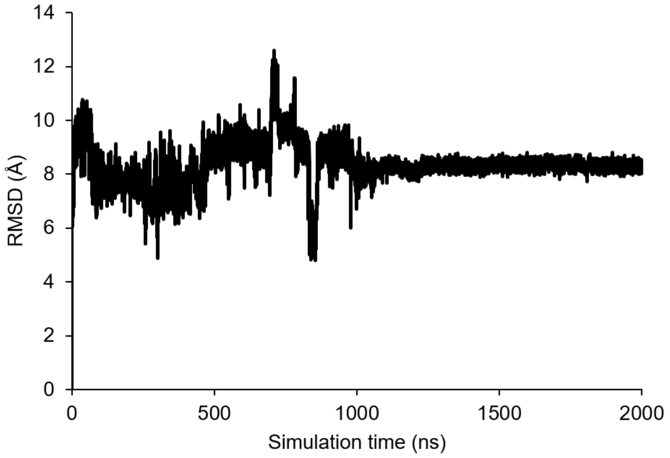

Figure S58. RMSD of **Py-Azo4F-3N** with respect the structure in which the **Py-Azo4F-3N**$_{cis}$ aromatic rings were manually intercalated in *c-MYC* Pu22.

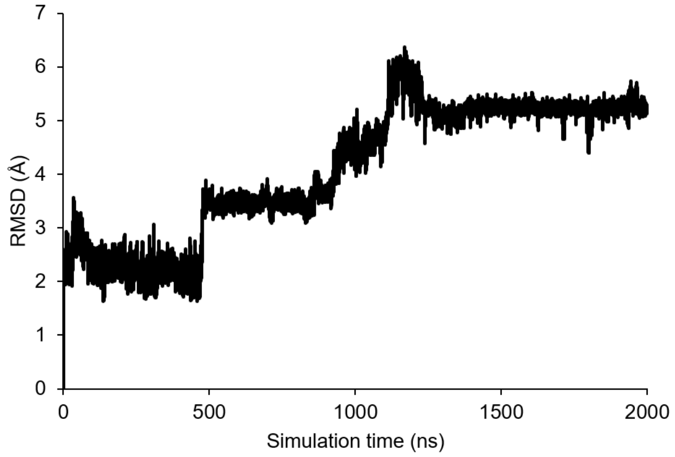

Figure S59. RMSD of *c-MYC* Pu22 in the **Py-Azo4F-3N**$_{cis}$ Pocket in which the **Py-Azo4F-3N**$_{cis}$ aromatic rings were manually intercalated with respect to 1XAV.

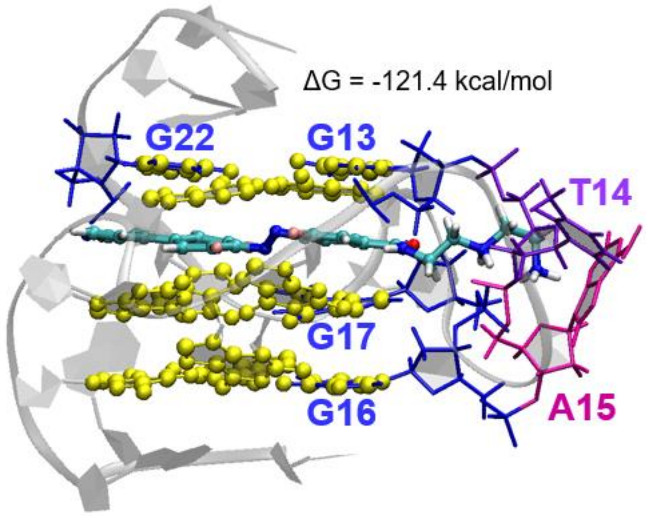

Figure S60. Representative MD structure and MMGBSA binding energy of the intercalative **Py-Azo4F-3N**$_{trans}$ Pocket 1 in *c-MYC* Pu22. The 6 most contributing residues according to the MMGBSA analysis are indicated in blue, purple and magenta for G, T and A, respectively.

Table S7. MMGBSA energy decomposition of the 6 most contributing residues in the intercalative **Py-Azo4F-3N**$_{trans}$ Pocket 1 in *c-MYC* Pu22. Energies in kcal/mol.

	**ΔG$_{vdW}$**	**ΔG$_{el}$**	**ΔG$_{pol}$**	**ΔG$_{nonpol}$**	**ΔG$_{tot}$**
A15	-6.61	-168.03	115.00	-3.24	-62.88
G17	-11.68	-155.56	112.65	-6.04	-60.64
G16	-3.84	-120.32	93.60	-1.54	-32.10
G13	-12.35	-79.09	70.11	-6.08	-27.41
T14	-3.91	-108.52	90.53	-1.69	-23.59
G22	-9.86	-35.01	34.61	-5.12	-15.38

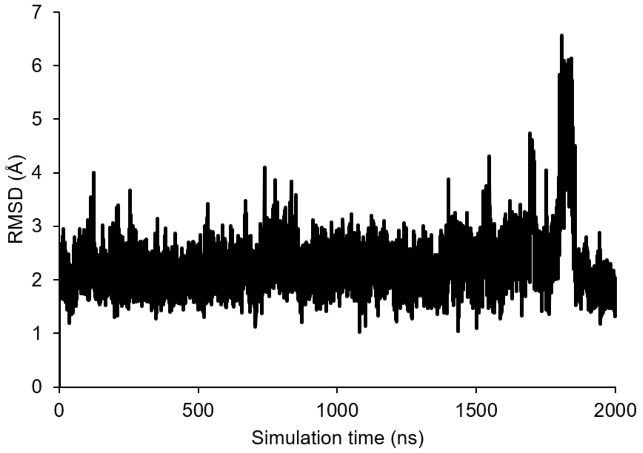

Figure S61. RMSD of **Py-Azo4F-3N** with respect to its docking pose in the intercalative **Py-Azo4F-3N**$_{trans}$ Pocket 1 in *c-MYC* Pu22.

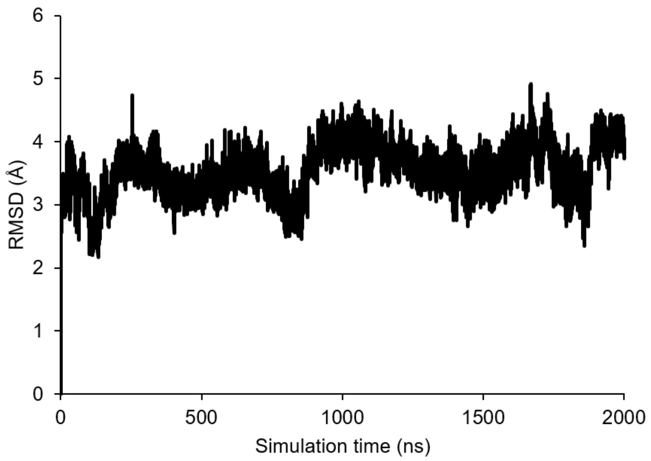

Figure S62. RMSD of *c-MYC* Pu22 in the intercalative **Py-Azo4F-3N**$_{trans}$ Pocket 1 with respect to 1XAV.

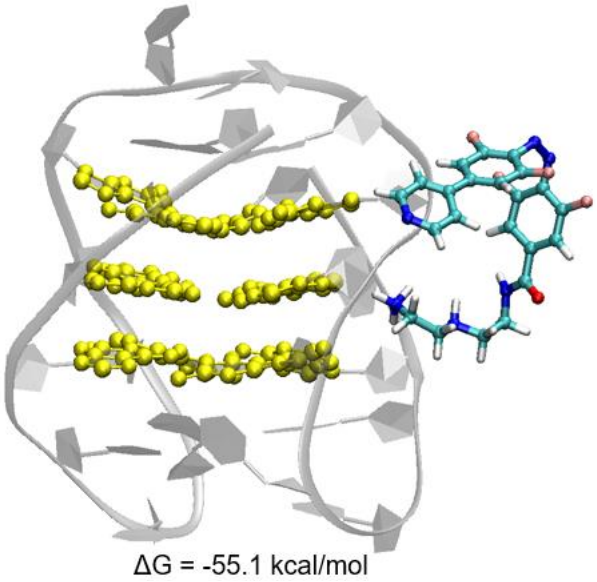

Figure S63. Representative MD structure and MMGBSA binding energy of the external **Py-Azo4F-3N**$_{cis}$ Pocket 1 in Tel22-Na^+.

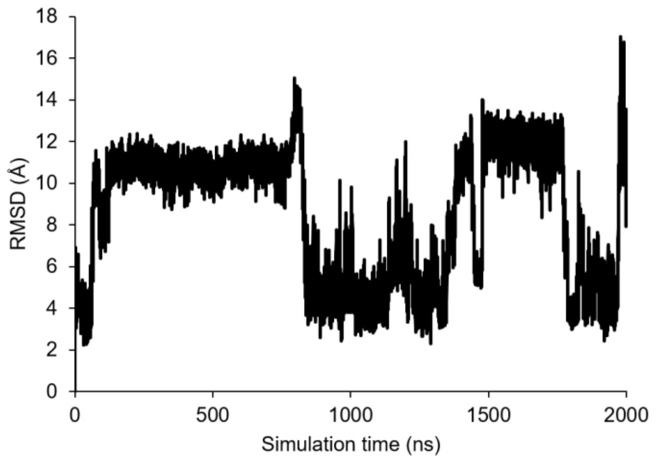

Figure S64. RMSD of **Py-Azo4F-3N** with respect to its docking pose in the external **Py-Azo4F-3N**$_{cis}$ Pocket 1 in Tel22-Na^+.

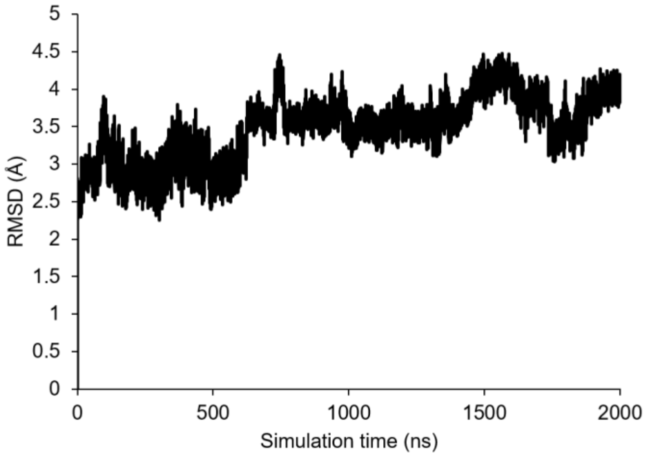

Figure S65. RMSD of Tel22-Na$^+$ in the external **Py-Azo4F-3N**$_{cis}$ Pocket 1 with respect to 143D.

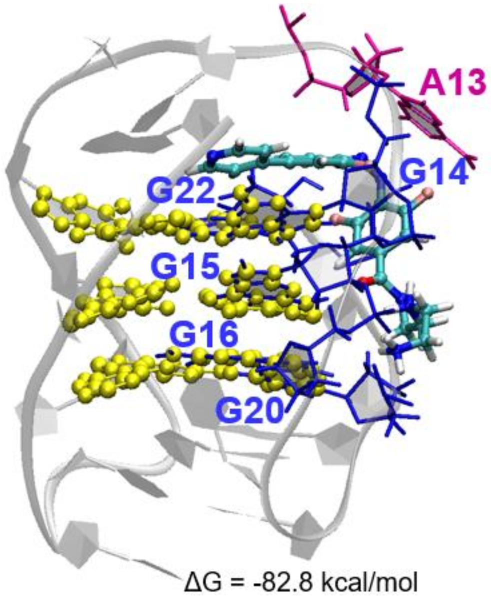

Figure S66. Representative MD structure and MMGBSA binding energy of the external **Py-Azo4F-3N**$_{cis}$ Pocket 2 in Tel22-Na$^+$. The 6 most contributing residues according to the MMGBSA analysis are indicated in blue, purple and magenta for G, T and A, respectively.

Table S8. MMGBSA energy decomposition of the 6 most contributing residues in the external **Py-Azo4F-3N**$_{cis}$ Pocket 2 in Tel22-Na$^+$. Energies in kcal/mol.

	ΔG_{vdW}	ΔG_{el}	ΔG_{pol}	ΔG_{nonpol}	ΔG_{tot}
G20	-7.85	-158.01	117.98	-4.06	-51.94
G15	-7.92	-149.76	123.71	-3.89	-37.87
G14	-15.50	-73.33	68.21	-8.15	-28.76
G16	-1.58	-141.74	116.95	-1.30	-27.68
A13	-9.62	-46.41	44.89	-5.42	-16.57
G22	-7.06	-28.99	28.48	-4.37	-11.94

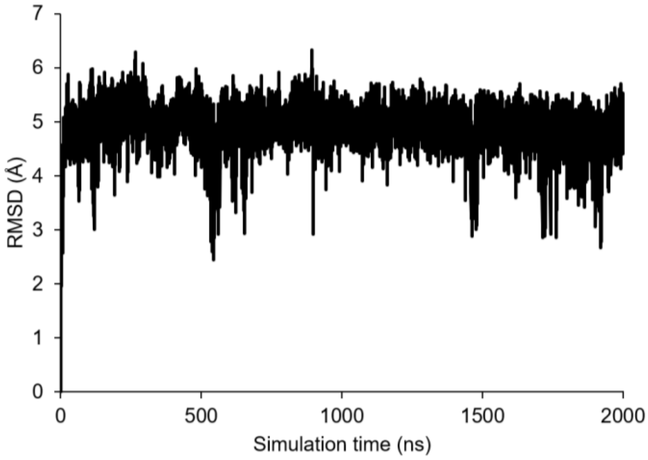

Figure S67. RMSD of **Py-Azo4F-3N** with respect to its docking pose in the external **Py-Azo4F-3N**$_{cis}$ Pocket 2 in Tel22-Na$^+$.

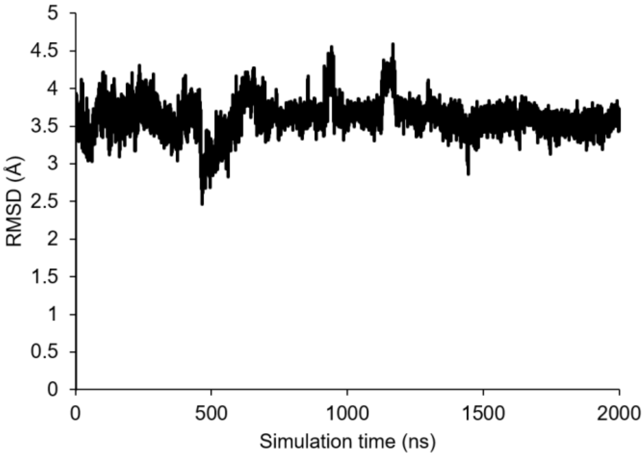

Figure S68. RMSD of Tel22-Na$^+$ in the external **Py-Azo4F-3N**$_{cis}$ Pocket 2 with respect to 143D.

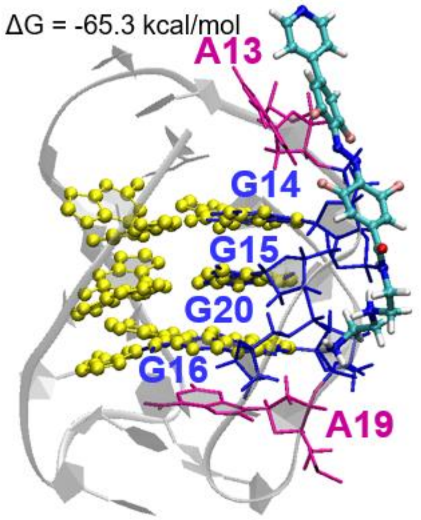

Figure S69. Representative MD structure and MMGBSA binding energy of the external **Py-Azo4F-3N**$_{trans}$ Pocket 1 in Tel22-Na$^+$. The 6 most contributing residues according to the MMGBSA analysis are indicated in blue, purple and magenta for G, T and A, respectively.

Table S9. MMGBSA energy decomposition of the 6 most contributing residues in the external **Py-Azo4F-3N**$_{trans}$ Pocket 1 in Tel22-Na^+. Energies in kcal/mol.

	ΔG_{vdW}	ΔG_{el}	ΔG_{pol}	ΔG_{nonpol}	ΔG_{tot}
G15	-10.84	-180.81	129.27	-5.55	-67.93
G16	-3.26	-158.96	119.47	-2.02	-44.77
G20	-1.95	-127.16	105.09	-1.34	-25.36
A13	-8.26	-48.48	47.17	-4.75	-14.32
G14	-4.91	-77.09	70.32	-2.53	-14.21
A19	-1.04	-81.80	76.80	-0.50	-6.53

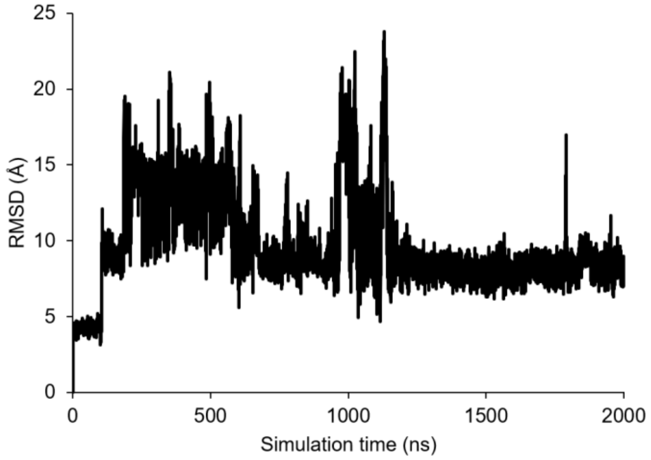

Figure S70. RMSD of **Py-Azo4F-3N** with respect to its docking pose in the external **Py-Azo4F-3N**$_{trans}$ Pocket 1 in Tel22-Na^+.

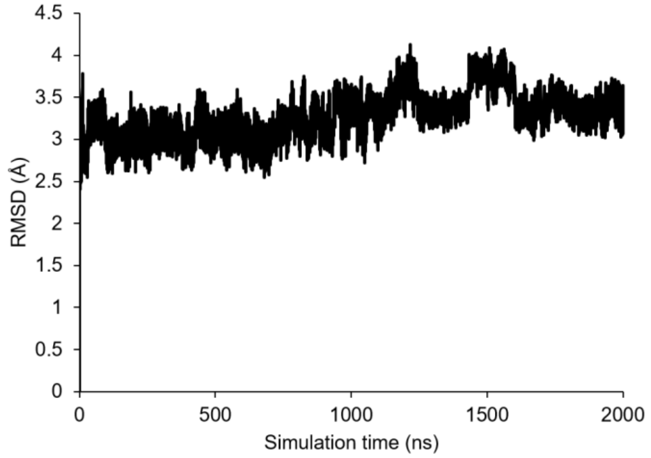

Figure S71. RMSD of Tel22-Na^+ in the external **Py-Azo4F-3N**$_{trans}$ Pocket 1 with respect to 143D.

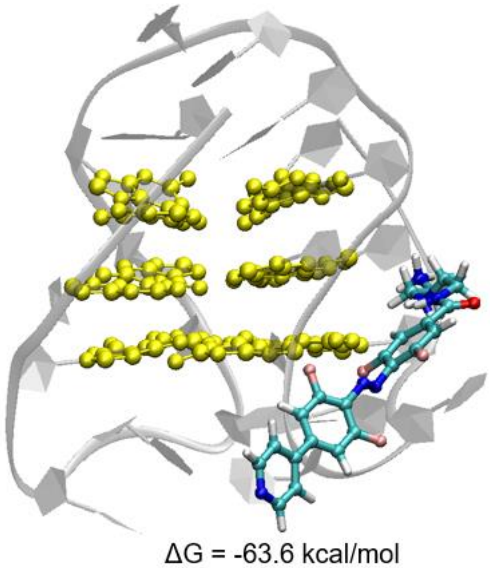

Figure S72. Representative MD structure and MMGBSA binding energy of the external **Py-Azo4F-3N***trans* Pocket 2 in Tel22-Na^+.

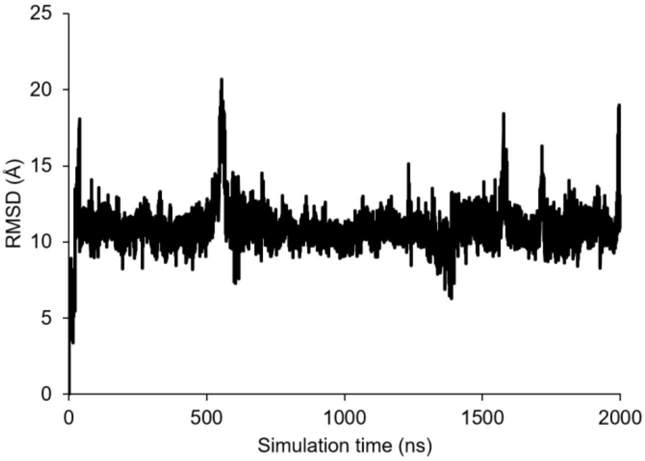

Figure S73. RMSD of **Py-Azo4F-3N** with respect to its docking pose in the external **Py-Azo4F-3N***trans* Pocket 2 in Tel22-Na^+.

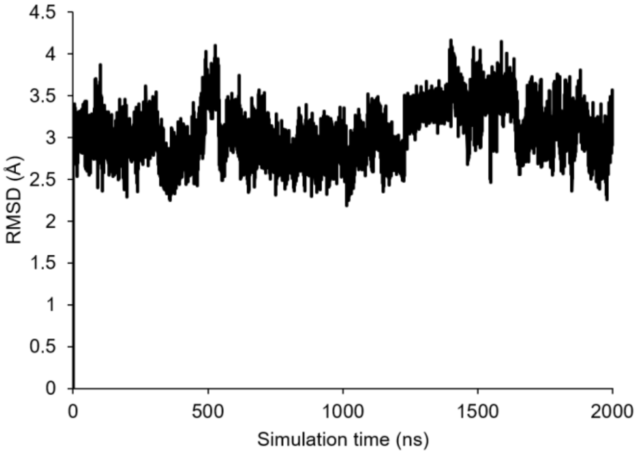

Figure S74. RMSD of Tel22-Na^+ in the external **Py-Azo4F-3N***trans* Pocket 2 with respect to 143D.

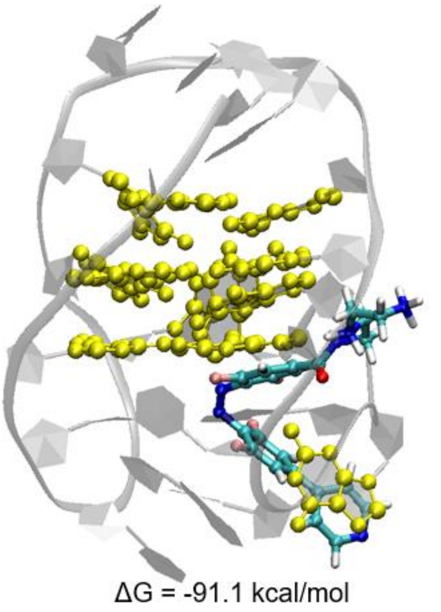

Figure S75. Representative MD structure and MMGBSA binding energy of the intercalative **Py-Azo4F-3N**$_{cis}$ Pocket 1 in Tel22-Na^+.

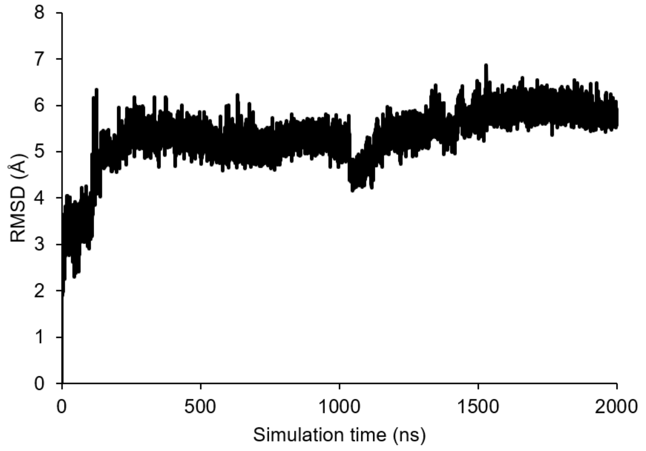

Figure S76. RMSD of **Py-Azo4F-3N** with respect to its docking pose in the intercalative **Py-Azo4F-3N**$_{cis}$ Pocket 1 in Tel22-Na^+.

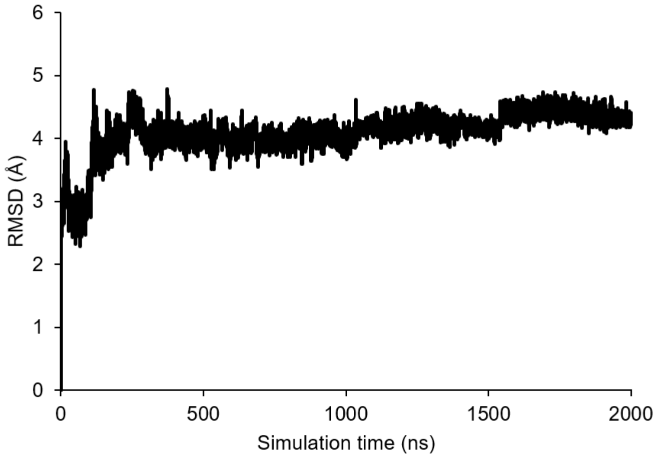

Figure S77. RMSD of Tel22-Na^+ in the intercalative **Py-Azo4F-3N**$_{cis}$ Pocket 1 with respect to 143D.

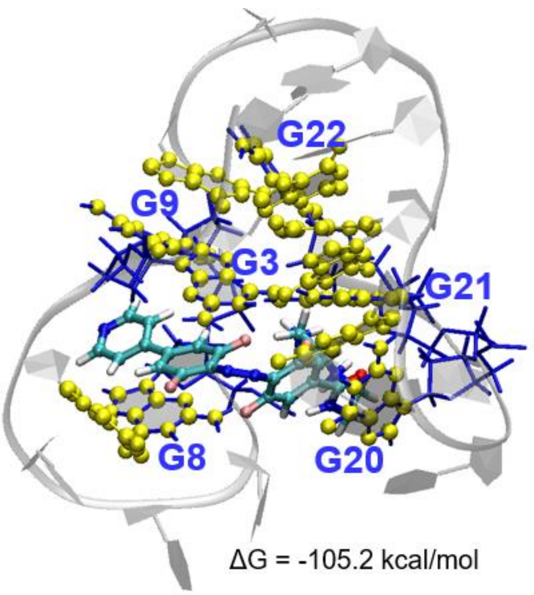

Figure S78. Representative MD structure and MMGBSA binding energy of the intercalative **Py-Azo4F-3N**$_{cis}$ Pocket 5 in Tel22-Na$^+$. The 6 most contributing residues according to the MMGBSA analysis are indicated in blue, purple and magenta for G, T and A, respectively.

Table S10. MMGBSA energy decomposition of the 6 most contributing residues in the intercalative **Py-Azo4F-3N**$_{cis}$ Pocket 5 in Tel22-Na$^+$. Energies in kcal/mol.

	**ΔG$_{vdW}$**	**ΔG$_{el}$**	**ΔG$_{pol}$**	**ΔG$_{nonpol}$**	**ΔG$_{tot}$**
G8	-16.50	-184.73	123.91	-9.31	-86.63
G9	-3.92	-150.08	115.10	-2.24	-41.15
G22	-5.07	-110.64	80.10	-2.51	-38.12
G21	-12.28	-88.91	72.43	-6.61	-35.37
G3	-9.34	-49.78	48.59	-4.70	-15.23
G20	-8.81	-57.60	55.92	-4.57	-15.07

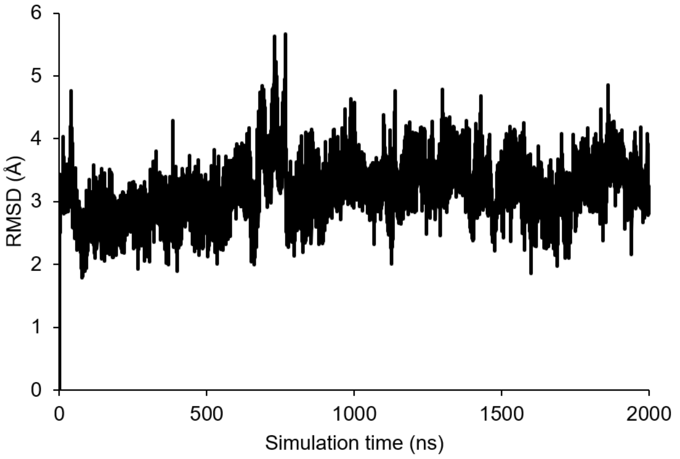

Figure S79. RMSD of **Py-Azo4F-3N** with respect to its docking pose in the intercalative **Py-Azo4F-3N**$_{cis}$ Pocket 5 in Tel22-Na$^+$.

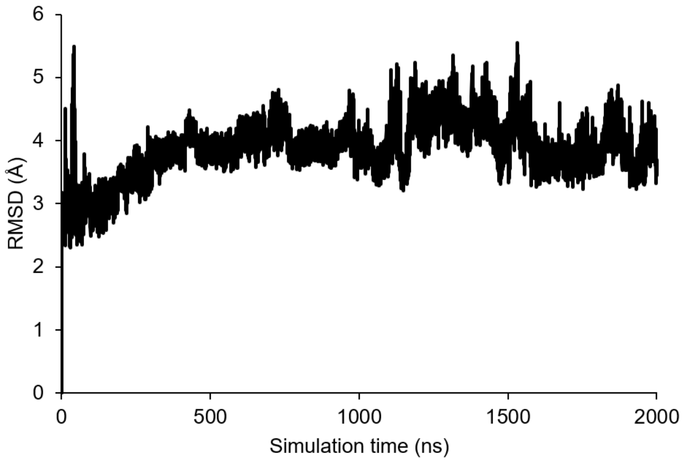

Figure S80. RMSD of Tel22-Na^+ in the intercalative **Py-Azo4F-3N***cis* Pocket 5 with respect to 143D.

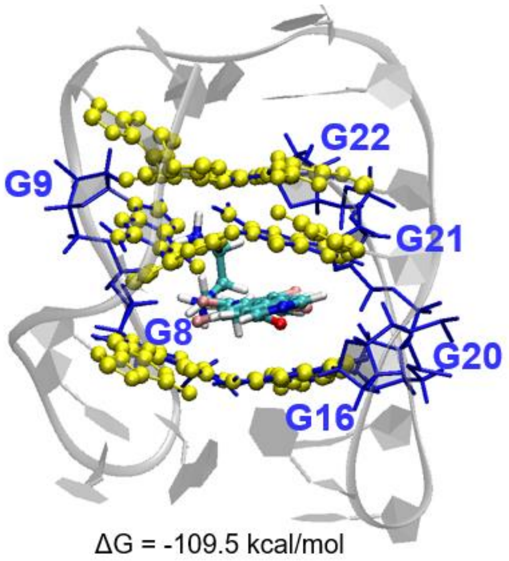

Figure S81. Representative MD structure and MMGBSA binding energy of the intercalative **Py-Azo4F-3N***trans* Pocket 1 in Tel22-Na^+. The 6 most contributing residues according to the MMGBSA analysis are indicated in blue, purple and magenta for G, T and A, respectively.

Table S11. MMGBSA energy decomposition of the 6 most contributing residues in the intercalative **Py-Azo4F-3N***trans* Pocket 1 in Tel22-Na^+. Energies in kcal/mol.

	ΔG_{vdW}	ΔG_{el}	ΔG_{pol}	ΔG_{nonpol}	ΔG_{tot}
G9	-5.96	-178.44	121.66	-3.49	-66.24
G8	-10.19	-136.46	105.14	-5.14	-46.65
G22	-7.84	-95.90	68.67	-3.33	-38.39
G21	-13.30	-75.91	62.16	-6.10	-33.16
G16	-10.57	-38.26	37.06	-5.32	-17.09
G20	-9.11	-54.28	53.17	-4.47	-14.70

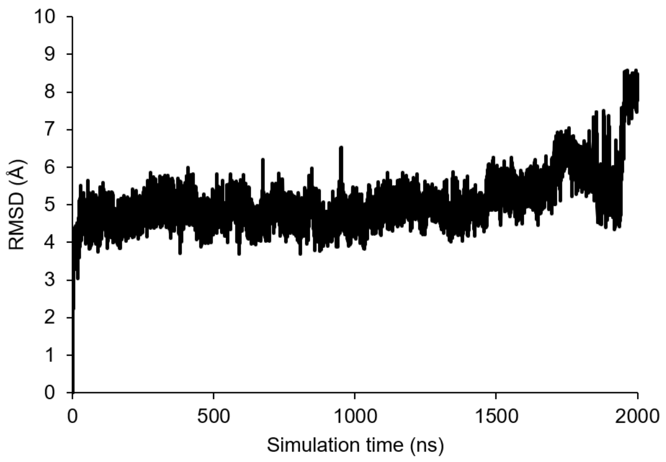

Figure S82. RMSD of **Py-Azo4F-3N** with respect to its docking pose in the intercalative **Py-Azo4F-3N**$_{trans}$ Pocket 1 in Tel22-Na^{+}.

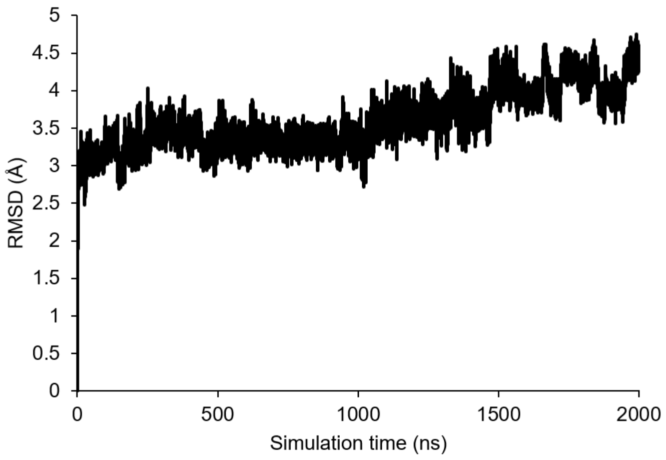

Figure S83. RMSD of Tel22-Na^{+} in the intercalative **Py-Azo4F-3N**$_{trans}$ Pocket 1 with respect to 143D.

Finally, we include the decomposition of the energy contributions per nucleotide in the most stable external binding pockets (Figure S84). As intercalation is more favourable in all the cases, the energy contributions in the intercalative pockets are discussed in the main text (Figure 5). Interestingly, for both intercalative and external poses, the Gs from the G tetrads contribute more to the G4 stabilization than the nucleotides from the grooves.

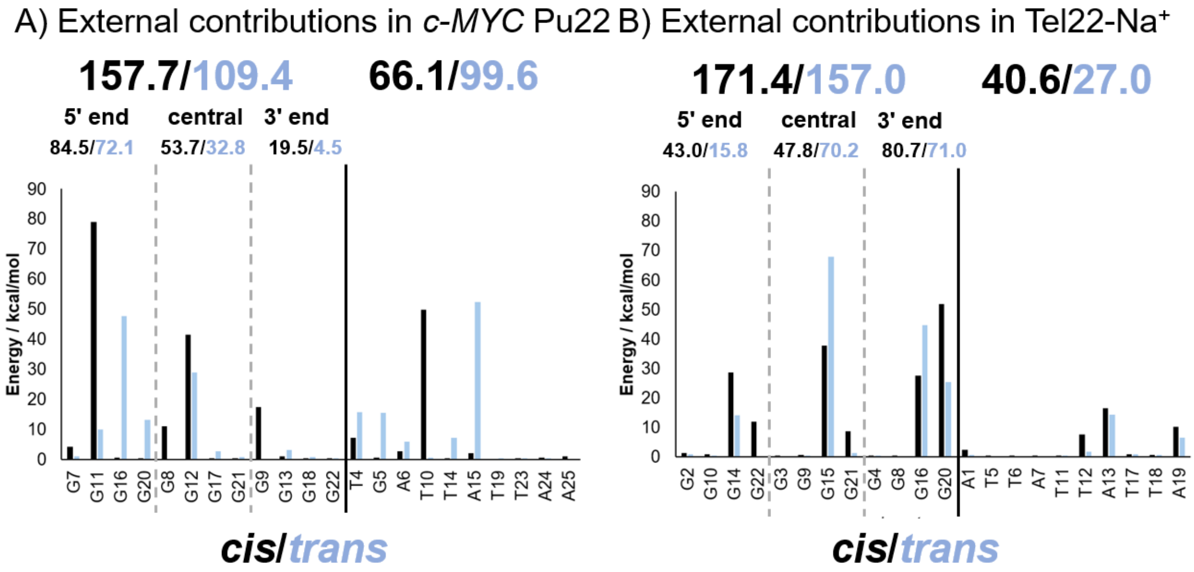

Figure S84. Decomposition of the energy contributions per nucleotide in the most stable external binding poses of **Py-Azo4F-3N** in *c-MYC* Pu22 (A) and Tel22-Na^+ (B).

7. REFERENCES

1. Bléger, D.; Schwarz, J.; Brouwer, A. M.; Hecht, S. o-Fluoroazobenzenes as Readily Synthesized Photoswitches Offering Nearly Quantitative Two-Way Isomerization with Visible Light. *J. Am. Chem. Soc.* **2012,** *134*, 20597-20600.
2. Zhao, F.; Grubert, L.; Hecht, S.; Bléger, D. Orthogonal switching in four-state azobenzene mixed-dimers. *Chem. Commun.* **2017,** *53*, 3323-3326.
3. Berman, H. M.; Westbrook, J.; Feng, Z.; Gilliland, G.; Bhat, T. N.; Weissig, H.; Shindyalov, I. N.; Bourne, P. E. The Protein Data Bank. *Nucleic Acids Res.* **2000,** *28*, 235-42.
4. Ambrus, A.; Chen, D.; Dai, J.; Jones, R. A.; Yang, D. Solution Structure of the Biologically Relevant G-Quadruplex Element in the Human c-MYC Promoter. Implications for G-Quadruplex Stabilization. *Biochem.* **2005,** *44*, 2048-2058.
5. Wang, Y.; Patel, D. J. Solution structure of the human telomeric repeat d[AG3(T2AG3)3] G-tetraplex. *Structure* **1993,** *1*, 263-282.
6. D.A. Case; K. Belfon; I.Y. Ben-Shalom; S.R. Brozell; D.S. Cerutti; T.E. Cheatham, I.; V.W.D. Cruzeiro; T.A. Darden; R.E. Duke; G. Giambasu; M.K. Gilson; H. Gohlke; A.W. Goetz; R Harris; S. Izadi; S.A. Izmailov; K. Kasavajhala; A. Kovalenko; R. Krasny; T. Kurtzman; T.S. Lee; S. LeGrand; P. Li; C. Lin; J. Liu; T. Luchko; R. Luo; V. Man; K.M. Merz; Y. Miao; O. Mikhailovskii; G. Monard; H. Nguyen; A. Onufriev; F.Pan; S. Pantano; R. Qi; D.R. Roe; A. Roitberg; C. Sagui; S. Schott-Verdugo; J. Shen; C.L. Simmerling; N.R. Skrynnikov; J. Smith; J. Swails; R.C. Walker; J. Wang; L. Wilson; R.M. Wolf; X. Wu; Y. Xiong; Y. Xue; D.M. York; Kollman, P. A., *Amber 2020*. University of California: San Francisco, 2020.
7. Mark, P.; Nilsson, L. Structure and Dynamics of the TIP3P, SPC, and SPC/E Water Models at 298 K. *J. Phys. Chem. A* **2001,** *105*, 9954-9960.
8. Galindo-Murillo, R.; Robertson, J. C.; Zgarbová, M.; Šponer, J.; Otyepka, M.; Jurečka, P.; Cheatham, T. E. III, Assessing the Current State of Amber Force Field Modifications for DNA. *J. Chem. Theory Comput.* **2016,** *12*, 4114-4127.

9. Roe, D. R.; Cheatham, T. E. III, PTRAJ and CPPTRAJ: Software for Processing and Analysis of Molecular Dynamics Trajectory Data. *J. Chem. Theory Comput.* **2013,** *9*, 3084-3095.
10. Allen, W. J.; Balius, T. E.; Mukherjee, S.; Brozell, S. R.; Moustakas, D. T.; Lang, P. T.; Case, D. A.; Kuntz, I. D.; Rizzo, R. C. DOCK 6: Impact of new features and current docking performance. *J. Comput. Chem.* **2015**, *36*, 1132-1156.
11. Dickerhoff, J.; Warnecke, K. R.; Wang, K.; Deng, N.; Yang, D. Evaluating Molecular Docking Software for Small Molecule Binding to G-Quadruplex DNA. *International Journal of Molecular Sciences* **2021**, *22*, 10801.
12. Roy Dennington; Todd Keith; Millam, J. *GaussView, Version 6.1.1*, Semichem Inc: Shawnee Mission, KS, 2019.
13. Becke, A. D. Density-functional thermochemistry. III. The role of exact exchange. *J. Chem. Phys.* **1993**, *98*, 5648-5652.
14. Lee, C.; Yang, W.; Parr, R. G. Development of the Colle-Salvetti correlation-energy formula into a functional of the electron density. *Phys. Rev. B* **1988,** *37*, 785-789.
15. Stephens, P. J.; Devlin, F. J.; Chabalowski, C. F.; Frisch, M. J. Ab Initio Calculation of Vibrational Absorption and Circular Dichroism Spectra Using Density Functional Force Fields. *J. Phys. Chem.* **1994,** *98*, 11623-11627.
16. Vosko, S. H.; Wilk, L.; Nusair, M. Accurate spin-dependent electron liquid correlation energies for local spin density calculations: a critical analysis. *Can. J. Phys.* **1980,** *58*, 1200-1211.
17. Dunning, T. H., Jr. Gaussian basis sets for use in correlated molecular calculations. I. The atoms boron through neon and hydrogen. *J. Chem. Phys.* **1989,** *90*, 1007-1023.
18. Miertuš, S.; Scrocco, E.; Tomasi, J. Electrostatic interaction of a solute with a continuum. A direct utilizaion of AB initio molecular potentials for the prevision of solvent effects. *Chem. Phys.* **1981,** *55*, 117-129.
19. Mennucci, B.; Cammi, R.; Tomasi, J. Excited states and solvatochromic shifts within a nonequilibrium solvation approach: A new formulation of the integral equation formalism method at the self-consistent field, configuration interaction, and multiconfiguration self-consistent field level. *J. Chem. Phys.* **1998,** *109*, 2798-2807.
20. Frisch, M. J.; Trucks, G. W.; Schlegel, H. B.; Scuseria, G. E.; Robb, M. A.; Cheeseman, J. R.; Scalmani, G.; Barone, V.; Petersson, G. A.; Nakatsuji, H.; Li, X.; Caricato, M.; Marenich, A. V.; Bloino, J.; Janesko, B. G.; Gomperts, R.; Mennucci, B.; Hratchian, H. P.; Ortiz, J. V.; Izmaylov, A. F.; Sonnenberg, J. L.; Williams; Ding, F.; Lipparini, F.; Egidi, F.; Goings, J.; Peng, B.; Petrone, A.; Henderson, T.; Ranasinghe, D.; Zakrzewski, V. G.; Gao, J.; Rega, N.; Zheng, G.; Liang, W.; Hada, M.; Ehara, M.; Toyota, K.; Fukuda, R.; Hasegawa, J.; Ishida, M.; Nakajima, T.; Honda, Y.; Kitao, O.; Nakai, H.; Vreven, T.; Throssell, K.; Montgomery Jr., J. A.; Peralta, J. E.; Ogliaro, F.; Bearpark, M. J.; Heyd, J. J.; Brothers, E. N.; Kudin, K. N.; Staroverov, V. N.; Keith, T. A.; Kobayashi, R.; Normand, J.; Raghavachari, K.; Rendell, A. P.; Burant, J. C.; Iyengar, S. S.; Tomasi, J.; Cossi, M.; Millam, J. M.; Klene, M.; Adamo, C.; Cammi, R.; Ochterski, J. W.; Martin, R. L.; Morokuma, K.; Farkas, O.; Foresman, J. B.; Fox, D. J. *Gaussian 16 Rev. C.01*, Wallingford, CT, 2016.
21. Woods, R. J.; Chappelle, R. Restrained electrostatic potential atomic partial charges for condensed-phase simulations of carbohydrates. *J. Mol. Struct.: THEOCHEM* **2000,** *527*, 149-156.
22. Slater, J. C. A Simplification of the Hartree-Fock Method. *Phys. Rev.* **1951,** *81*, 385-390.
23. Hehre, W. J.; Ditchfield, R.; Pople, J. A. Self—Consistent Molecular Orbital Methods. XII. Further Extensions of Gaussian—Type Basis Sets for Use in Molecular Orbital Studies of Organic Molecules. *J. Chem. Phys.* **1972,** *56*, 2257-2261.

24. Hariharan, P. C.; Pople, J. A. The influence of polarization functions on molecular orbital hydrogenation energies. *Theor. Chim. Acta* **1973,** *28*, 213-222.
25. Wang, J.; Wang, W.; Kollman, P. A.; Case, D. A. Automatic atom type and bond type perception in molecular mechanical calculations. *J. Mol. Graphics and Modell.* **2006,** *25*, 247-260.
26. Pettersen, E. F.; Goddard, T. D.; Huang, C. C.; Couch, G. S.; Greenblatt, D. M.; Meng, E. C.; Ferrin, T. E. UCSF Chimera—A visualization system for exploratory research and analysis. *J. Comput. Chem.* **2004,** *25*, 1605-1612.
27. He, X.; Man, V. H.; Yang, W.; Lee, T.-S.; Wang, J. A fast and high-quality charge model for the next generation general AMBER force field. *J. Chem. Phys.* **2020,** *153*, 114502.
28. McCullagh, M.; Franco, I.; Ratner, M. A.; Schatz, G. C. DNA-Based Optomechanical Molecular Motor. *J. Am. Chem. Soc.* **2011,** *133*, 3452-3459.
29. Humphrey, W.; Dalke, A.; Schulten, K. VMD: Visual molecular dynamics. *J. Mol. Graph.* **1996,** *14*, 33-38.
30. Genheden, S.; Ryde, U. The MM/PBSA and MM/GBSA methods to estimate ligand-binding affinities. *Expert Opin. Drug Dis.* **2015,** *10*, 449-461.
